# Supplementary material for: Balancing Honest Assessment and Compassion for Learners Experiencing Burnout: A Workshop and Feedback Tool for Clinical Teachers
Source: MedEdPORTAL. 2024 Oct 15;20:11449. doi: 10.15766/mep_2374-8265.11449 (PMC11473647; doi:10.15766/mep_2374-8265.11449)
Supplement: Supplementary file 1 — GetINburnOUT Method.pdfAgenda.docxFacilitator Guide.docxWorkshop Presentation.pptxCases.docxOnline Workshop Evaluation.pdf [file mep_2374-8265.11449-s001.zip › A. GetINburnOUT Model.pdf]

GetINBurnOUT

This tool is to help clinical educators to deliver feedback to learners in the clinical setting who may be experiencing burnout. Using this model, the educator can support their learner while still delivering honest and accurate feedback to correct deficiencies and promote growth.

Identify

- **Validated screening questions:**
  - Are you feeling burned out from your work?
  - Have you become more callous toward people since you started this job/ rotation?
- **OR frame concern in reference to observed behaviors or interactions.**
  - *e.g. "I've noticed (behavior, perceived attitude, etc) and I'm wondering if you're feeling overwhelmed?"*
- **<u>Briefly</u> explore impact and perception of these feelings**

Name & Normalize

- **Be ready to explain burnout**
  - *e.g. "The feelings you are explaining sound like maybe you are experiencing burnout. Does that resonate with you?"*
- **Normalize burnout**
  - e.g. *"The hospital wards are overwhelming, and burnout is especially common among residents this time of year."*
- **Empathize:**
  - e.g. *"I am sorry you are feeling burned out. It makes it really hard to get through a day."*

Ask-Tell-Ask

(i.e. deliver feedback)

- **Verbalize moving into feedback**
  - Promise to return to burnout
  - If learner persists on the topic, discuss impact on work
  - If comes up mid-session, backup and start with "IN"
- **General feedback principles**
  - Plan ahead
  - Avoid overloading (1-2 items)
  - Behavior-based only/avoid nonactionable comments
- **Give honest feedback**
  - Address deficiencies
  - Include positives when present

BurnOUT

(Return to topic & provide intervention)

- Clarify expectations and adjust leaner's goals
- Refer to leadership (be upfront if escalating concerns)
- Encourage support seeking in and out of medicine
- Set <u>ONE</u> self-care goal
- Discuss self-monitoring of crossover from work → life

Guiding principles:

*** Be prepared * Address deficiencies * Brief intervention***
