# Supplementary material for: Balancing Honest Assessment and Compassion for Learners Experiencing Burnout: A Workshop and Feedback Tool for Clinical Teachers
Source: MedEdPORTAL. 2024 Oct 15;20:11449. doi: 10.15766/mep_2374-8265.11449 (PMC11473647; doi:10.15766/mep_2374-8265.11449)
Supplement: Supplementary file 1 — GetINburnOUT Method.pdfAgenda.docxFacilitator Guide.docxWorkshop Presentation.pptxCases.docxOnline Workshop Evaluation.pdf [file mep_2374-8265.11449-s001.zip › B. Agenda.docx]

**Appendix B: Workshop Agenda**

| Timing | Agenda Item | Activity Type | Objective # addressed |
| --- | --- | --- | --- |
| 0-5 min | **Introductions & Engagement** | **Introductions of group**  **Individual Reflection (invite to share):** “Consider a time you worked with a learner you suspected was burned out…” |  |
| 10-25 min | **Conceptualizing burnout in medical trainees** | **Large Group Game:** teams vote True/False and explain reasoning  **For virtual format, virtual polling of true/false questions* | 1,2 |
| 25-30 min | **Foundational Definitions and Introduction to Maslach Burnout Inventory (MBI)** | **Didactic:** Introduce the MBI as a framework for conceptualizing burnout in others/learners | 1,2 |
| 30-50 min | **MBI application to the Clinical Learning Environment (CLE)** | **Small Group Activity:** Brainstorm behaviors on sticky notes  **Large Group Activity:** Groups sort behaviors into MBI facets  **For virtual format, use virtual open ended activity within each MBI facet and brainstorm* | 2 |
| 50-55 min | **The Evidence: behaviors of learners experiencing burnout** | **Didactic:** Summary of evidence  **Individua/Group:** “Does this evidence change your approach to learners in the CLE…”. | 2 |
| 55-60 min | **Break** | | |
| 60-50 minutes | **The GetINBurnOUT method** | **Didactic:** Introduce GetINBurnOUT method and intervention with time for questions | 3 |
| 70-85  min | **Practicing GetINBurnOUT method in the CLE** | **Small Group Case Practice:** Review manifestations as a group, practice approaching the trainee using GetINBurnOUT method  **Report to Large group:** Cases illustrate different types of trainee burnout for discussion  **For virtual format, use breakout rooms for small group to discuss cases, and return to large group for presentation/sharing* | 1,2,3 |
| 85-90 min | **Wrap Up** | A note on summative evaluations  Thank you and evaluation |  |
