# Supplementary material for: Balancing Honest Assessment and Compassion for Learners Experiencing Burnout: A Workshop and Feedback Tool for Clinical Teachers
Source: MedEdPORTAL. 2024 Oct 15;20:11449. doi: 10.15766/mep_2374-8265.11449 (PMC11473647; doi:10.15766/mep_2374-8265.11449)
Supplement: Supplementary file 1 — GetINburnOUT Method.pdfAgenda.docxFacilitator Guide.docxWorkshop Presentation.pptxCases.docxOnline Workshop Evaluation.pdf [file mep_2374-8265.11449-s001.zip › C. Facilitator Guide.docx]

**Appendix C**

**Balancing Honest Assessment and Compassion for Learners Experiencing Burnout: A Workshop and Feedback Tool for Clinical Teachers**

**Facilitator Guide**

**Slides 0-4 (3 min)**

Introduction

- Presenters introduce themselves and the workshop. In person, participants should be asked to introduce themselves within their groups.
- Review the Goal and Objectives as written.

**Slide 5** (2 min)

Self-reflection

- Guide the group through self-reflection to prime them for the workshop. Ask them to consider a time they may have had a learner that was burned out. What made them think the learner was burnout out? What did they do? Did it impact the way they evaluated the learner or provided feedback to the learner?
- After the reflection, suggest to the participants that they keep this learner or particular interaction in mind throughout the workshop.

**Slides 6-20** (20 min)

Conceptualizing burnout in medical trainees

- These slides review common myths related to burnout through True or False questions.
- In person, statements were provided on each slide. Participants were asked to discuss quickly with their group and vote using their green (true) and red (false) “flags.”
- In the virtual format, this was an individual activity completed using PollEverywhere® software via multiple choice questions with A (true) or B (false). Depending on time, groups/individuals were asked to explain their votes before answers are revealed to elicit any additional misconceptions that needed to be addressed. Each poll or question is followed by the correct answer along with an explanation and slight expansion on the subject.
- Additional supporting information and references can be found in the footnotes.

**Slides 21-22** (5 min)

Foundational definitions and introduction to the Maslach Burnout Inventor

- This section is intended to introduce the Maslach burnout inventory, the different dimensions of burnout, and how it can be used a framework for conceptualizing burnout in others in the clinical learning environment.
- It should be emphasized that while MBI is intended to be an introspective tool to measure burnout, in this workshop, the MBI would use be used as a framework for identifying burnout in others.
- Given that the MBI is foundation for individuals interested in burnout and wellness across fields, time must be taken to answer questions and ensure understanding.

**Slide 23-24**(20 min)

MBI application to the clinical learning environment & common behaviors of learners experiencing burnout

- Begin by having participants separate into small groups of approximately 5 individuals (group sizing can be modified based on number of participants but should probably have no more than 6 per group) ​
- Ask groups to brainstorm behaviors or signs that may reflect one of the three burnout dimensions.
- In the in-person format, ask participants to write down one behavior per sticky note and give approximately 10 minutes for this activity). Then, ask the groups to categorize these behaviors into the three MBI domains. A representative (or 3 representatives) should place the appropriate sticky notes onto one of three large sheets of paper, with each sheet labeled with one domain. Then, the facilitators take turns to read within each domain and the large group is invited to comment.
- In the virtual format, open text polling or the chat function was used to brainstorm behaviors in each domain one at a time.
- Invite discussion on the overlap of behaviors across multiple domains of the MBI but use the next slide (slide 24) to show behaviors of burned-out trainees from the evidence, which is minimal. Continue to invite discussion on how the evidence compares to the group’s brainstormed signs of burnout, which are often less specific and less behavior-based and given opportunity to reflect.

**Slides 25** (5 min)

- This slide will acknowledge how difficult it can be to differentiate between burnout and depression and explore their link and commonalities.
- Facilitators should take special care to control the discussion at this point to remain within the scope of the workshop (as the goal is not to teach comprehensive psychiatric diagnostics or the nuances of system-level support for mentally ill learners). These types of concerns should be escalated to programmatic leadership.

**Slides 27-30 (2 minute)**

Assessment and Barriers

- These slides begin by defining and reviewing the different types of assessment and common barriers to providing honest assessment with evidence

**Slide 31-38 (8 minute)**

Introduction of the GetINBurnOUT Method

- Facilitators will then introduce the GetINBurnOUT method to provide honest but compassionate assessment to a learner. Print out The GetINBurnOUT method (Appendix A) for participants to reference throughout.
- Slide 31 will provide an overview of the method and what it stands for. It was created as a modification of the ask-tell-ask feedback format to include identification of burnout, naming and normalizing burnout, and then lastly acknowledging the burnout, having compassion for the learner, and if needed referring to program leadership for additional help.
- Each of the three parts of the GetINBurnOUT method is described in detail sequentially and facilitators provide suggestions on how to carry out each part:
  - (1) The validated screening tools are taught as well as more pointed questions. Participants then were taught to use knowledge gained in the workshop to “name and normalize” burnout, including its definition and frequency in healthcare.
  - (2) Participants are empowered to fully move on from the topic of burnout to provide feedback by verbalizing that this transition is happening and providing reassurance that they will return to the topic before the end of the session. While this workshop is not intended to train educators in effective feedback, a brief overview of best practice feedback techniques was reviewed including how to be prepared, focusing on behavior-based and actionable items, and choosing only 1-2 items each session. These tips are essential in the setting of learner burnout which is an obstacle to honest feedback.
  - (3) Participants then are prepared to deliver the intervention. It can be discussed that this intervention is built on burnout evidence by wellness experts, but there is no evidence on its usefulness for learners at this point in time. However, it allows the clinical educator to take some action while maintaining their time-limited role to the learner.
    - Step 1. Clarifying expectations and adjusting goals. Explain this aims to address universal contributors to burnout (workload, time pressure, lack of role clarity) as well as reminds trainees that they have support/are not the last stop for difficult clinical scenarios.
    - Step 2. Assessing the need for referral to leadership. Leadership may help with scheduling, resources, or arranging coaching/mentorship depending on the learner’s specific needs. Reiterate that any concern for major burnout or other mental health issues should be referred to leadership. Remind the audience to be honest with the resident about intention to escalate concerns if doing so.
    - Step 3. Encouraging support seeking. There is evidence that social support is a mitigating factor for burnout. This includes having support both within the medical community (peers, mentors) and outside of the medical community.
    - Step 4. Setting one self-care goal. Like in high quality feedback, goals should be realistic, timely, and specific. While ideally multiple goals could be set and achieved, this would be a chance to set one (exercise, social time, better sleep, task sharing, setting mentorship meetings) and determine a timeline for such.
    - Step 5. Discussing self-monitoring for development of other mental health disturbance. Refer back to the discussion of depression vs burnout. Remind the audience that medical trainees are adult learners and should be empowered to understand when burnout moves out of the occupation setting.
- Opportunity to ask questions.

**Slides 39-42**(20 min)

Application of the GetINBurnOUT method in the clinical learning environment through practice cases

- Divide back into small groups to practice applying the GetINBurnOUT method. Again here, groups of 5-6 are ideal. Groups can be the same as earlier in the workshop. Breakout rooms can be used in the virtual format.
- Assign one case to each group. Each case represented a different “type” of learner at risk of or suffering from burnout: a previously high-performing learner who performance is impacted by burnout, a high-performing learner at risk of burnout whose performance is not impacted, and a learner in difficulty whose competency cannot be directly attributed to burnout. It is ok for multiple groups to have the same case as long as all cases are used by at least one group.
- For each case, participants should answer the following questions:
  - What behaviors seem consistent or inconsistent with burnout?
  - Is burnout affecting the learner’s performance?
  - How can you use the GetINBurnOUT method with them?
- Case 1 explores an experience with a resident whose interpersonal interactions have deteriorated over the course of the past academic year in comparison to what they were when she started.
  - Discuss how these behaviors and attitude are an obvious change from previous which can signal burnout and/or mental health issues.
  - Behaviors consistent with burnout include snapping at a nurse during phone call, being curt in interactions with care manager (represents emotional exhaustion), stating “that’s not my job” about tracking records (represents depersonalization as she removing self from patient care role and decreased sense of personal accomplishment as she does not feel worth in those tasks).
  - In addition to delivering feedback about the above behaviors, acknowledge that learner still interacts well with families during rounds and work is thorough and efficient despite other concerns so performance is not impacted across the board.
  - Reflect on information learned in True/False activity that compassionate and advanced communicators may even be at greater risk of burnout.
  - The GetINBurnOUT method will be specifically useful in determining if burnout is present and helping the resident understand their own burnout and its potential impact on performance. In this case, it might be appropropriate to contextualize these competency issues (interpersonal skills) with the understanding that burnout is present. However, the learner must still be corrected and guided in the feedback portion.
  - The burnout intervention can help this mature learner track their own burnout and any concerns for cross-over to depression and provide them with an opening to seek support from program leadership.
- Case 2 explores an experience with a resident who is performing well but openly appears exhausted, has given up social activities, and regularly stays late at the hospital or and works past the end of his shift.
  - Discuss that there are no obvious behaviors suggesting emotional exhaustion, depersonalization, or decreased sense of personal accomplishment.
  - This case represents a trainee at high risk of burnout and an opportunity to intervene before behaviors develop. In returning to knowledge gained in the True/False activity, risk of burnout increases with high workload, lack of role clarity and, in medical trainees, lack of work-life balance.
  - The GetINBurnOUT can be used to screen for feelings of burnout, especially using validated screening questions.
  - Whether or not screening questions point to burnout, positive feedback can be delivered. The intervention should focus on the educators’ concern about the resident’s risk of burnout including the evidence-based reasons mentioned above and discussion of how future burnout may impact this resident’s goals.
  - The burnout intervention should include role clarification and expectation setting, as this resident seems to be taking on tasks that should be delegated to other team members (taking on intern frontline provider role instead of supervising them to complete the task on their own, not signing out tasks). Given what the educator knows about this trainee’s priorities to socialize with colleagues, a specific, measurable self-care goal to re-establish work-life balance will be especially helpful.
- Case 3 explores an experience with a resident who endorses burnout when given critical feedback about his performance and medical knowledge.
  - It can be discussed that it is often difficult to separate if poor performance is due to burnout or if burnout is due to self-awareness of poor performance. Likely, it is a positive feedback loop and trainees who have underlying competency issues are at high risk of burnout (as well as depression or other mental health issues.)
  - In this case, the trainee demonstrates behaviors that are consistent with burnout including relying on upper level to fill in (decreased personal accomplishment) or rejection of feedback (emotional exhaustion). The literature (Baer TE et al, slide 24) also describes not fully answering patient’s questions as a specific burnout behavior. He also readily identifies/admits to feeling burnout during his feedback session and attributes his poor performance to such.
  - However, the observation of consistent suboptimal performance including inability to explain thought process demonstrates likely lack of medical knowledge and reasoning that cannot be explained by emotional exhaustion, depersonalization, or decreased personal accomplishment. This is why it is important for an educator in the CLE to take opportunities for trainees to demonstrate competency via different methods, like asking them to explain their reasoning. (It may be appropriate for facilitators to address with participants that difficulty processing may be a sign of depression.)
  - The GetINBurnOUT method is particularly useful in this case to empower the educator to empathize with the learner, but to ensure that feedback on the specific behaviors/competency issues is delivered. The educator should feel empowered to completely move on from the topic of burnout to do so, while making a plan to address competency issues beyond the resident excusing them based on their own feelings of burnout. The intervention for this trainee is especially important to refer the trainee to leadership (and disclose an intention to reach out to leadership on their behalf).
- After 15 minutes of discussion, each group can elect a representative to share the main points of their discussion. The facilitators offered a summary of key points and asked for questions or comments from the participants.

**Slide 43** (1 min)

A note on summative assessment

- Briefly mention some tips for providing good summative assessment.

**Slide 44-45** (1 min)

Thank you, evaluation, and resources

- Feel free to insert a link or QR code for an evaluation here.
- The last slide provides several resources and articles used in the creation of the workshop.
