# Supplementary material for: Balancing Honest Assessment and Compassion for Learners Experiencing Burnout: A Workshop and Feedback Tool for Clinical Teachers
Source: MedEdPORTAL. 2024 Oct 15;20:11449. doi: 10.15766/mep_2374-8265.11449 (PMC11473647; doi:10.15766/mep_2374-8265.11449)
Supplement: Supplementary file 1 — GetINburnOUT Method.pdfAgenda.docxFacilitator Guide.docxWorkshop Presentation.pptxCases.docxOnline Workshop Evaluation.pdf [file mep_2374-8265.11449-s001.zip › D. Workshop Presentation.pptx]

## Slide 1
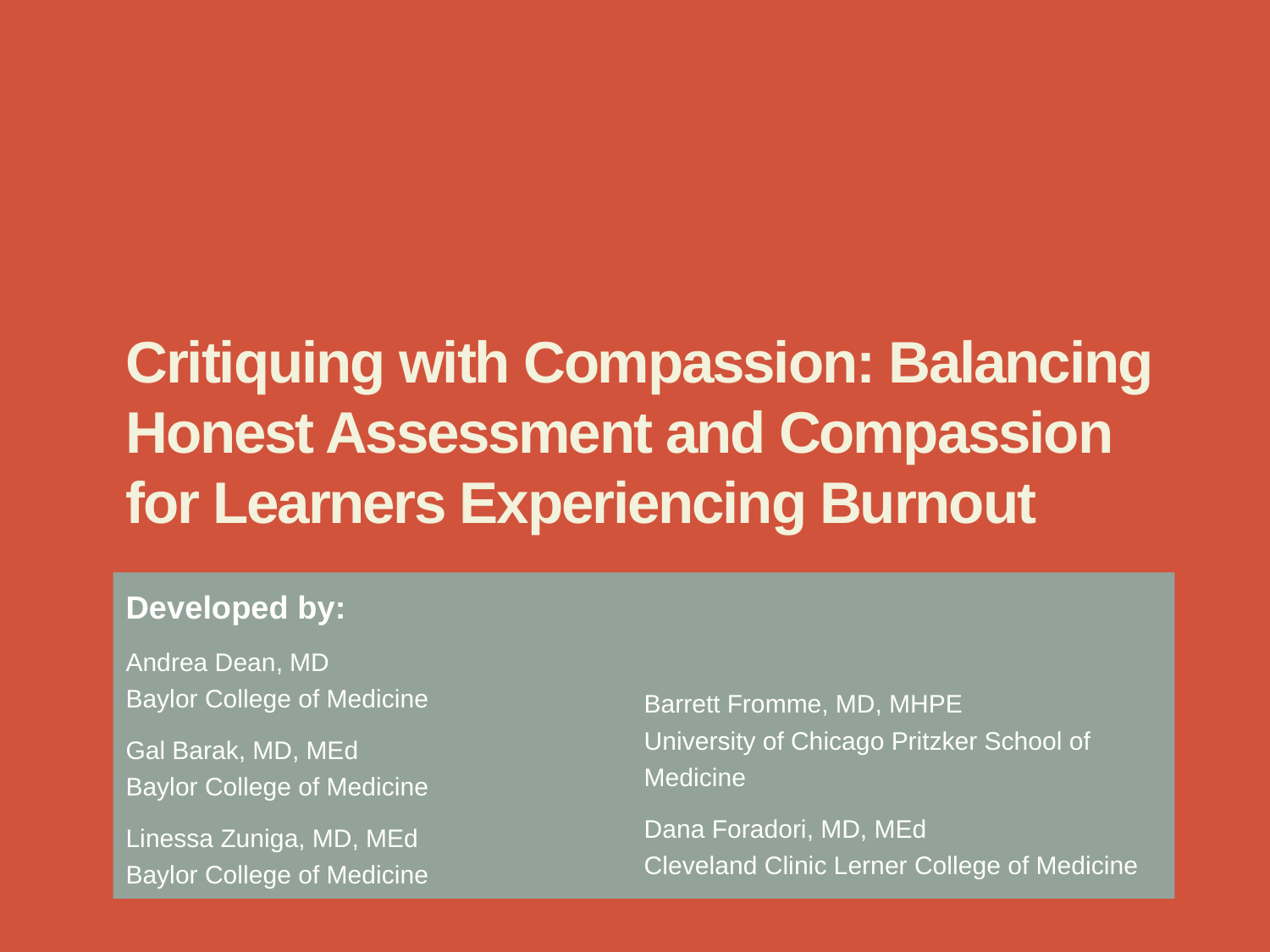

# Critiquing with Compassion: Balancing Honest Assessment and Compassion for Learners Experiencing Burnout
Developed by:
Andrea Dean, MD
Baylor College of Medicine
Gal Barak, MD, MEd
Baylor College of Medicine
Linessa Zuniga, MD, MEd
Baylor College of Medicine
Barrett Fromme, MD, MHPE 
University of Chicago Pritzker School of Medicine
Dana Foradori, MD, MEd
Cleveland Clinic Lerner College of Medicine

## Slide 2
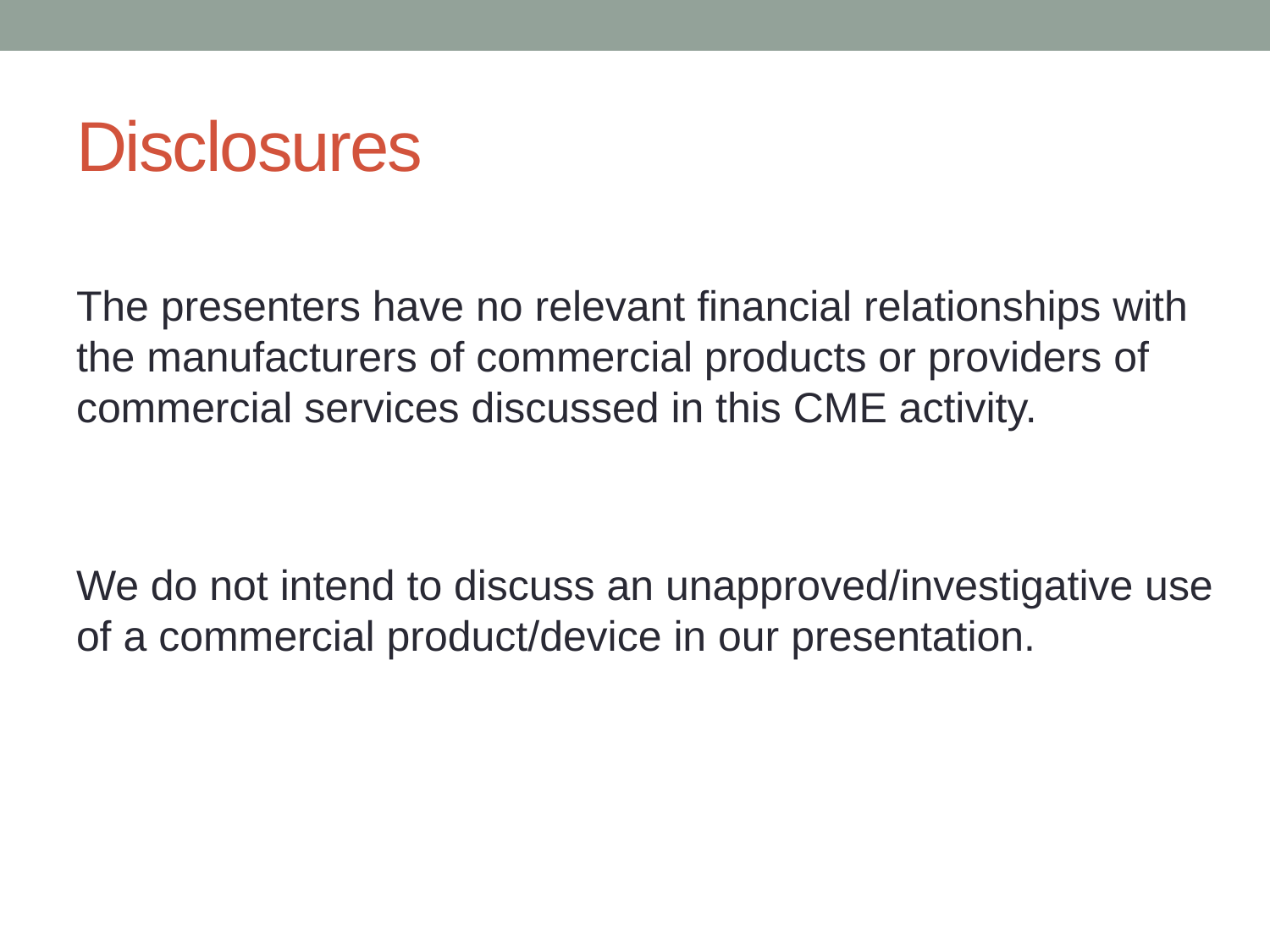

# Disclosures
The presenters have no relevant financial relationships with the manufacturers of commercial products or providers of commercial services discussed in this CME activity.
We do not intend to discuss an unapproved/investigative use of a commercial product/device in our presentation.

## Slide 3
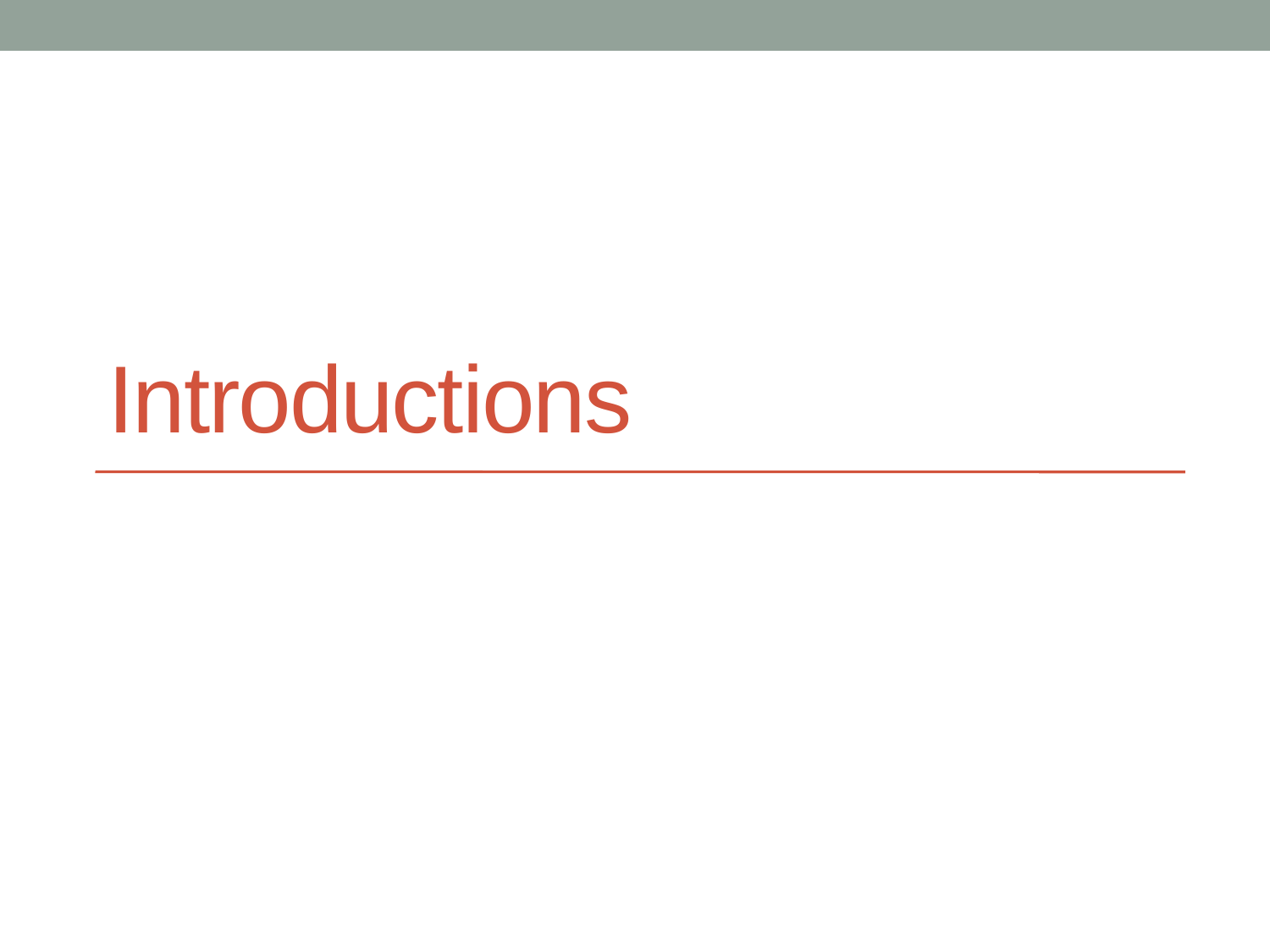

# Introductions

## Slide 4
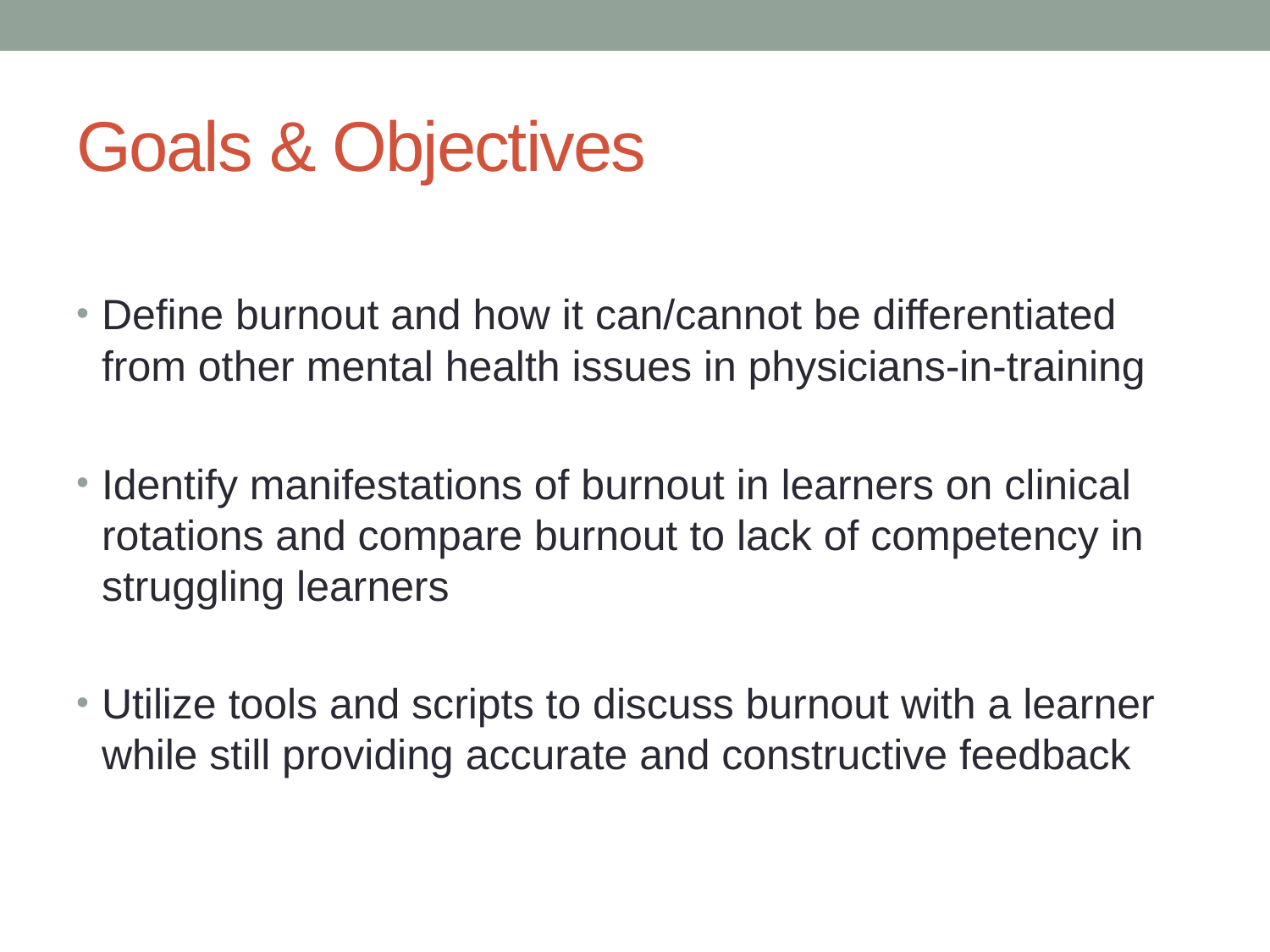

# Goals & Objectives
Define burnout and how it can/cannot be differentiated from other mental health issues in physicians-in-training
Identify manifestations of burnout in learners on clinical rotations and compare burnout to lack of competency in struggling learners
Utilize tools and scripts to discuss burnout with a learner while still providing accurate and constructive feedback

## Slide 5
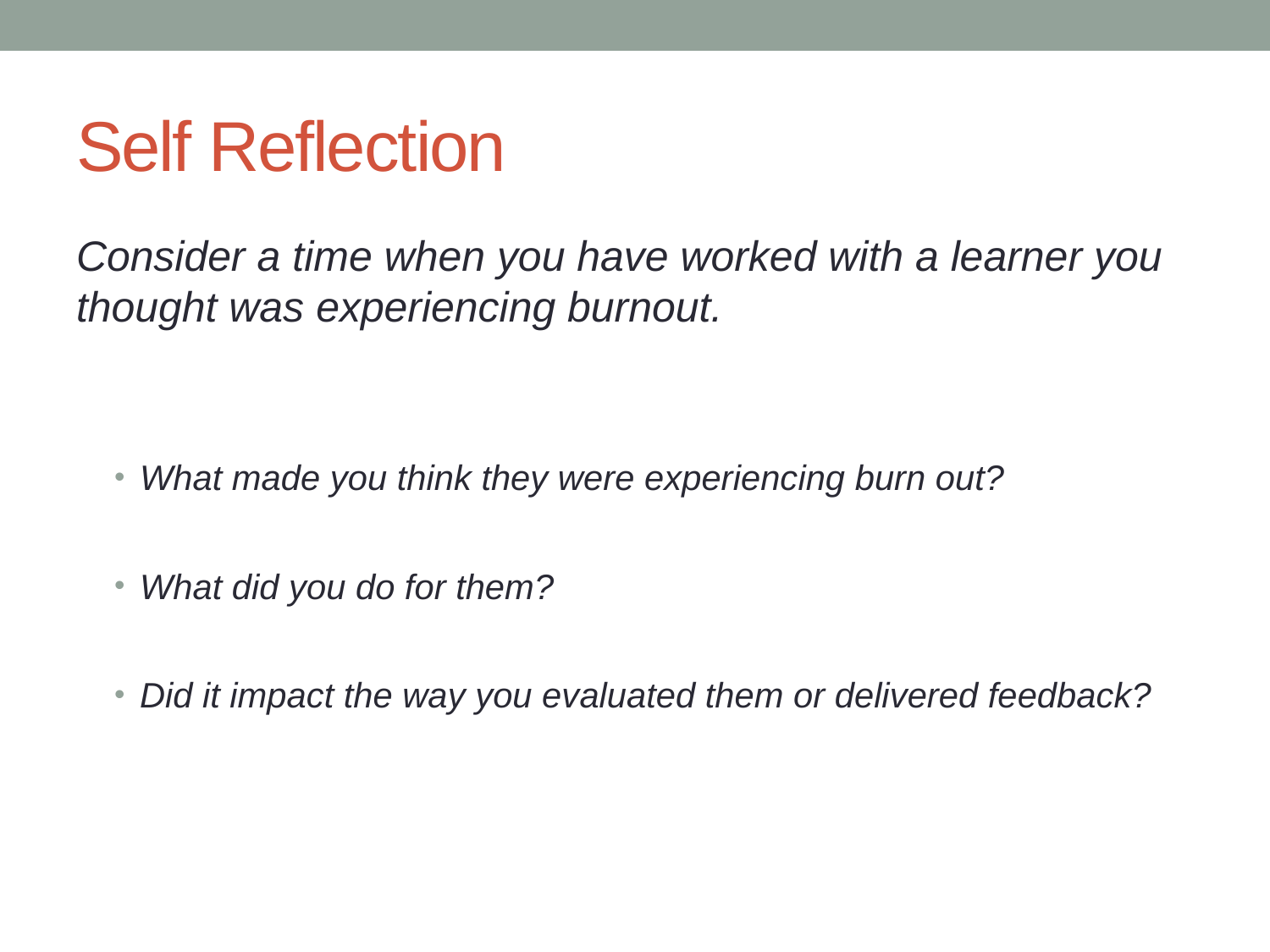

# Self Reflection
Consider a time when you have worked with a learner you thought was experiencing burnout.
What made you think they were experiencing burn out?
What did you do for them?
Did it impact the way you evaluated them or delivered feedback?

## Slide 6
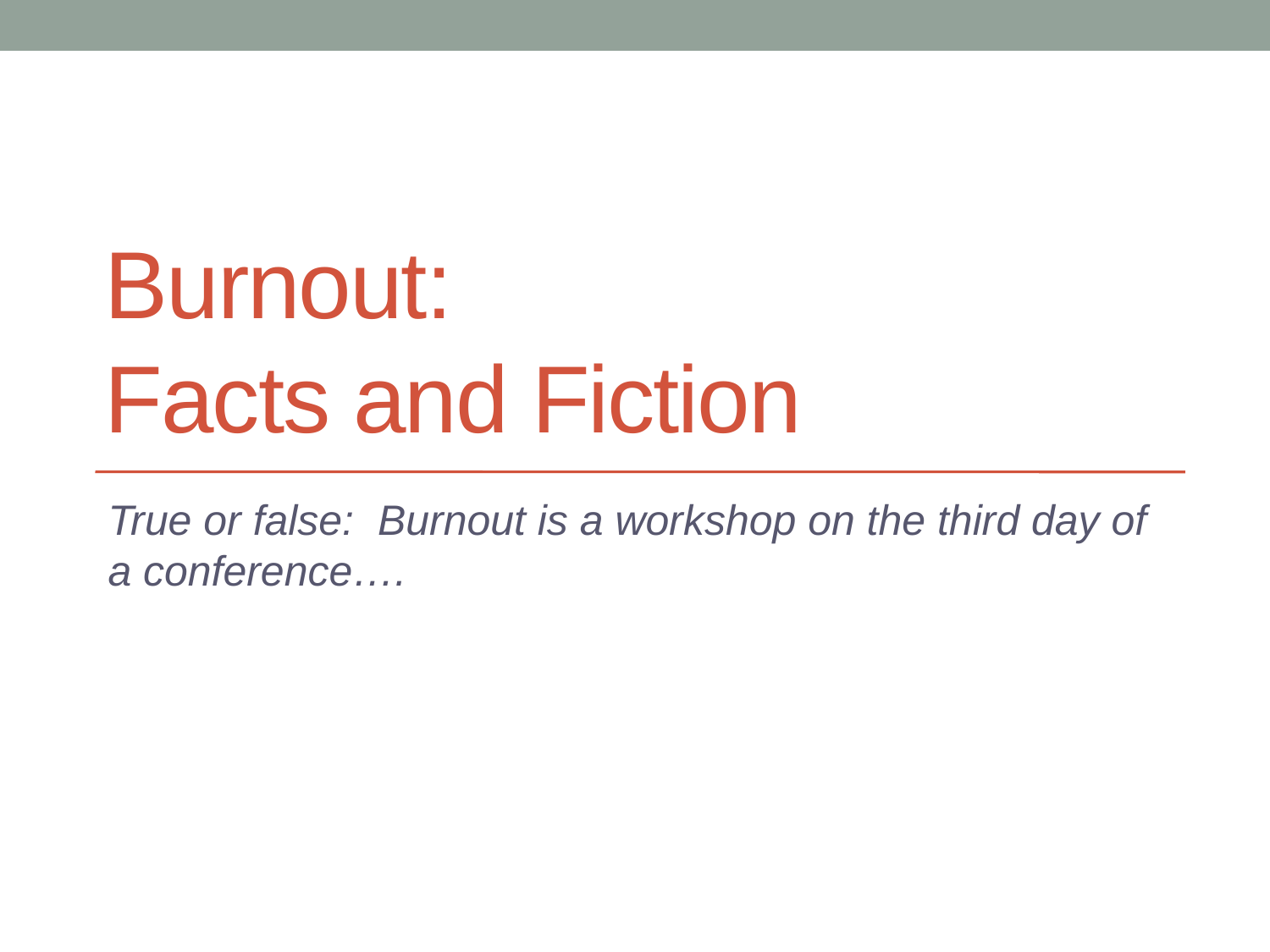

# Burnout: Facts and Fiction
True or false: Burnout is a workshop on the third day of a conference….

## Slide 7
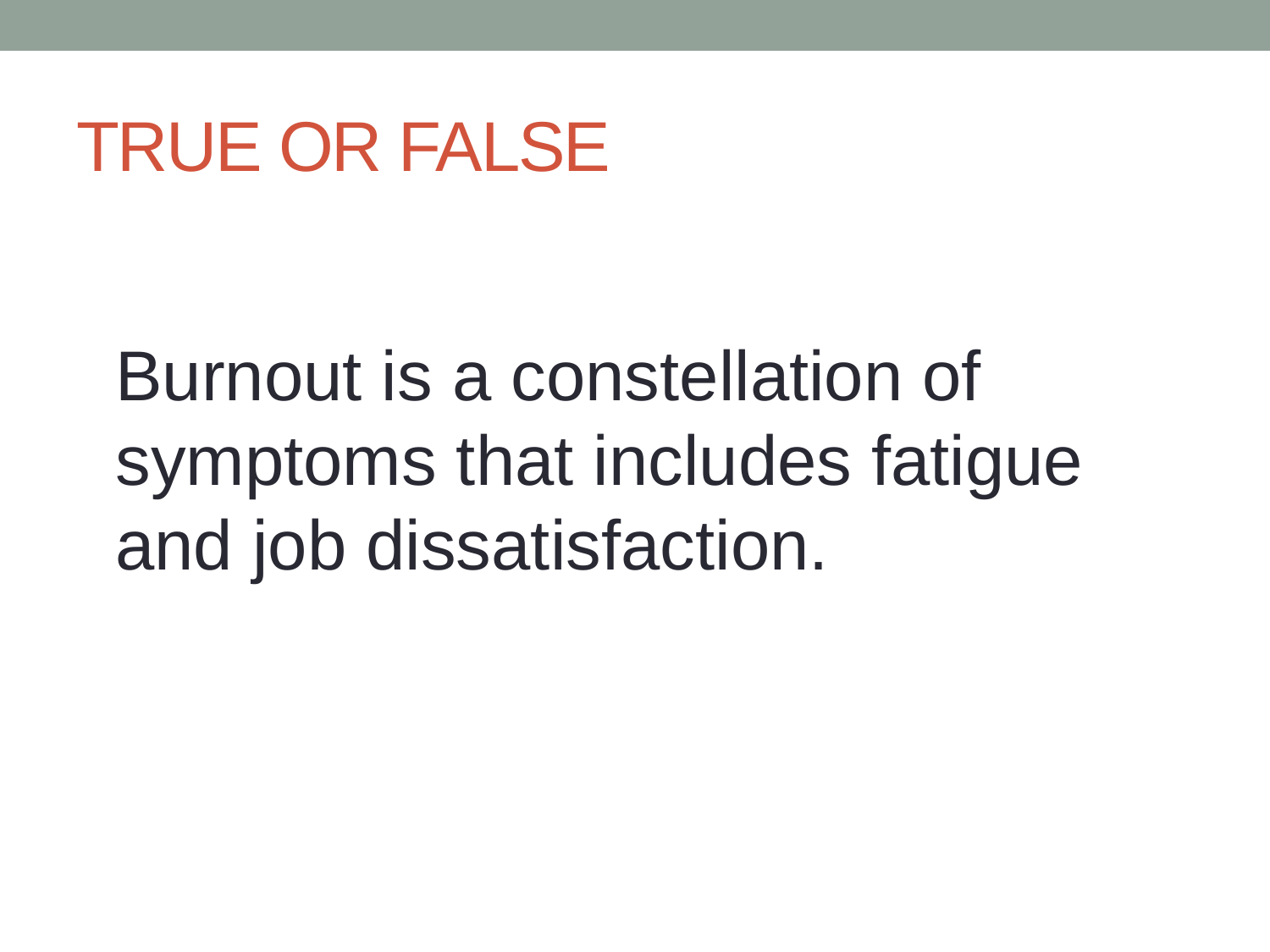

# TRUE OR FALSE
Burnout is a constellation of symptoms that includes fatigue and job dissatisfaction.

## Slide 8
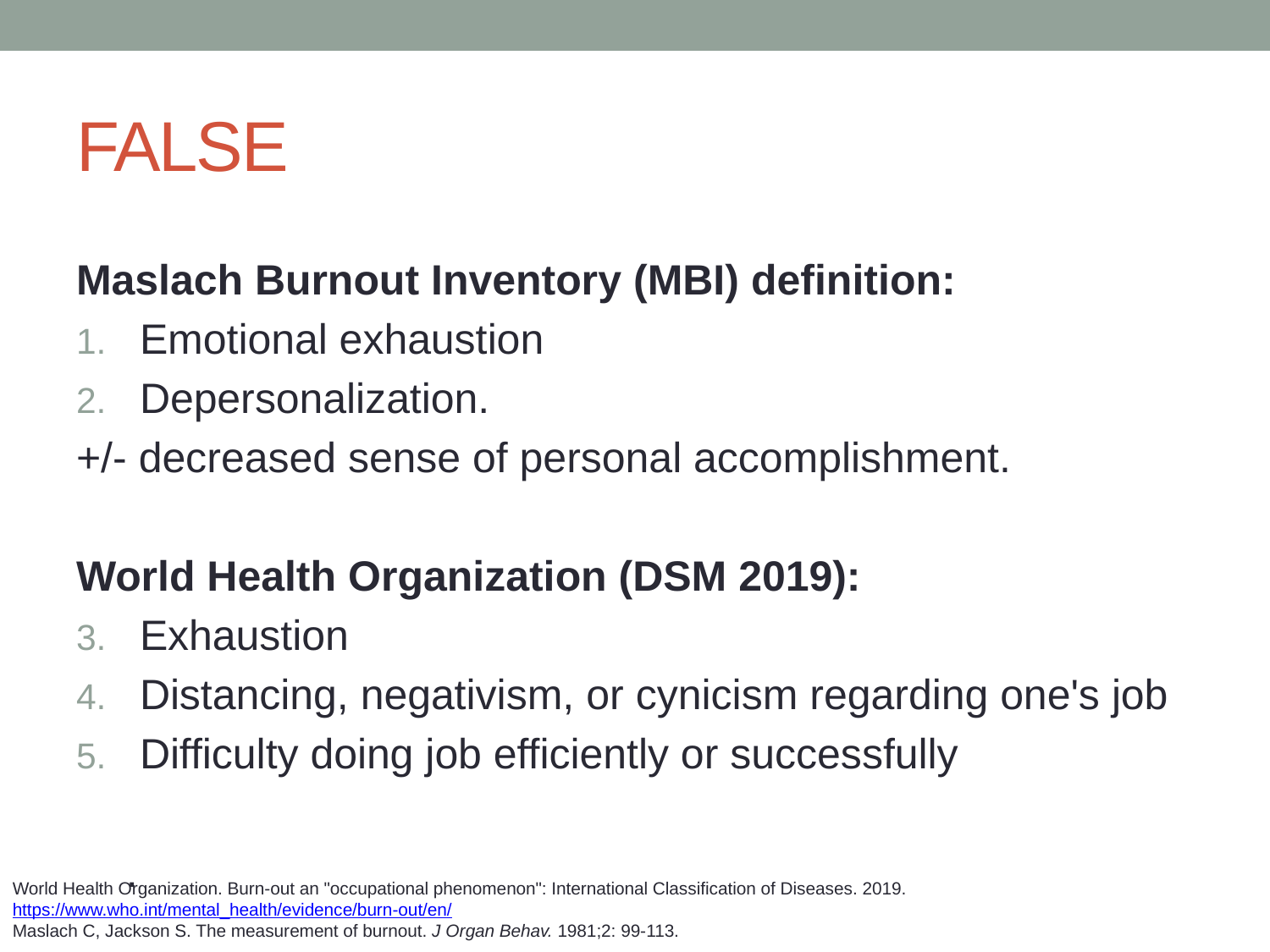

# FALSE
Maslach Burnout Inventory (MBI) definition:
Emotional exhaustion
Depersonalization.
+/- decreased sense of personal accomplishment.
World Health Organization (DSM 2019):
Exhaustion
Distancing, negativism, or cynicism regarding one's job
Difficulty doing job efficiently or successfully
 .
World Health Organization. Burn-out an "occupational phenomenon": International Classification of Diseases. 2019. https://www.who.int/mental_health/evidence/burn-out/en/
Maslach C, Jackson S. The measurement of burnout. J Organ Behav. 1981;2: 99-113.

## Slide 9
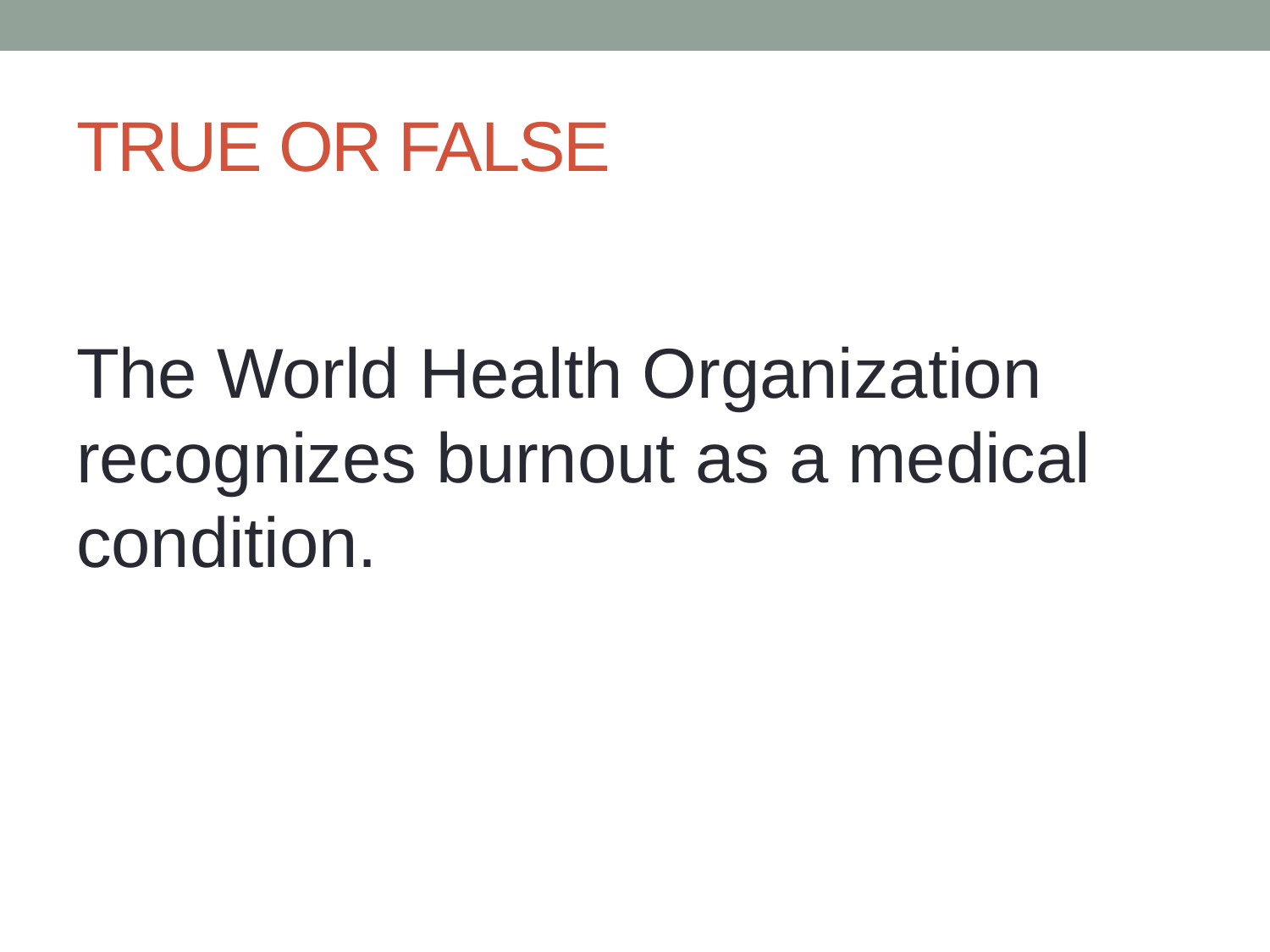

# TRUE OR FALSE
The World Health Organization recognizes burnout as a medical condition.

## Slide 10
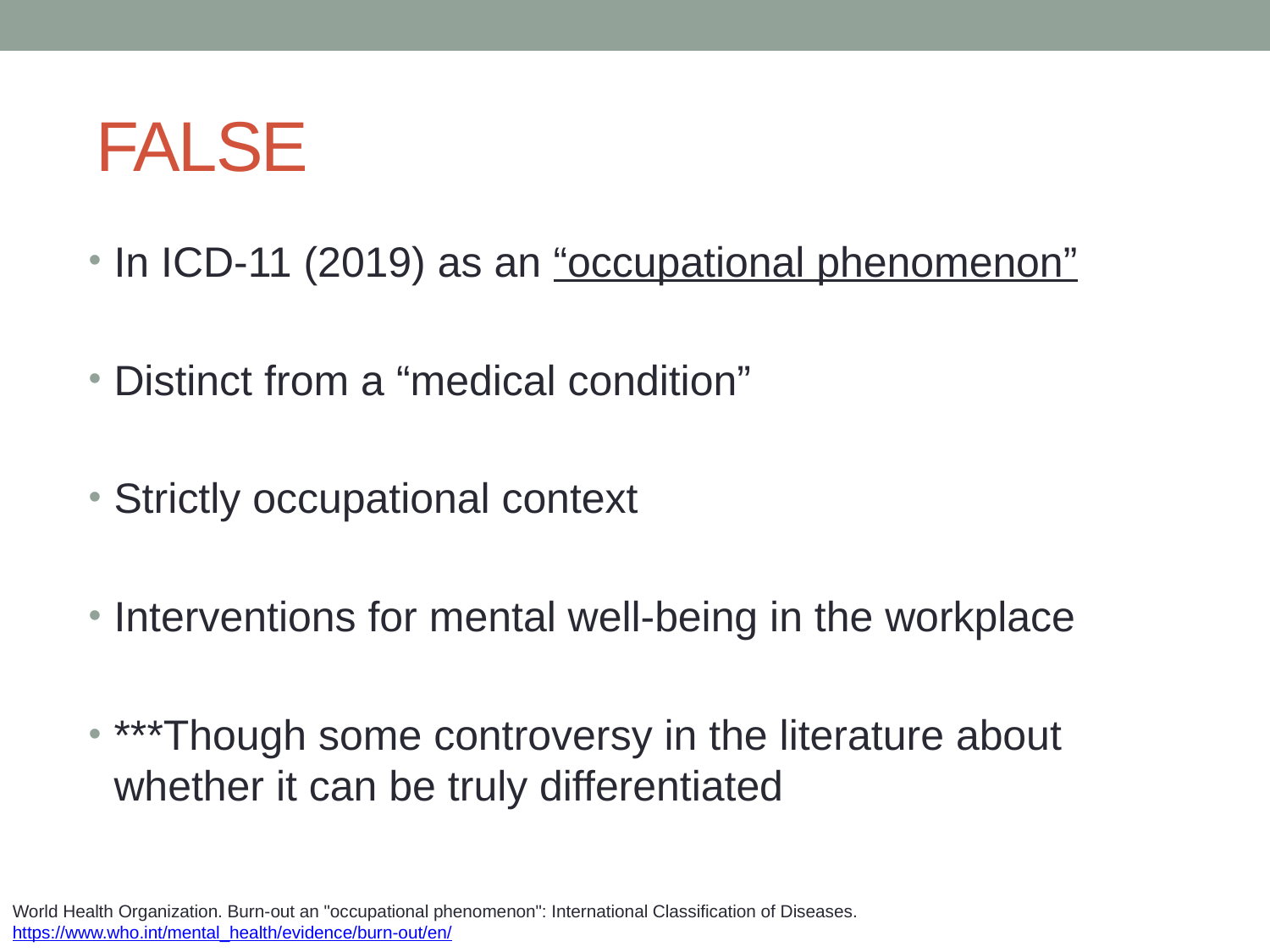

# FALSE
In ICD-11 (2019) as an “occupational phenomenon”
Distinct from a “medical condition”
Strictly occupational context
Interventions for mental well-being in the workplace
***Though some controversy in the literature about whether it can be truly differentiated
World Health Organization. Burn-out an "occupational phenomenon": International Classification of Diseases. https://www.who.int/mental_health/evidence/burn-out/en/

## Slide 11
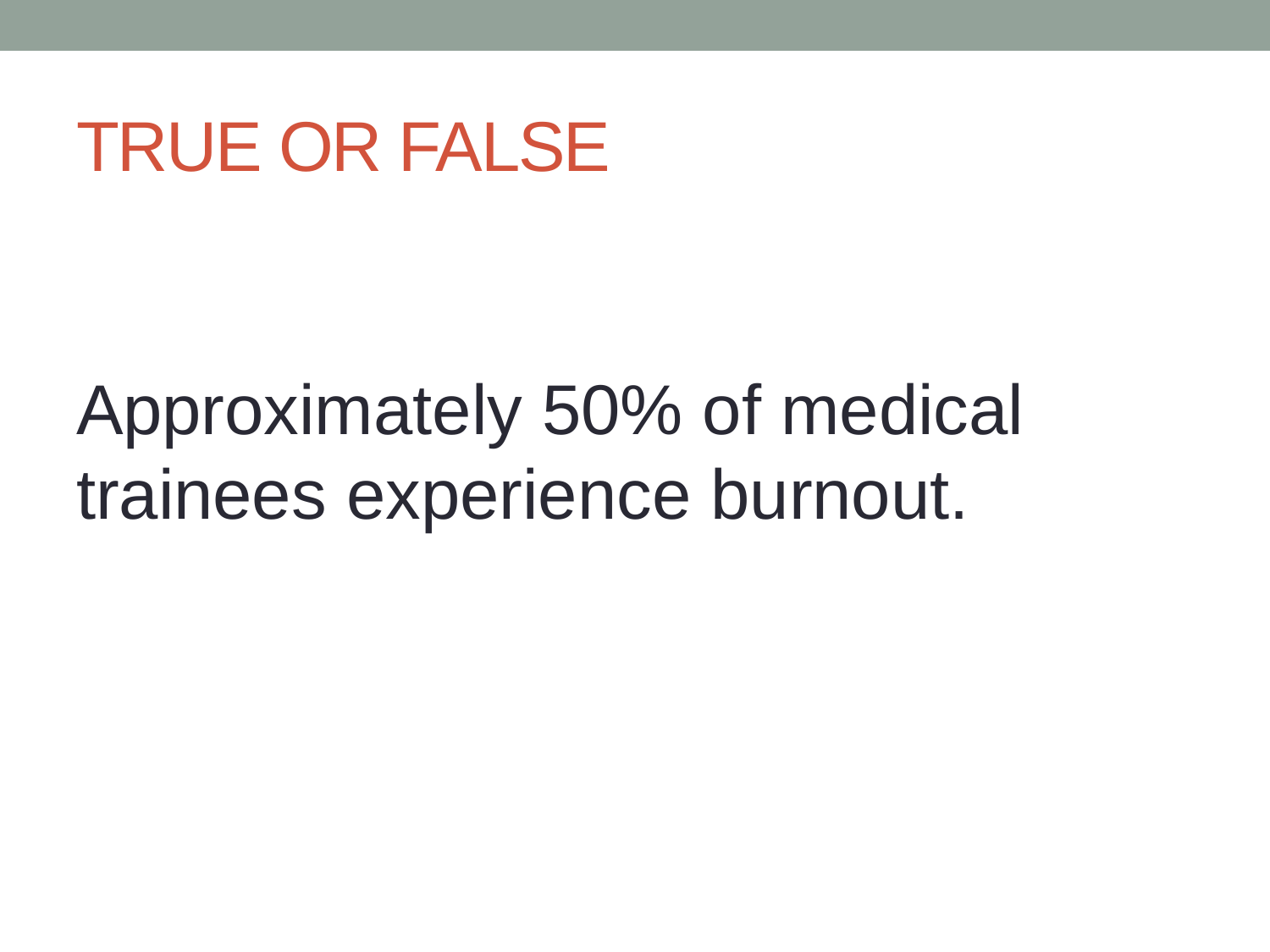

# TRUE OR FALSE
Approximately 50% of medical trainees experience burnout.

## Slide 12
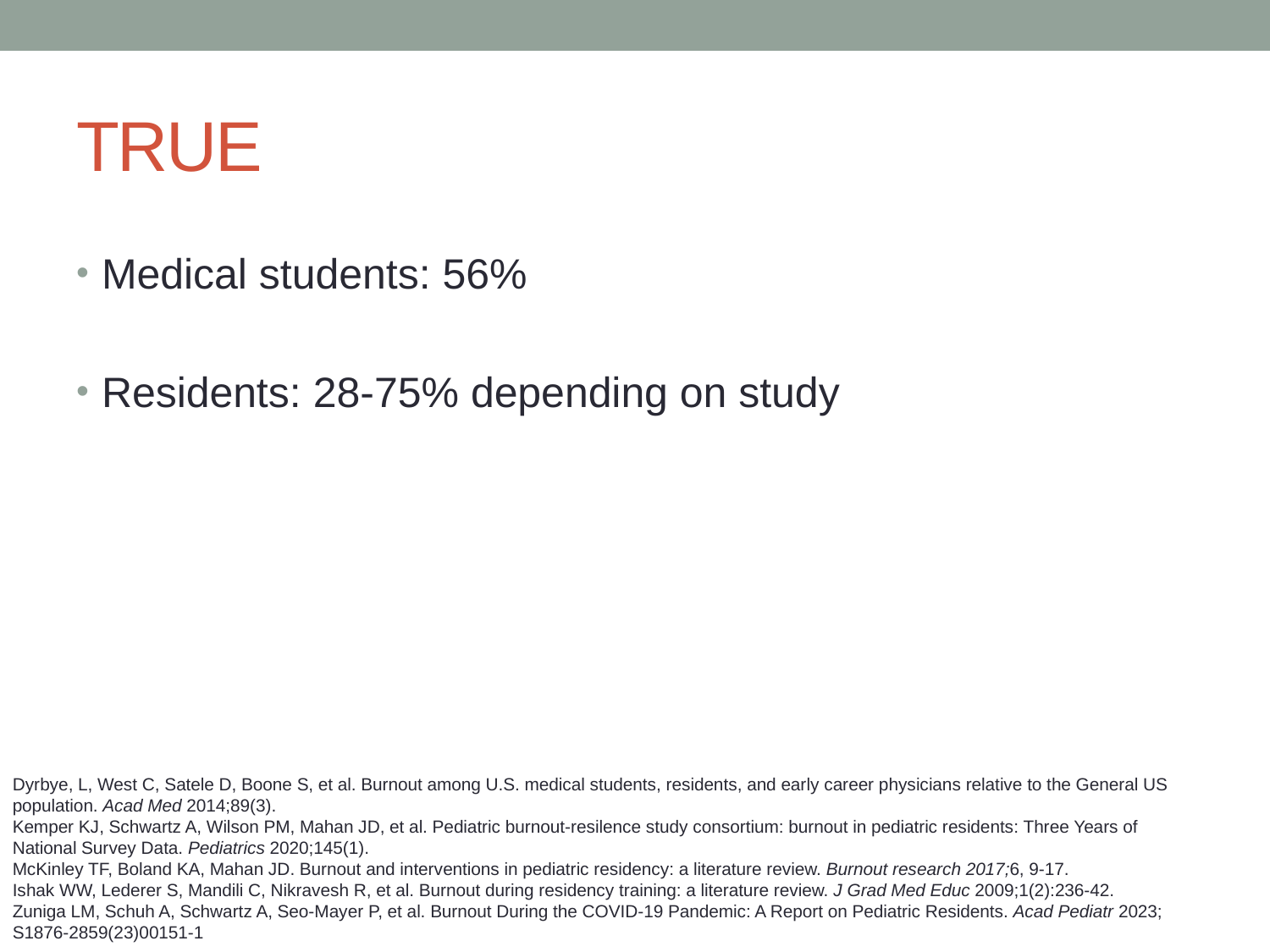

# TRUE
Medical students: 56%
Residents: 28-75% depending on study
Dyrbye, L, West C, Satele D, Boone S, et al. Burnout among U.S. medical students, residents, and early career physicians relative to the General US population. Acad Med 2014;89(3).
Kemper KJ, Schwartz A, Wilson PM, Mahan JD, et al. Pediatric burnout-resilence study consortium: burnout in pediatric residents: Three Years of National Survey Data. Pediatrics 2020;145(1).
McKinley TF, Boland KA, Mahan JD. Burnout and interventions in pediatric residency: a literature review. Burnout research 2017;6, 9-17.
Ishak WW, Lederer S, Mandili C, Nikravesh R, et al. Burnout during residency training: a literature review. J Grad Med Educ 2009;1(2):236-42.
Zuniga LM, Schuh A, Schwartz A, Seo-Mayer P, et al. Burnout During the COVID-19 Pandemic: A Report on Pediatric Residents. Acad Pediatr 2023; S1876-2859(23)00151-1

## Slide 13
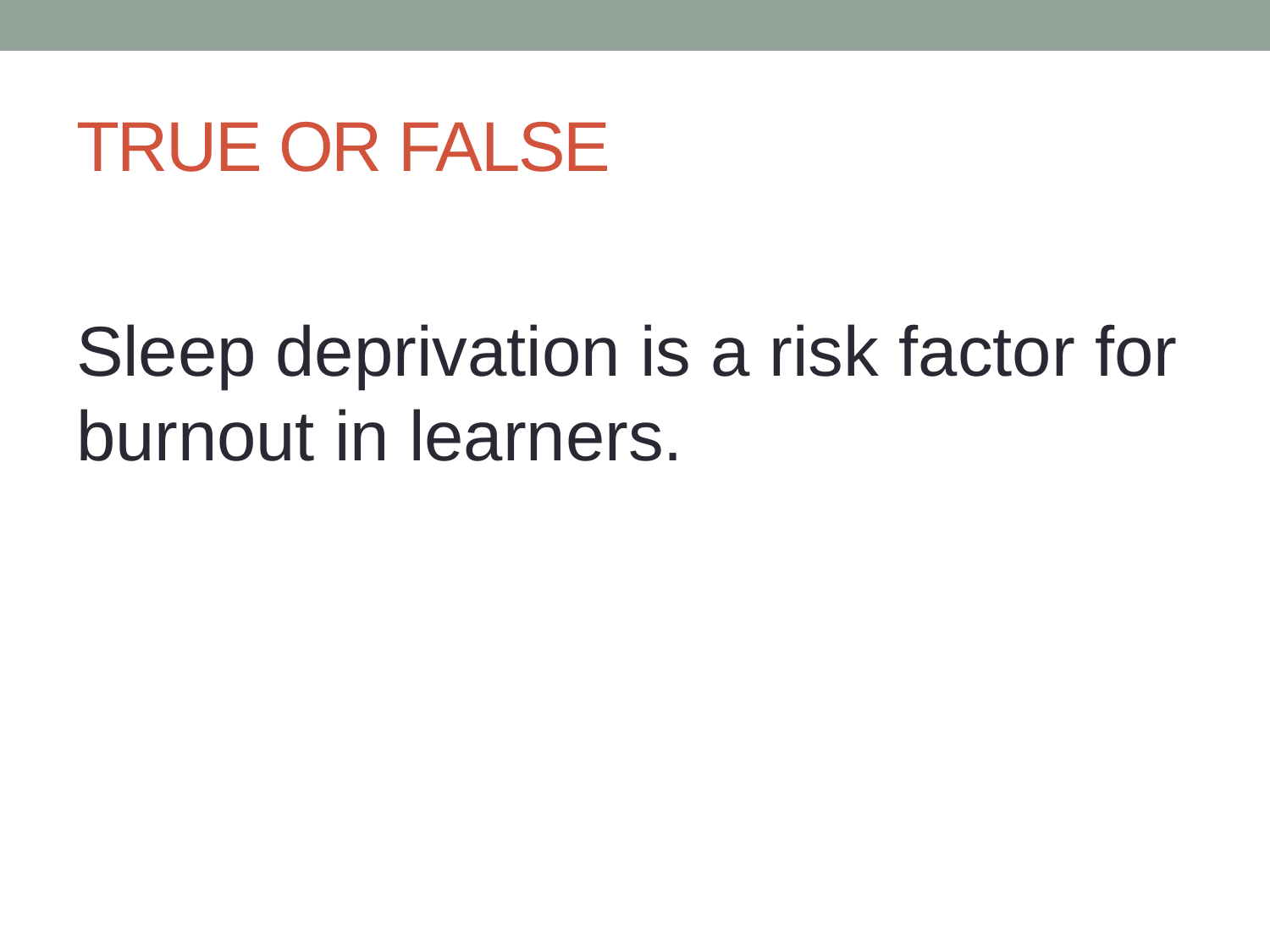

# TRUE OR FALSE
Sleep deprivation is a risk factor for burnout in learners.

## Slide 14
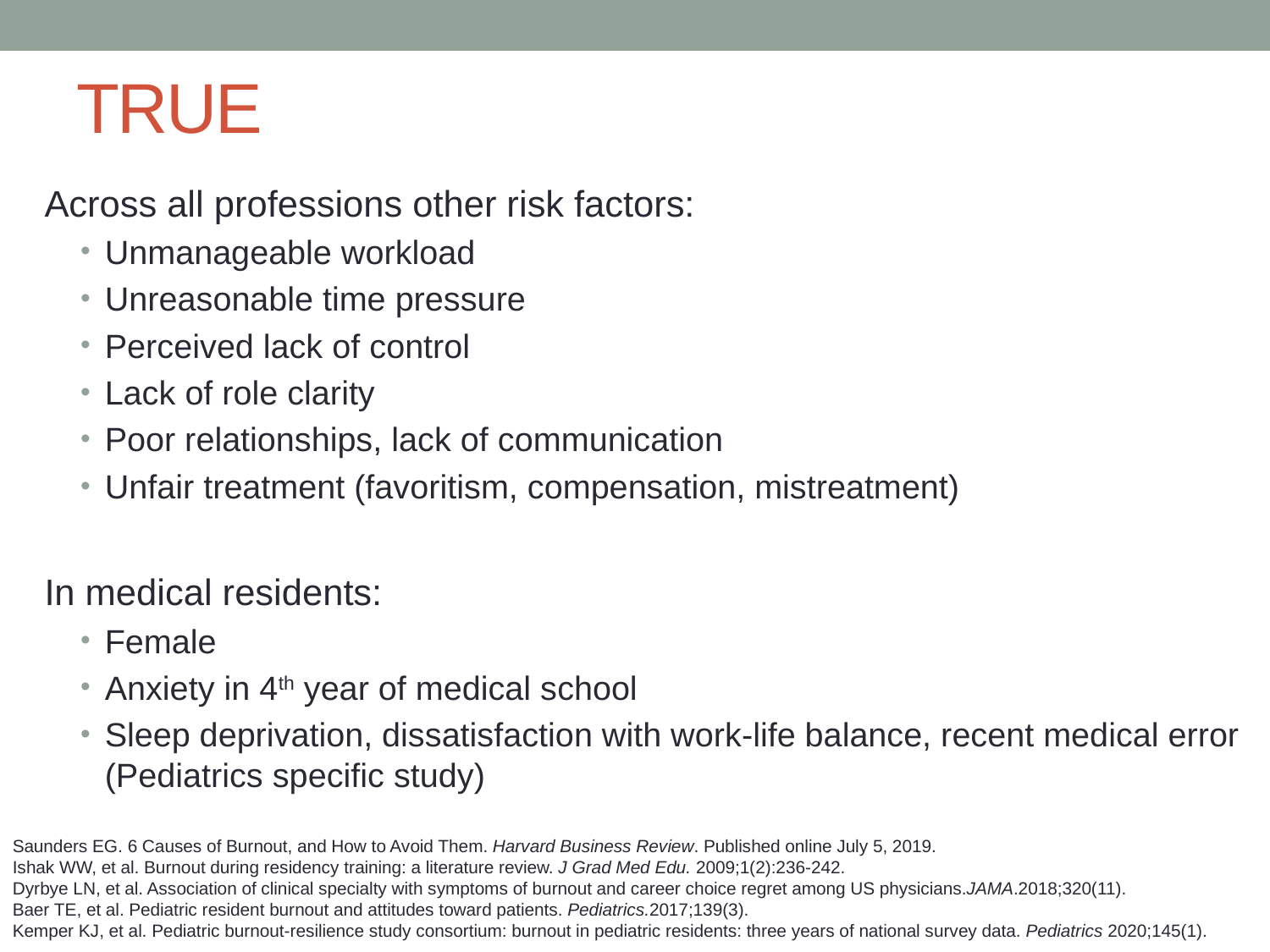

# TRUE
Across all professions other risk factors:
Unmanageable workload
Unreasonable time pressure
Perceived lack of control
Lack of role clarity
Poor relationships, lack of communication
Unfair treatment (favoritism, compensation, mistreatment)
In medical residents:
Female
Anxiety in 4th year of medical school
Sleep deprivation, dissatisfaction with work-life balance, recent medical error (Pediatrics specific study)
Saunders EG. 6 Causes of Burnout, and How to Avoid Them. Harvard Business Review. Published online July 5, 2019.
Ishak WW, et al. Burnout during residency training: a literature review. J Grad Med Edu. 2009;1(2):236-242.
Dyrbye LN, et al. Association of clinical specialty with symptoms of burnout and career choice regret among US physicians.JAMA.2018;320(11).
Baer TE, et al. Pediatric resident burnout and attitudes toward patients. Pediatrics.2017;139(3).
Kemper KJ, et al. Pediatric burnout-resilience study consortium: burnout in pediatric residents: three years of national survey data. Pediatrics 2020;145(1).

## Slide 15
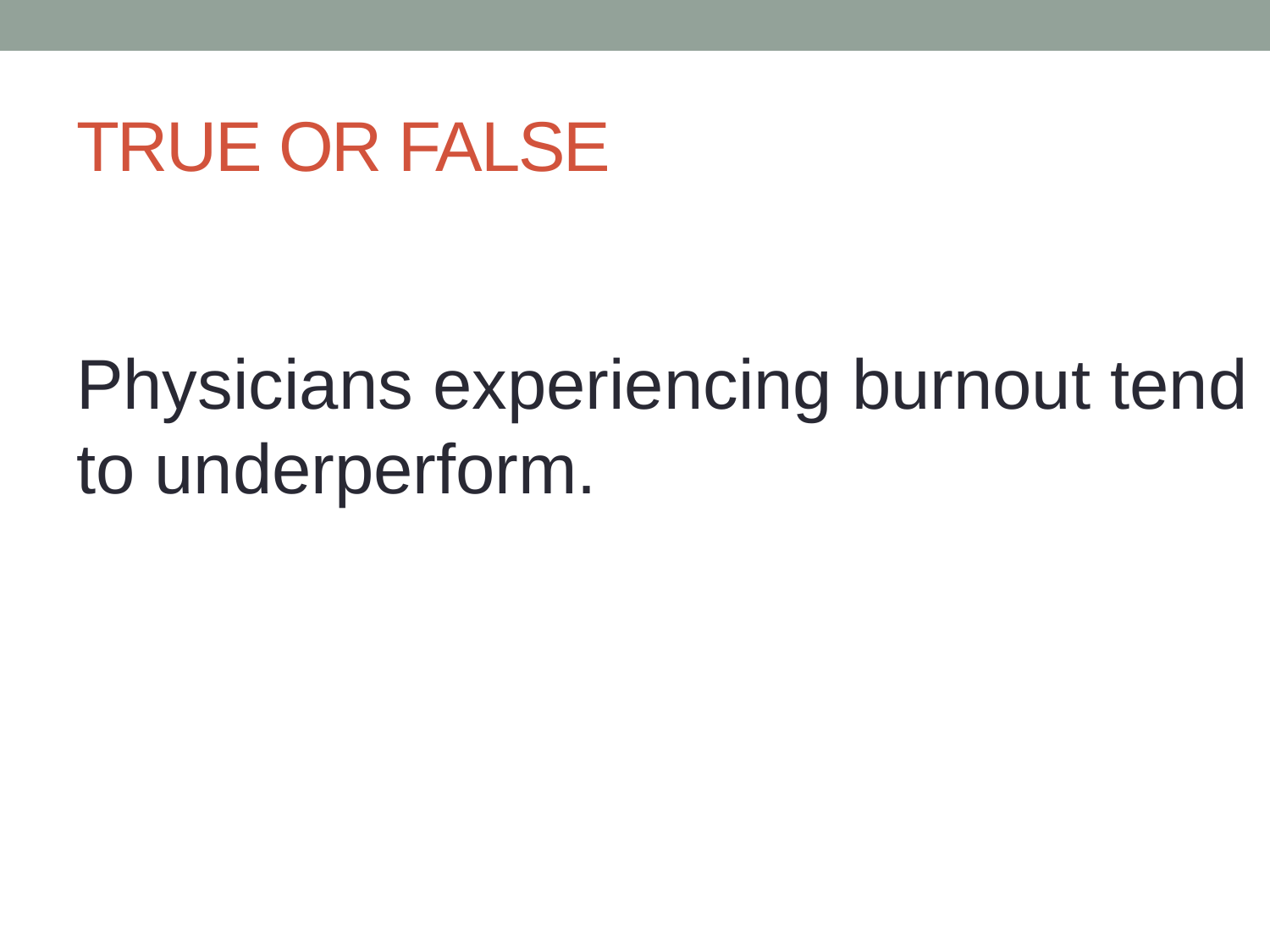

# TRUE OR FALSE
Physicians experiencing burnout tend to underperform.

## Slide 16
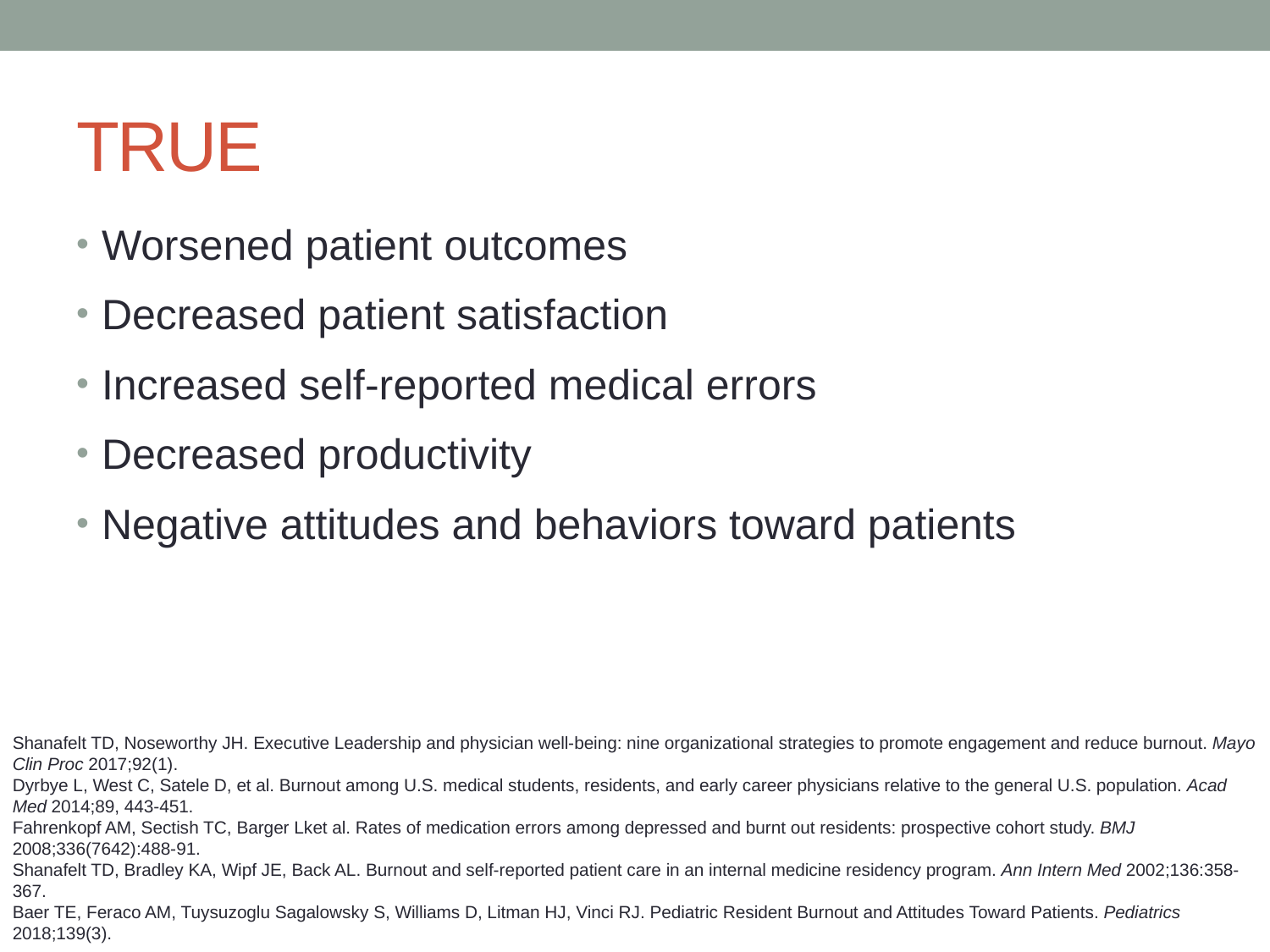

# TRUE
Worsened patient outcomes
Decreased patient satisfaction
Increased self-reported medical errors
Decreased productivity
Negative attitudes and behaviors toward patients
Shanafelt TD, Noseworthy JH. Executive Leadership and physician well-being: nine organizational strategies to promote engagement and reduce burnout. Mayo Clin Proc 2017;92(1).
Dyrbye L, West C, Satele D, et al. Burnout among U.S. medical students, residents, and early career physicians relative to the general U.S. population. Acad Med 2014;89, 443-451.
Fahrenkopf AM, Sectish TC, Barger Lket al. Rates of medication errors among depressed and burnt out residents: prospective cohort study. BMJ 2008;336(7642):488-91.
Shanafelt TD, Bradley KA, Wipf JE, Back AL. Burnout and self-reported patient care in an internal medicine residency program. Ann Intern Med 2002;136:358-367.
Baer TE, Feraco AM, Tuysuzoglu Sagalowsky S, Williams D, Litman HJ, Vinci RJ. Pediatric Resident Burnout and Attitudes Toward Patients. Pediatrics 2018;139(3).

## Slide 17
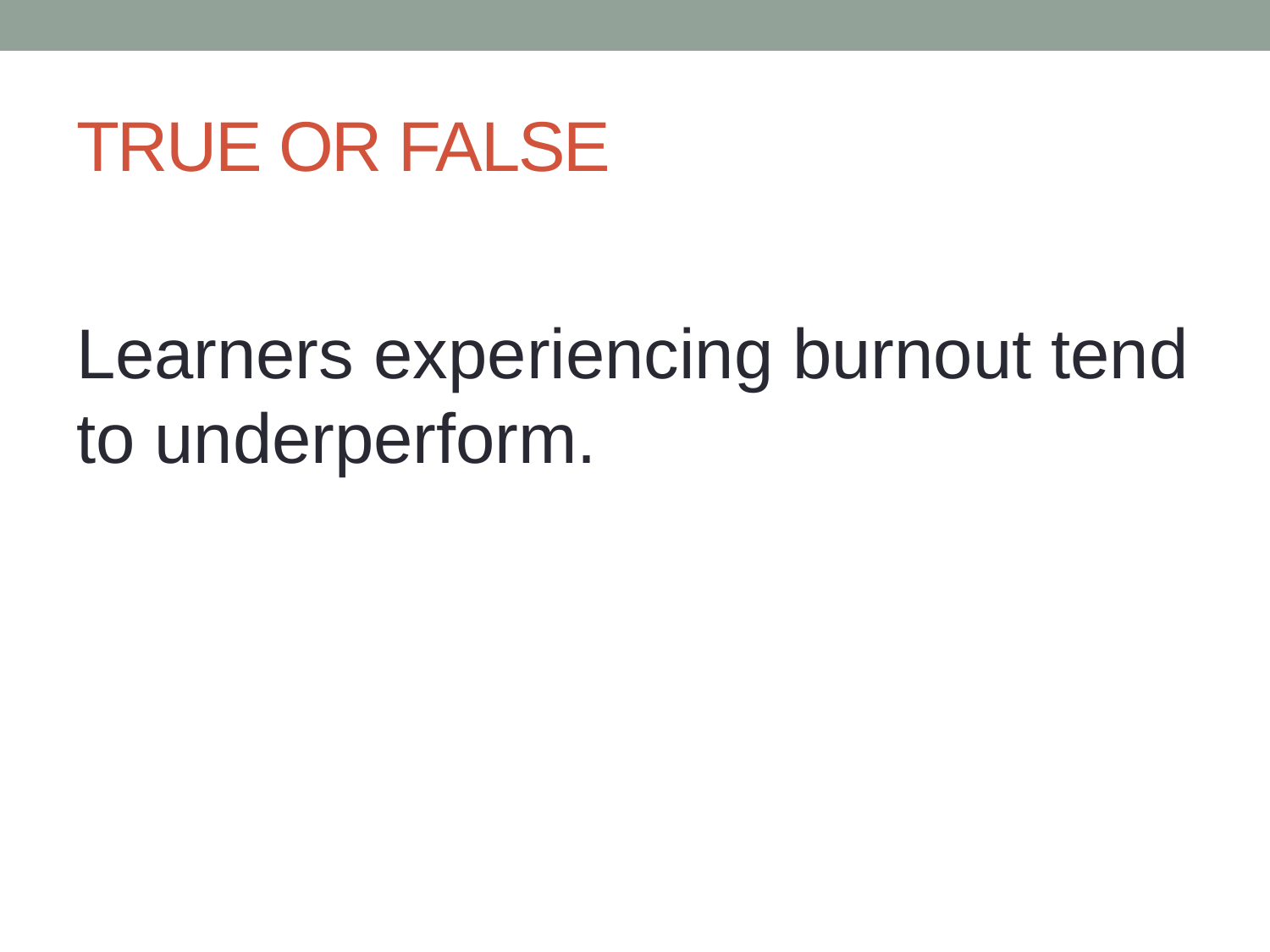

# TRUE OR FALSE
Learners experiencing burnout tend to underperform.

## Slide 18
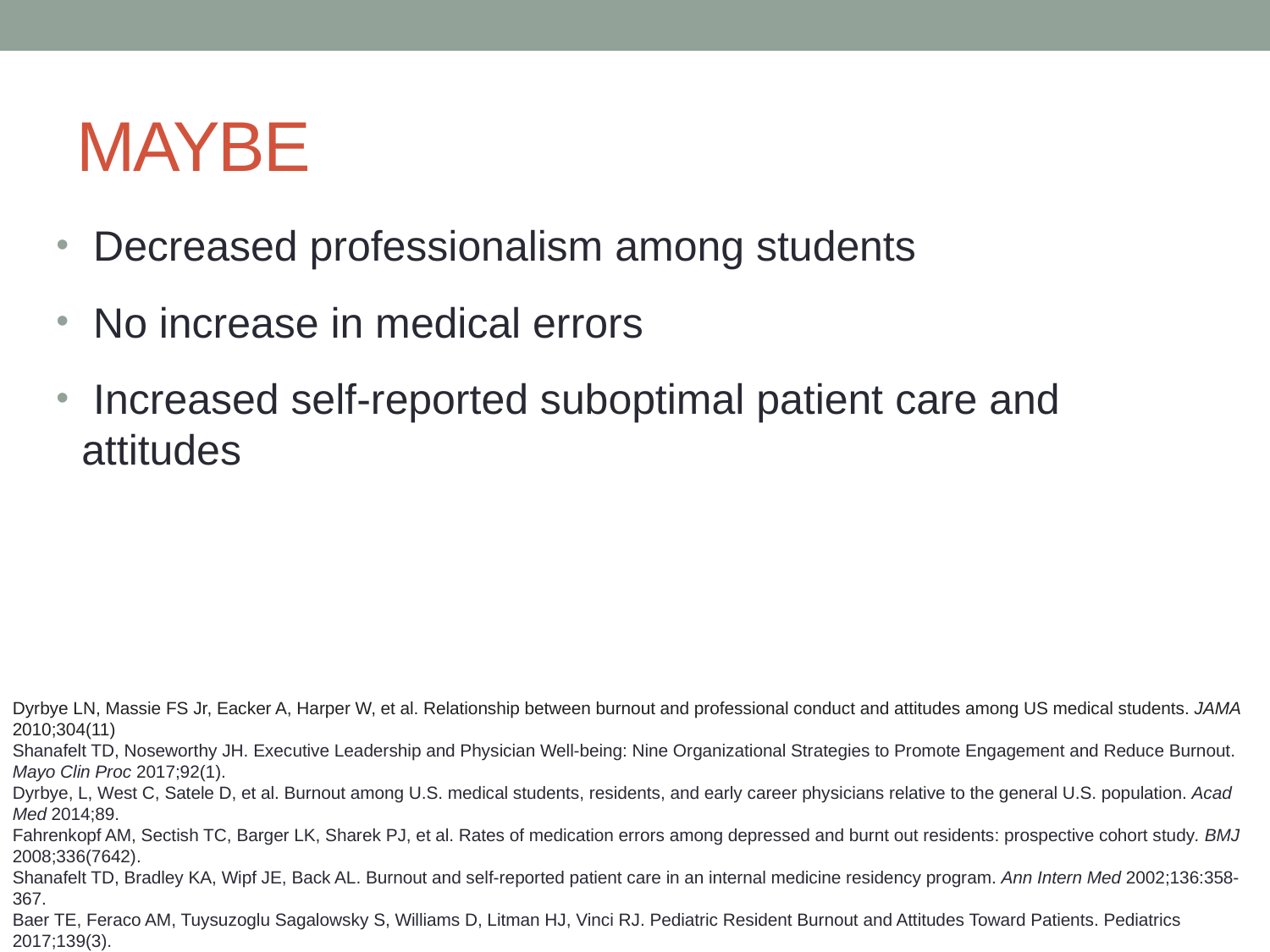

# MAYBE
 Decreased professionalism among students
 No increase in medical errors
 Increased self-reported suboptimal patient care and attitudes
Dyrbye LN, Massie FS Jr, Eacker A, Harper W, et al. Relationship between burnout and professional conduct and attitudes among US medical students. JAMA 2010;304(11)
Shanafelt TD, Noseworthy JH. Executive Leadership and Physician Well-being: Nine Organizational Strategies to Promote Engagement and Reduce Burnout. Mayo Clin Proc 2017;92(1).
Dyrbye, L, West C, Satele D, et al. Burnout among U.S. medical students, residents, and early career physicians relative to the general U.S. population. Acad Med 2014;89.
Fahrenkopf AM, Sectish TC, Barger LK, Sharek PJ, et al. Rates of medication errors among depressed and burnt out residents: prospective cohort study. BMJ 2008;336(7642).
Shanafelt TD, Bradley KA, Wipf JE, Back AL. Burnout and self-reported patient care in an internal medicine residency program. Ann Intern Med 2002;136:358-367.
Baer TE, Feraco AM, Tuysuzoglu Sagalowsky S, Williams D, Litman HJ, Vinci RJ. Pediatric Resident Burnout and Attitudes Toward Patients. Pediatrics 2017;139(3).

## Slide 19
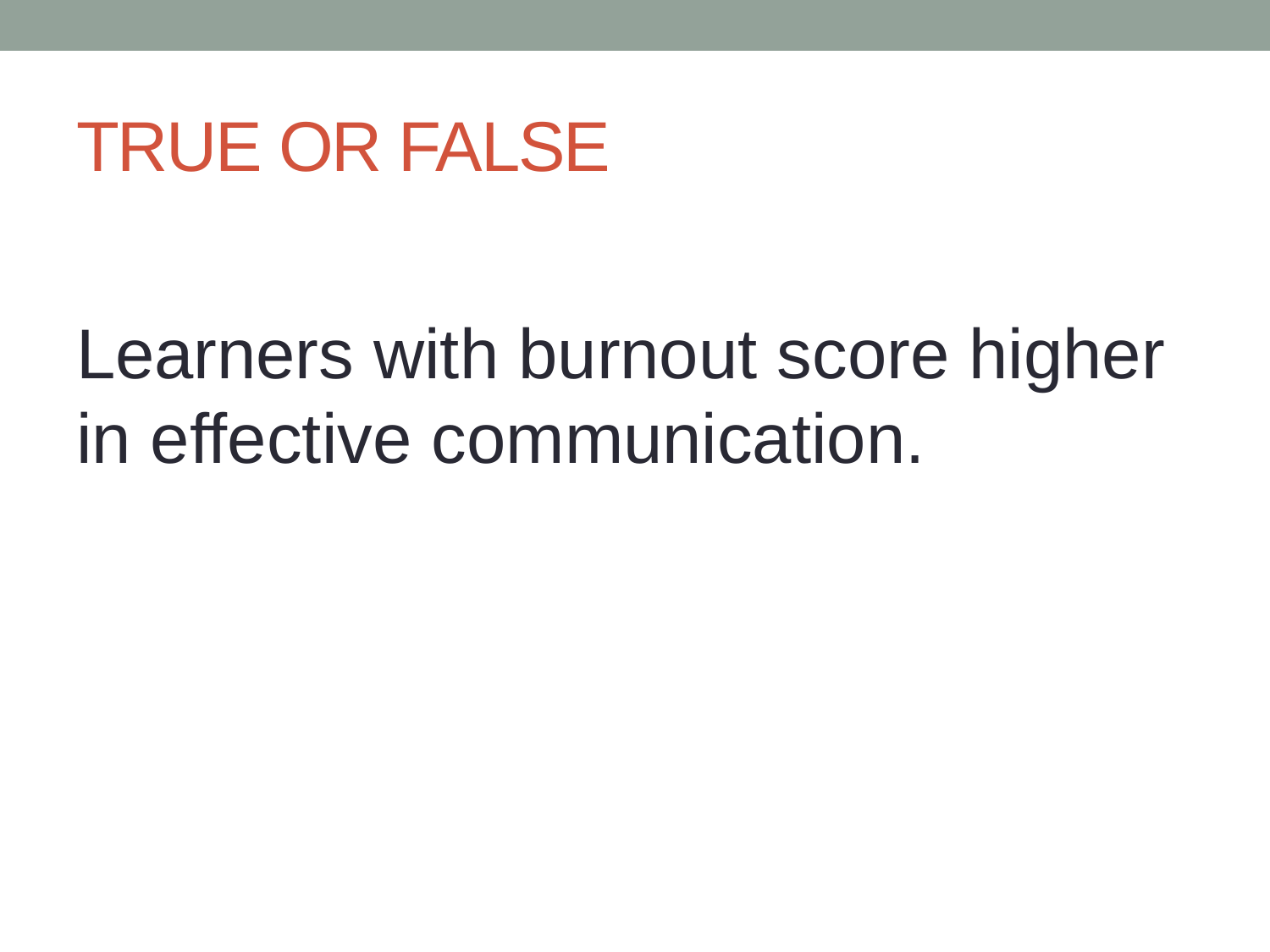

# TRUE OR FALSE
Learners with burnout score higher in effective communication.

## Slide 20
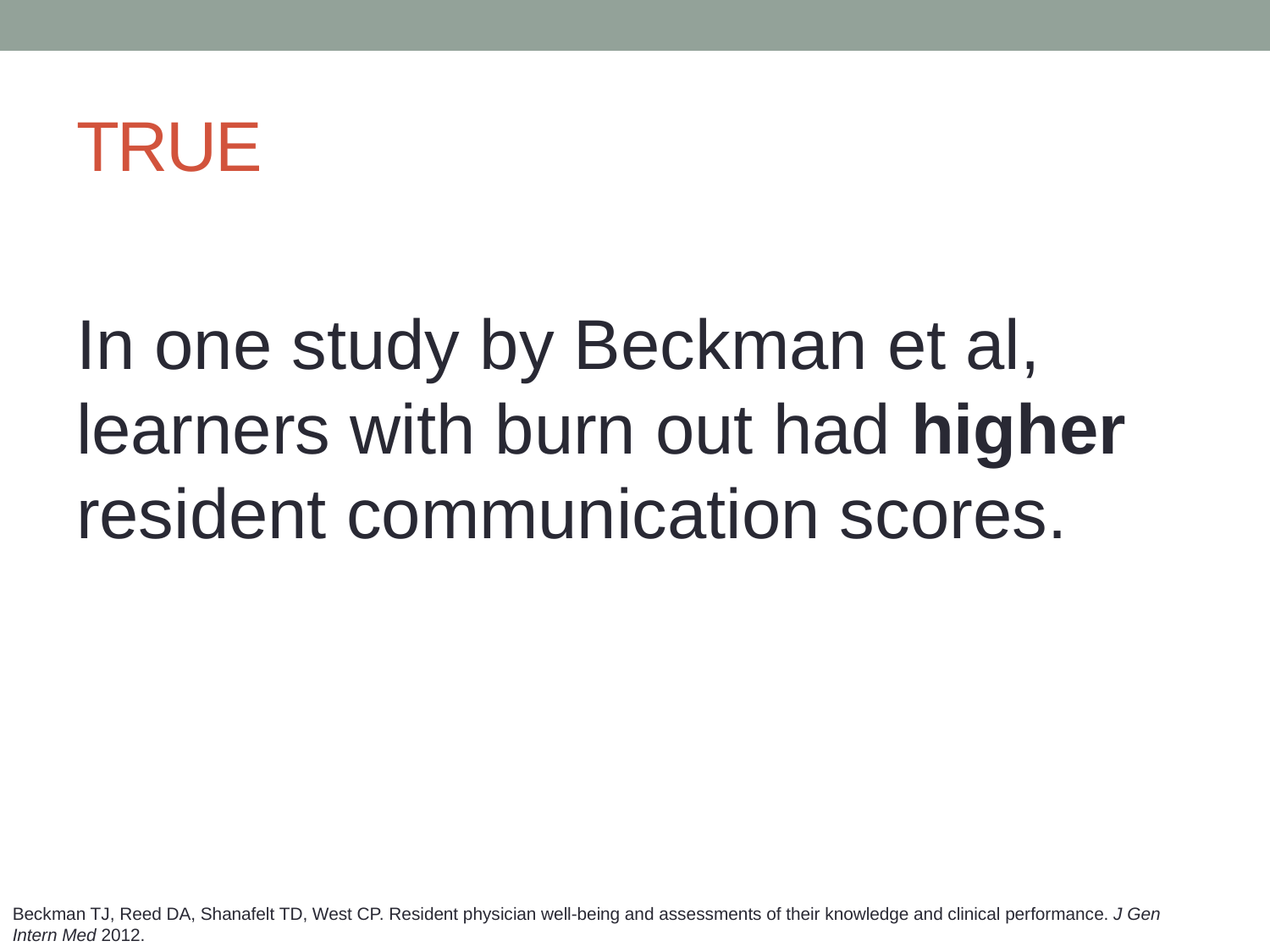

# TRUE
In one study by Beckman et al, learners with burn out had higher resident communication scores.
.
Beckman TJ, Reed DA, Shanafelt TD, West CP. Resident physician well-being and assessments of their knowledge and clinical performance. J Gen Intern Med 2012.

## Slide 21
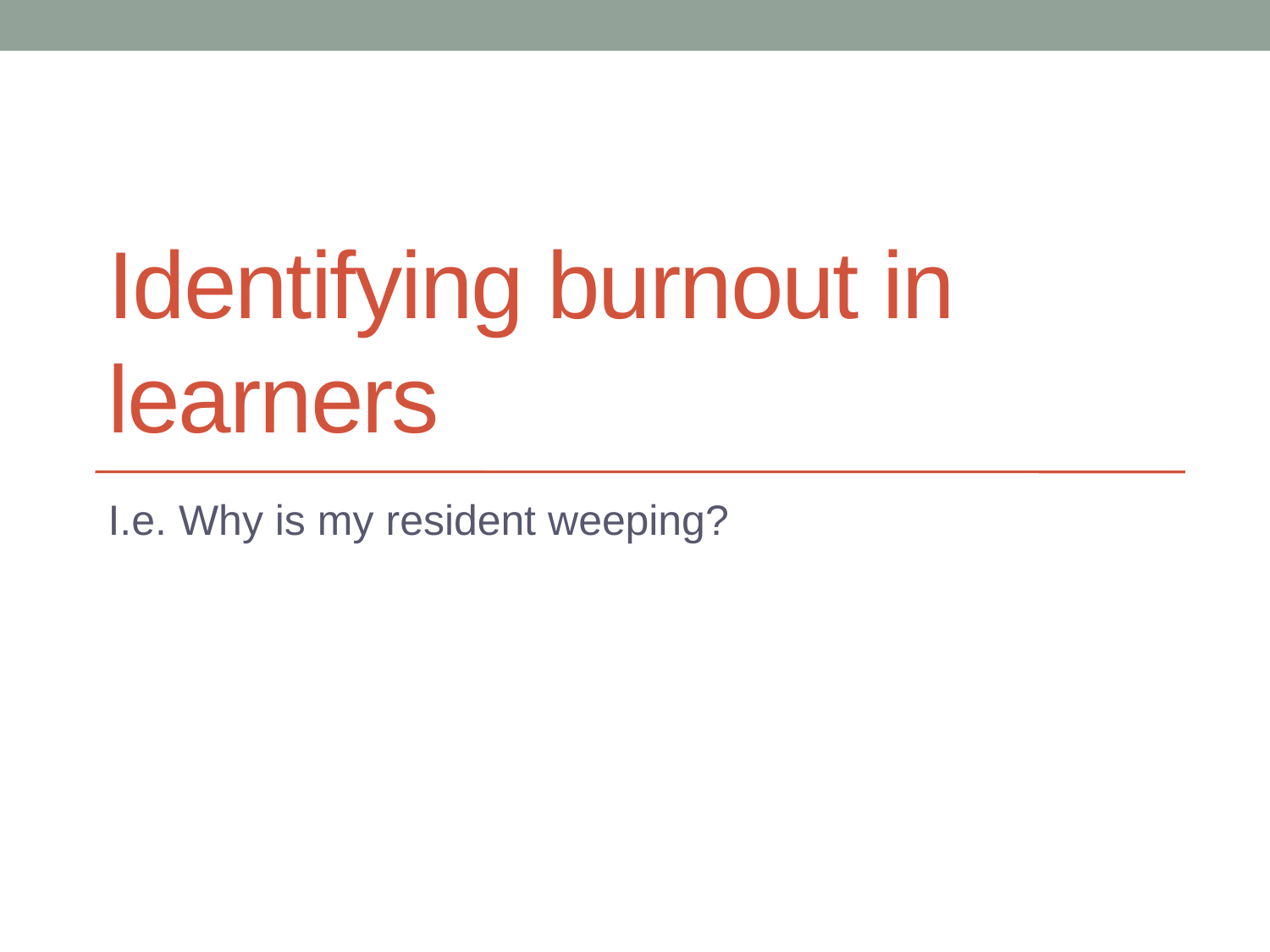

# Identifying burnout in learners
I.e. Why is my resident weeping?

## Slide 22
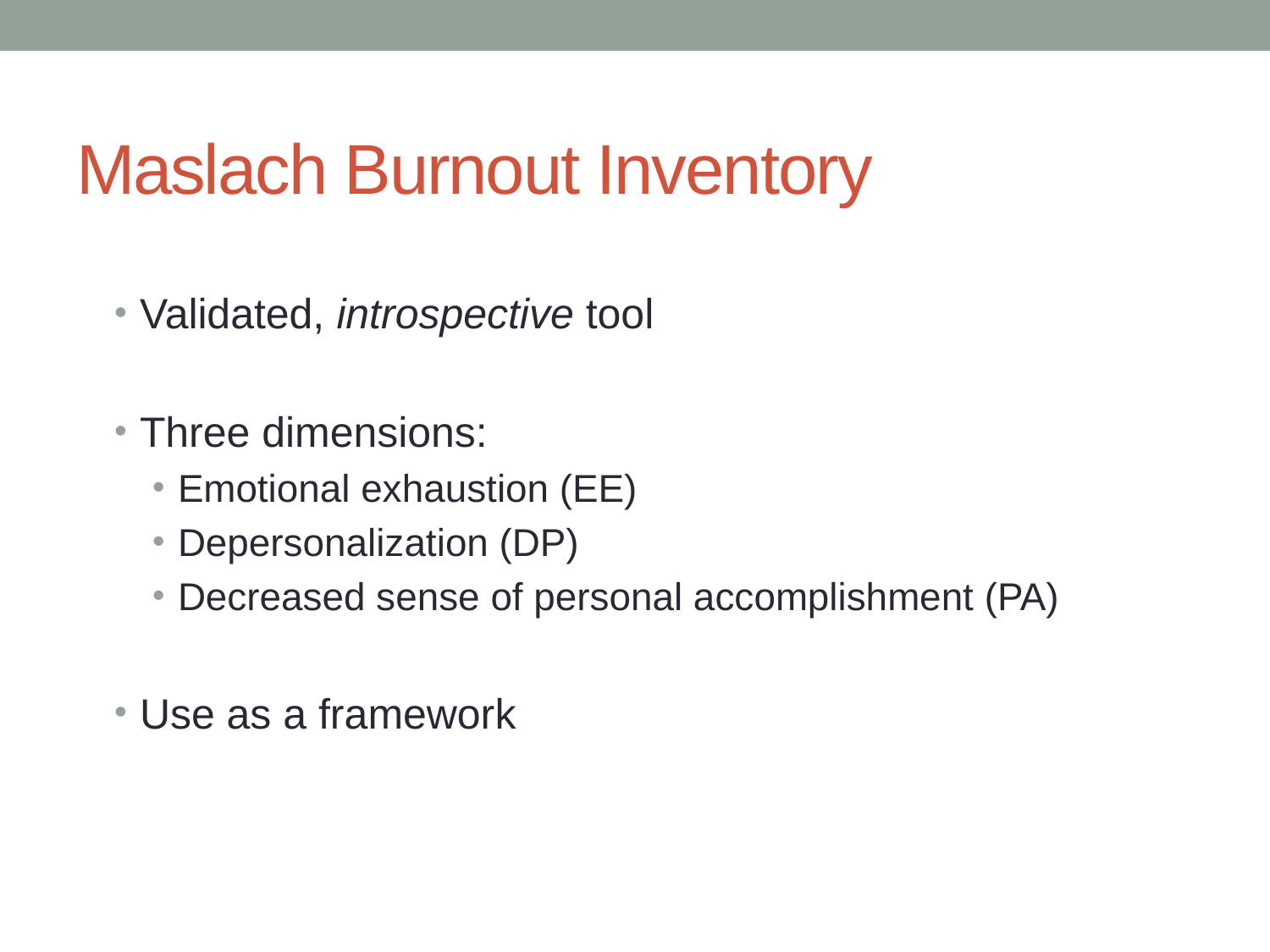

# Maslach Burnout Inventory
Validated, introspective tool
Three dimensions:
Emotional exhaustion (EE)
Depersonalization (DP)
Decreased sense of personal accomplishment (PA)
Use as a framework

## Slide 23
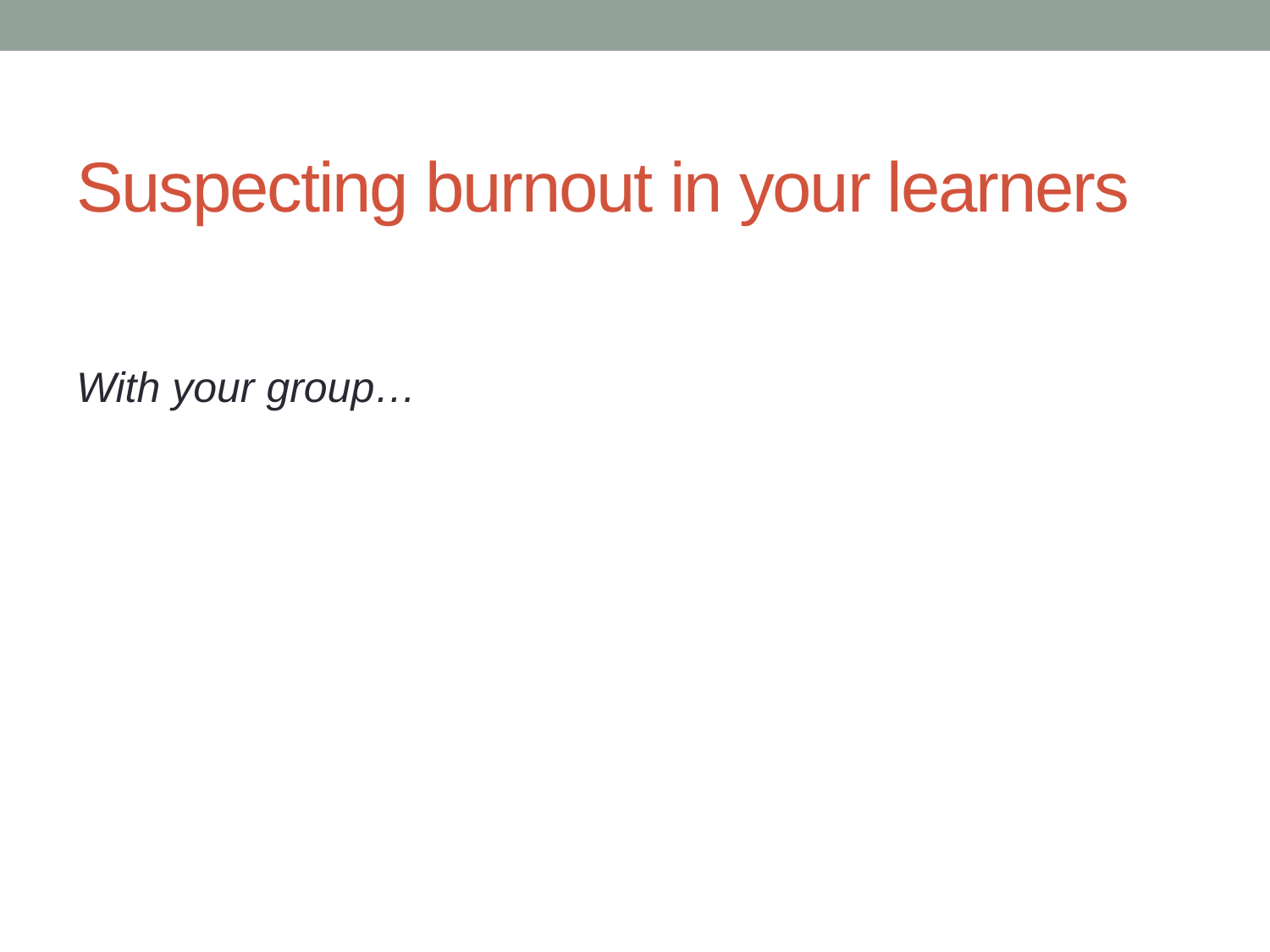

# Suspecting burnout in your learners
With your group…

## Slide 24
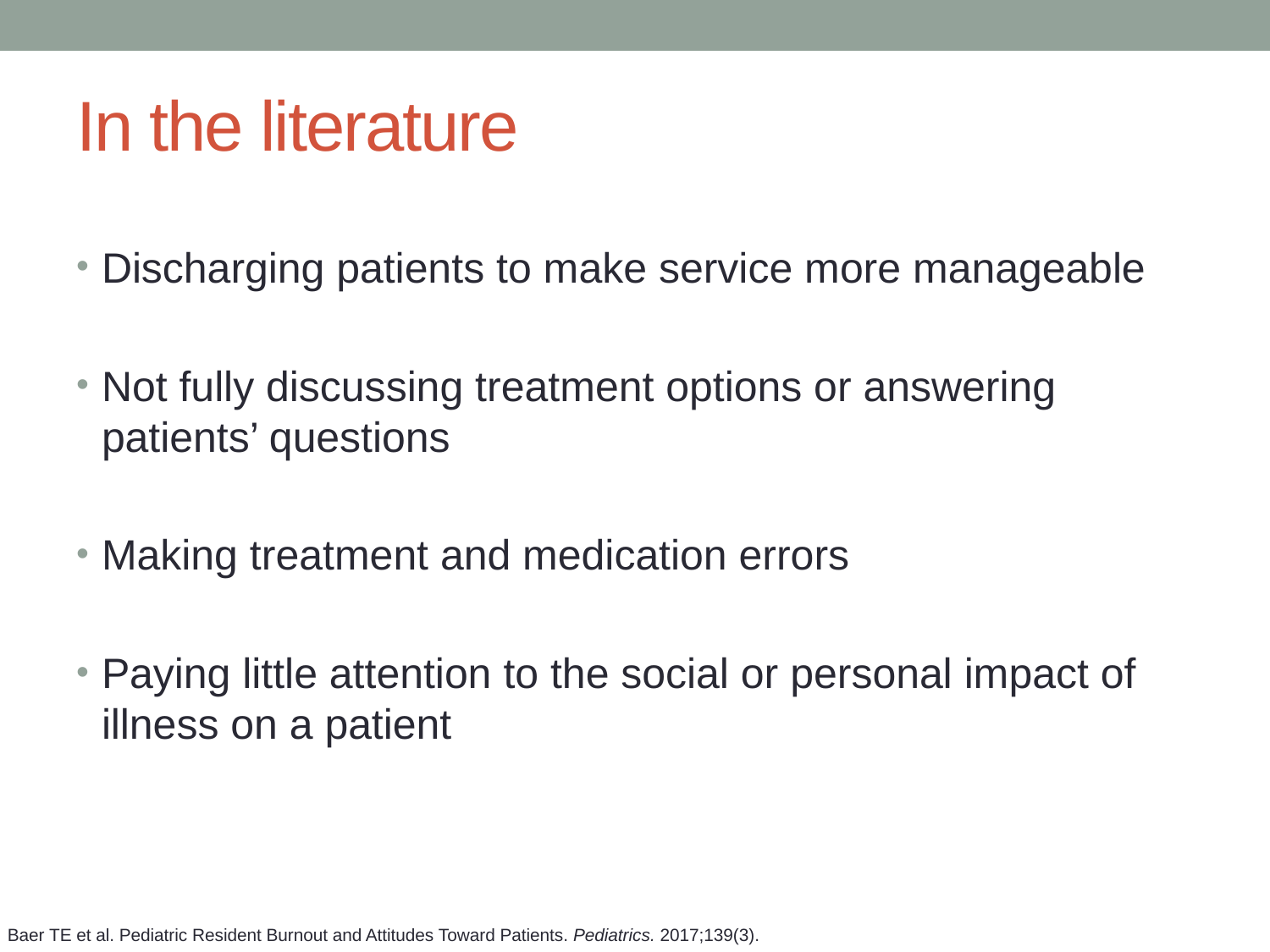

# In the literature
Discharging patients to make service more manageable
Not fully discussing treatment options or answering patients’ questions
Making treatment and medication errors
Paying little attention to the social or personal impact of illness on a patient
Baer TE et al. Pediatric Resident Burnout and Attitudes Toward Patients. Pediatrics. 2017;139(3).

## Slide 25
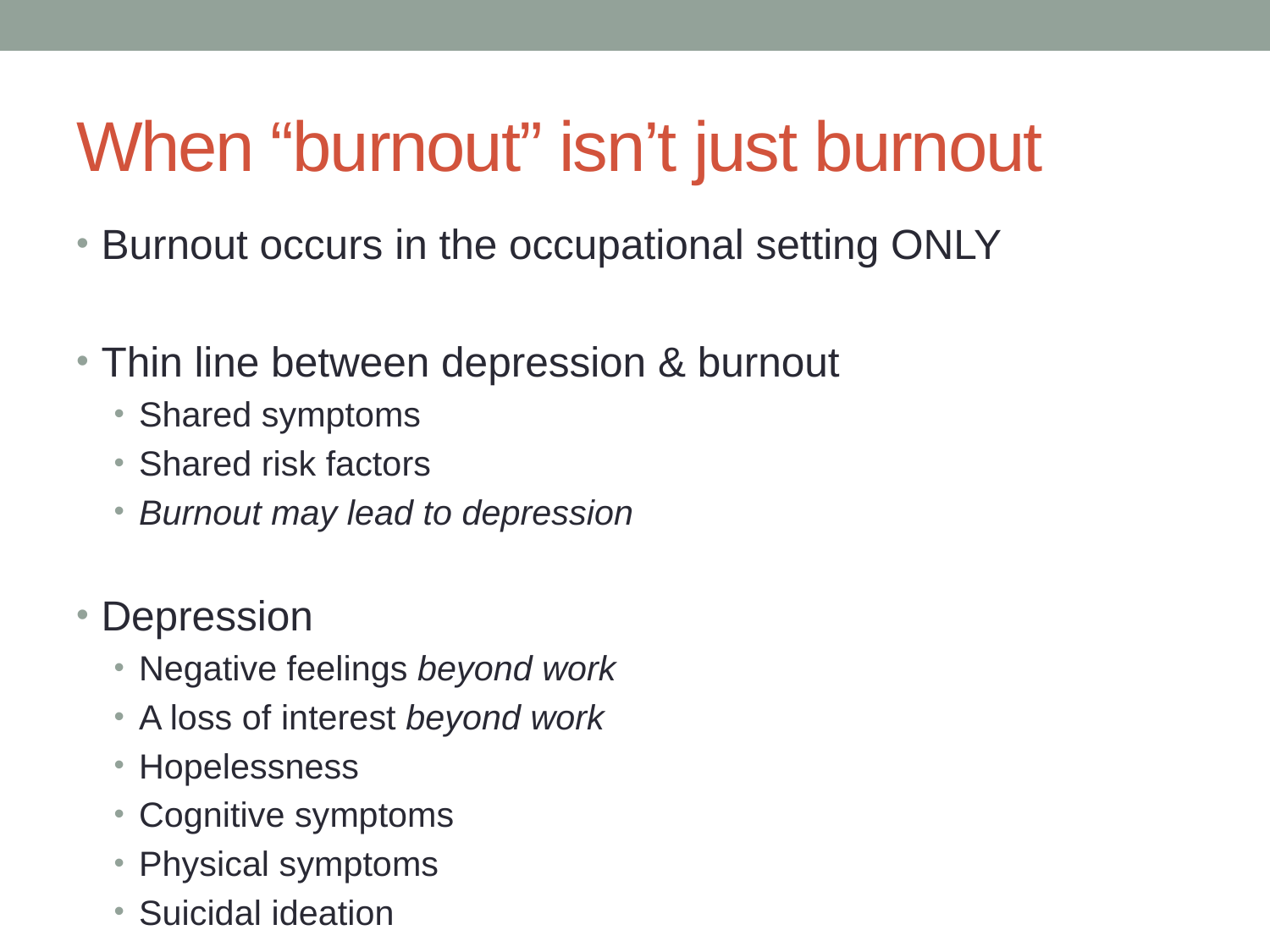

# When “burnout” isn’t just burnout
Burnout occurs in the occupational setting ONLY
Thin line between depression & burnout
Shared symptoms
Shared risk factors
Burnout may lead to depression
Depression
Negative feelings beyond work
A loss of interest beyond work
Hopelessness
Cognitive symptoms
Physical symptoms
Suicidal ideation

## Slide 26
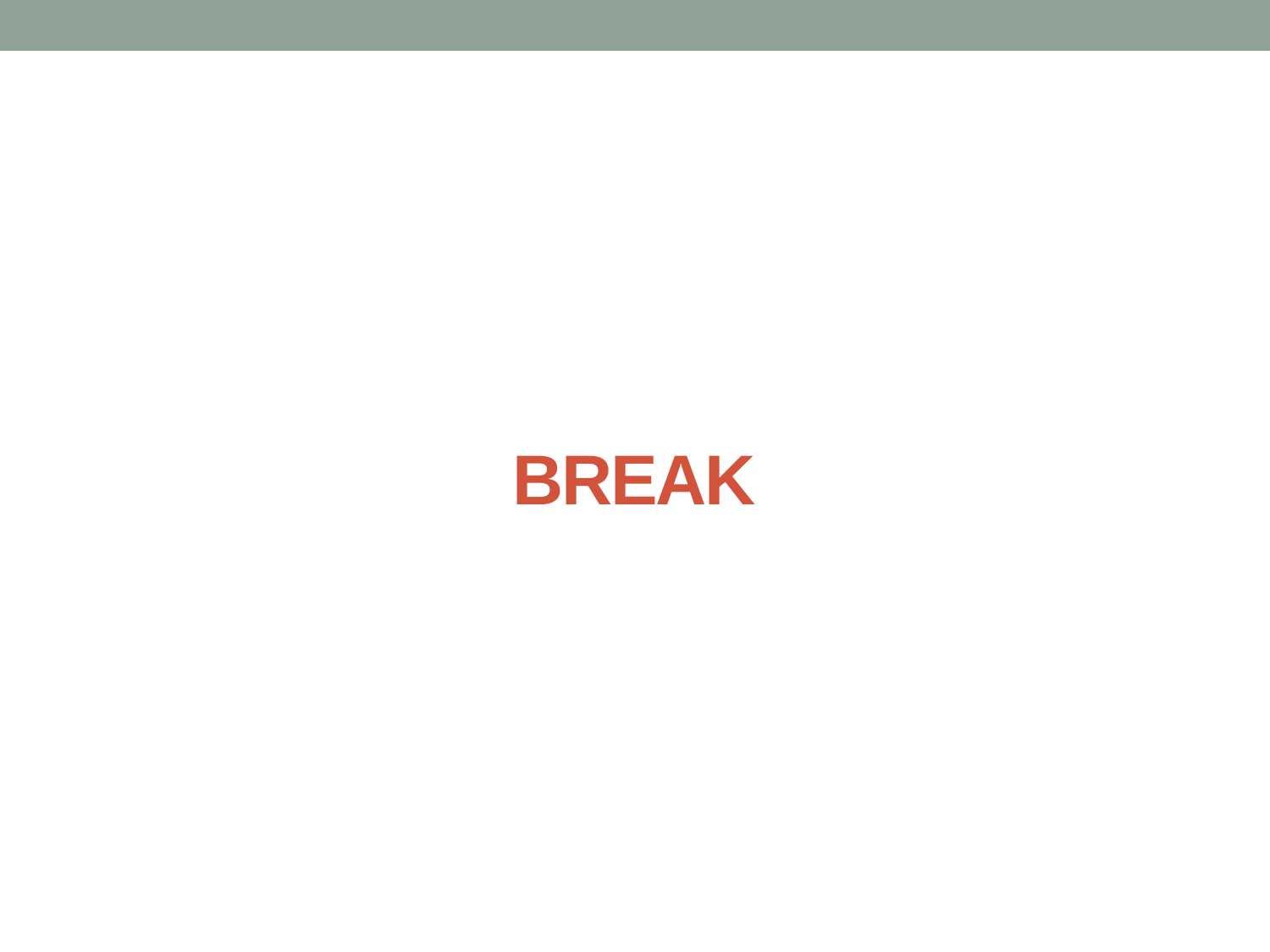

# BREAK

## Slide 27
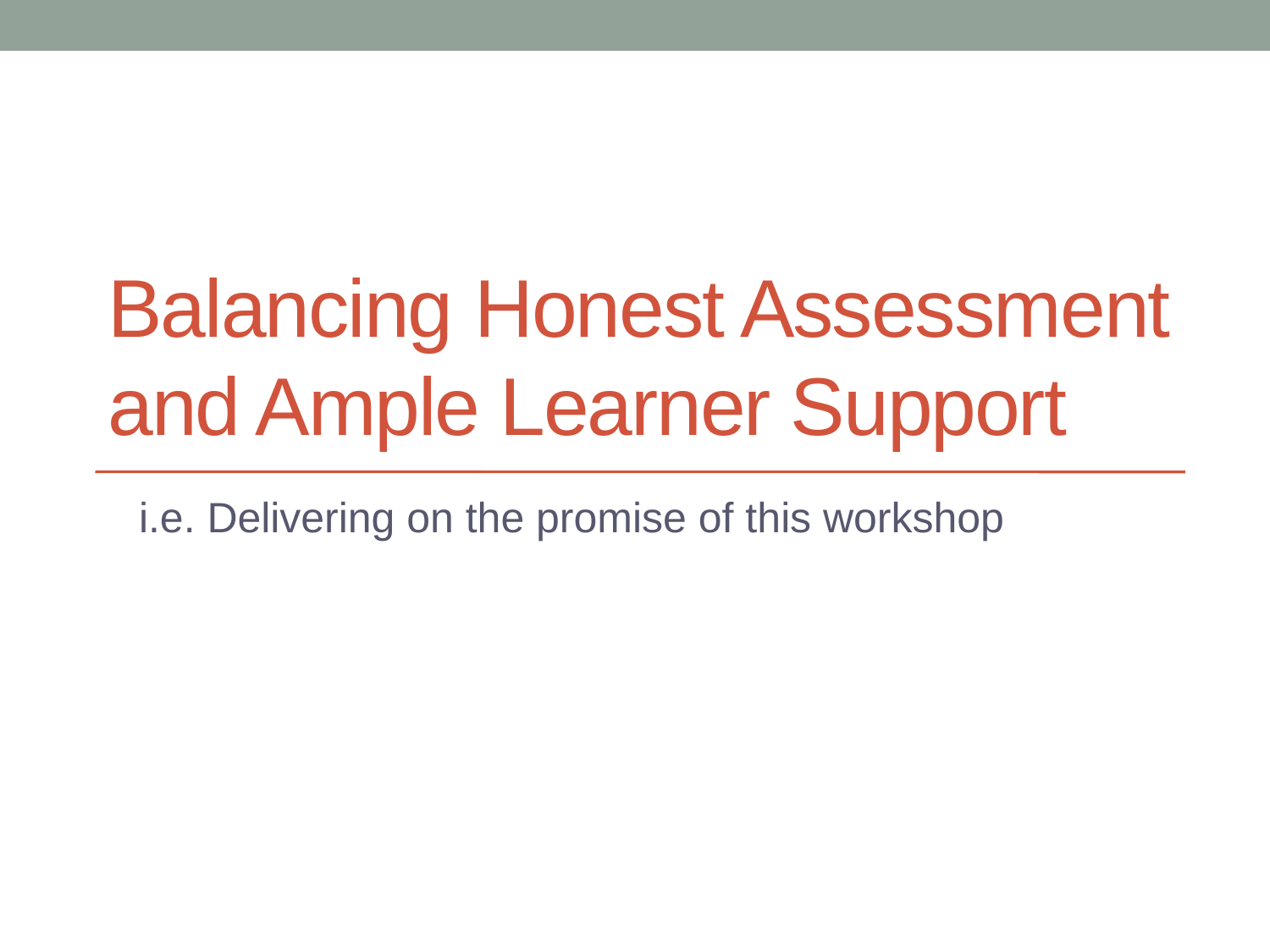

# Balancing Honest Assessment and Ample Learner Support
i.e. Delivering on the promise of this workshop

## Slide 28
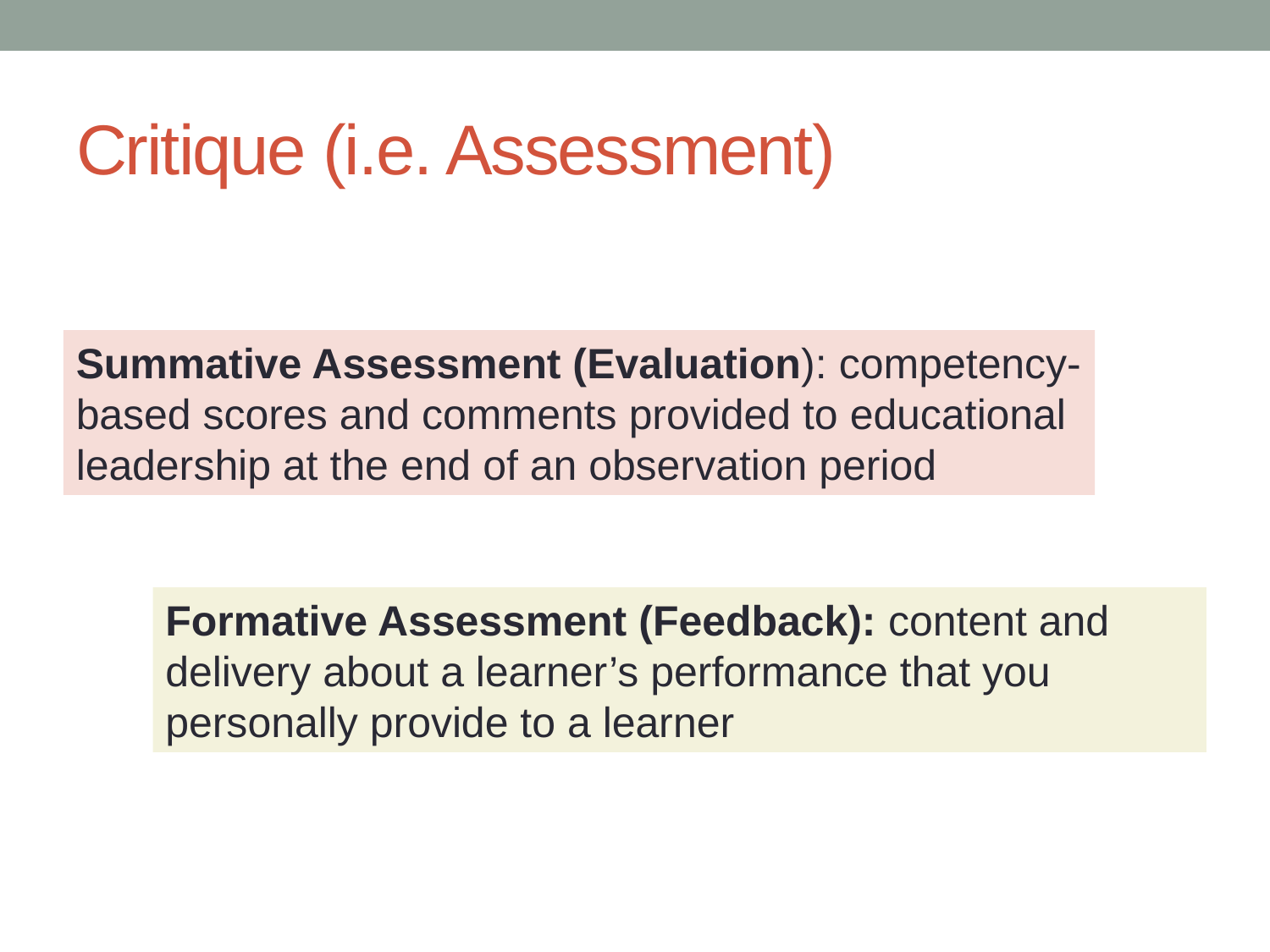

# Critique (i.e. Assessment)
Summative Assessment (Evaluation): competency-based scores and comments provided to educational leadership at the end of an observation period
Formative Assessment (Feedback): content and delivery about a learner’s performance that you personally provide to a learner

## Slide 29
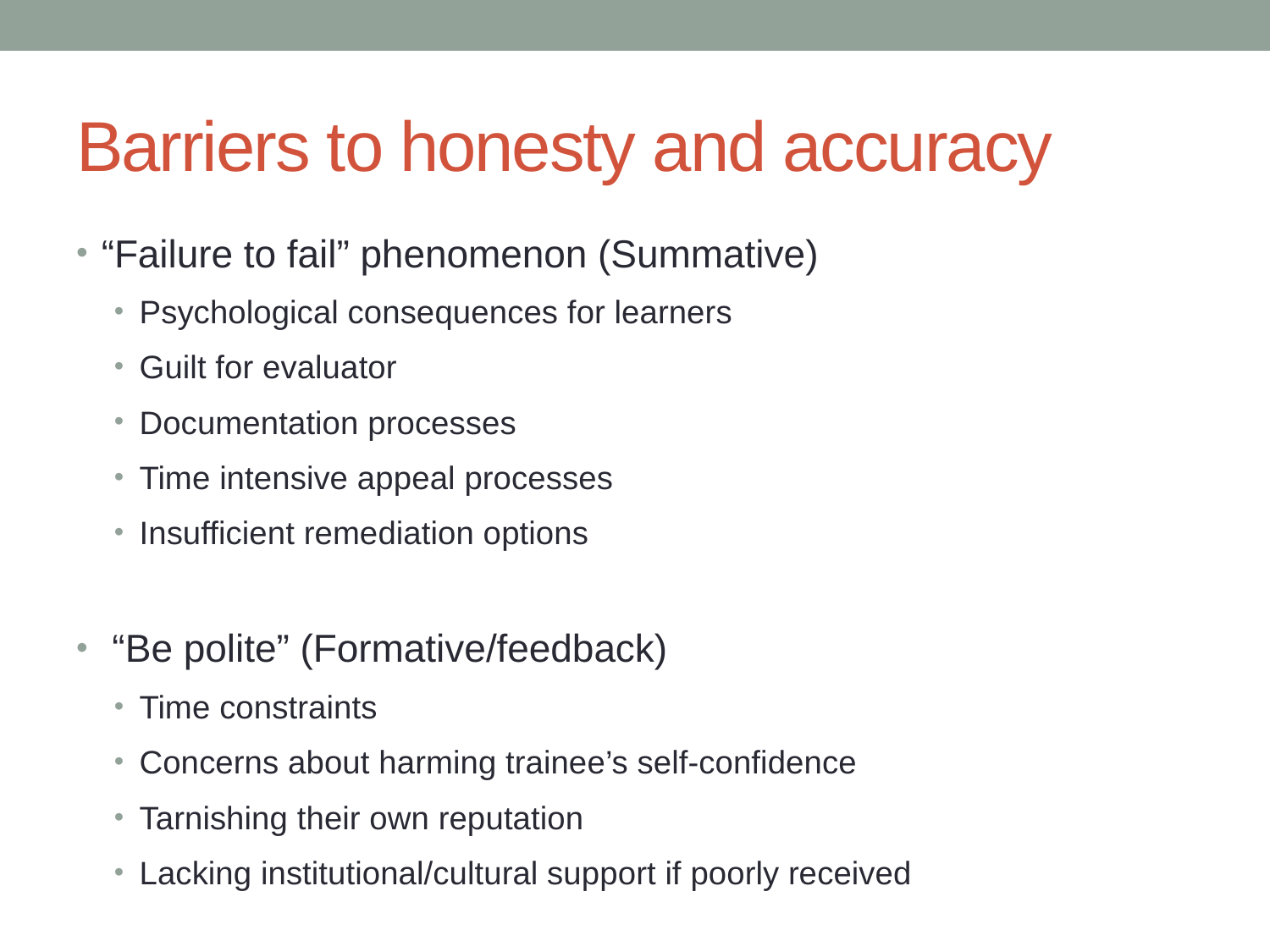

# Barriers to honesty and accuracy
“Failure to fail” phenomenon (Summative)
Psychological consequences for learners
Guilt for evaluator
Documentation processes
Time intensive appeal processes
Insufficient remediation options
 “Be polite” (Formative/feedback)
Time constraints
Concerns about harming trainee’s self-confidence
Tarnishing their own reputation
Lacking institutional/cultural support if poorly received

## Slide 30
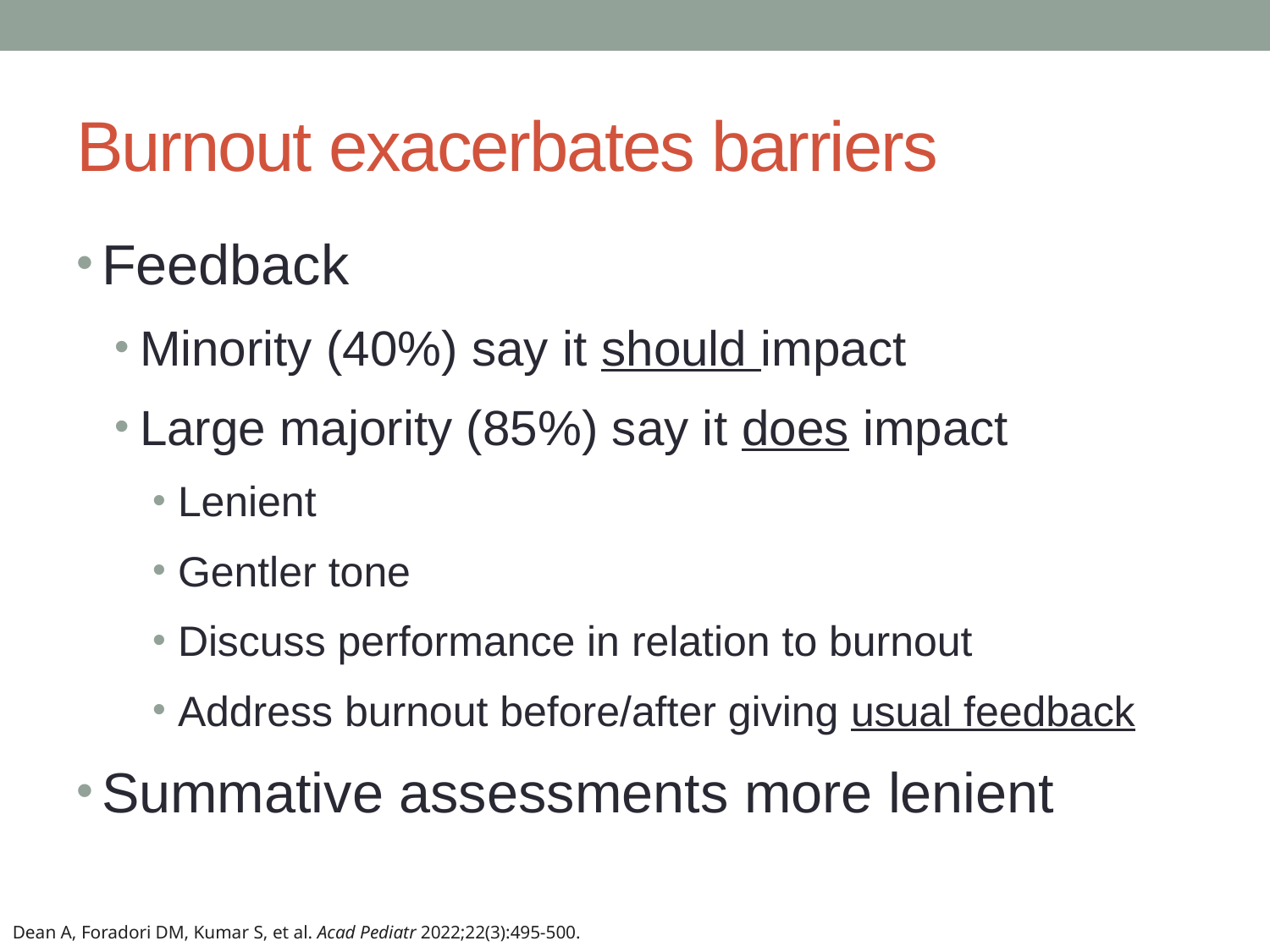

# Burnout exacerbates barriers
Feedback
Minority (40%) say it should impact
Large majority (85%) say it does impact
Lenient
Gentler tone
Discuss performance in relation to burnout
Address burnout before/after giving usual feedback
Summative assessments more lenient
Dean A, Foradori DM, Kumar S, et al. Acad Pediatr 2022;22(3):495-500.

## Slide 31
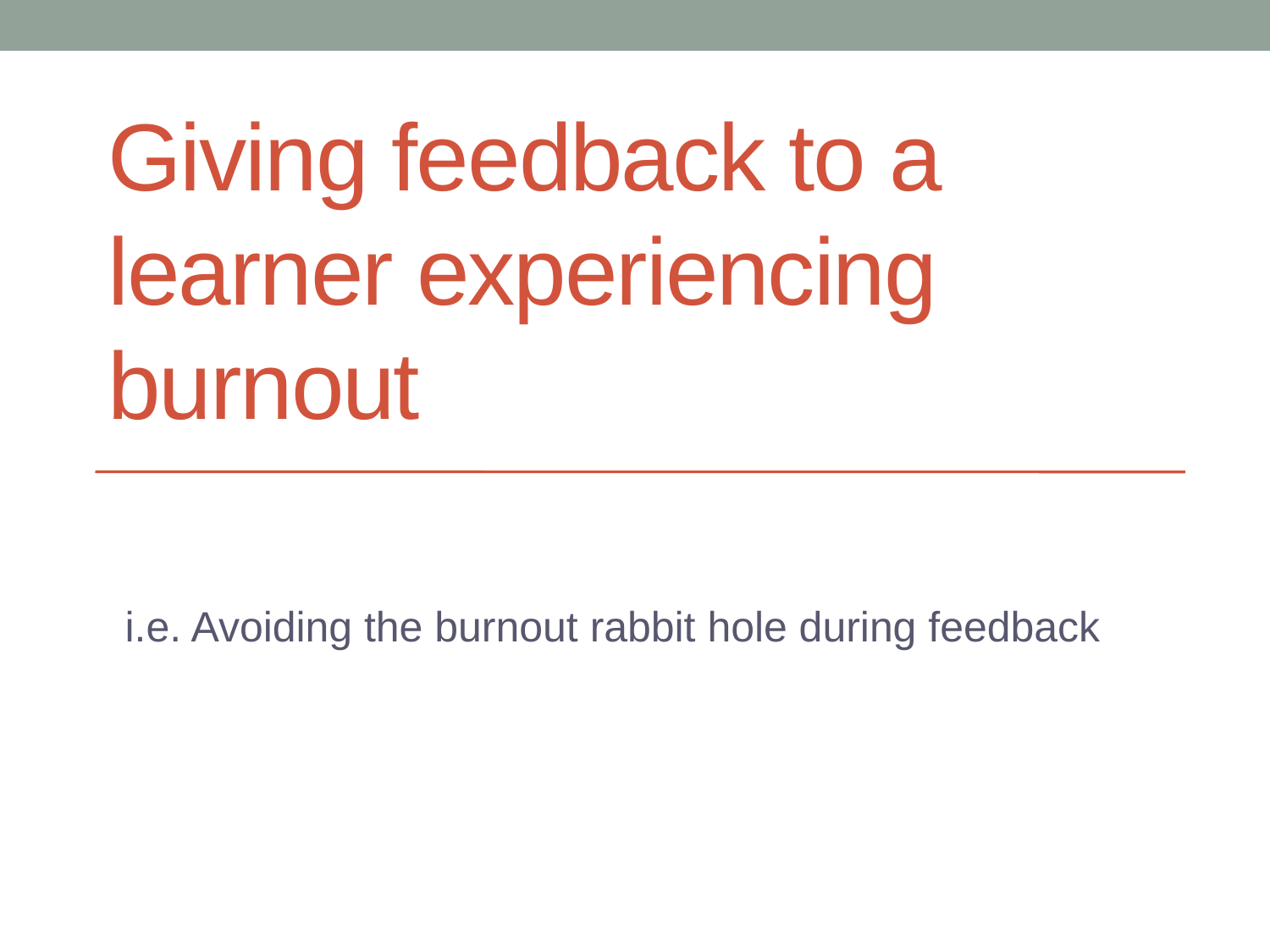

# Giving feedback to a learner experiencing burnout
i.e. Avoiding the burnout rabbit hole during feedback

## Slide 32
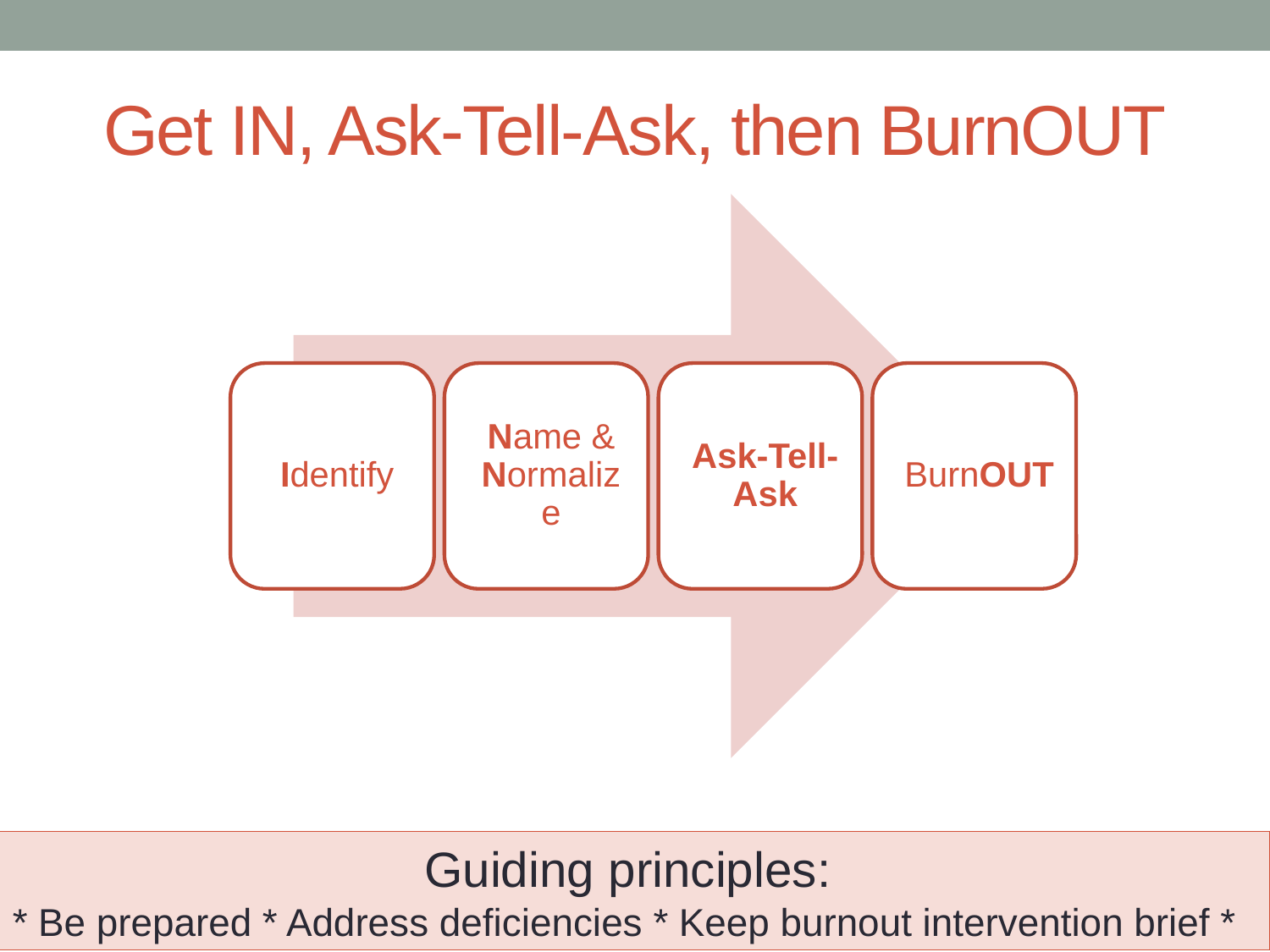

# Get IN, Ask-Tell-Ask, then BurnOUT
Guiding principles:
* Be prepared * Address deficiencies * Keep burnout intervention brief *

## Slide 33
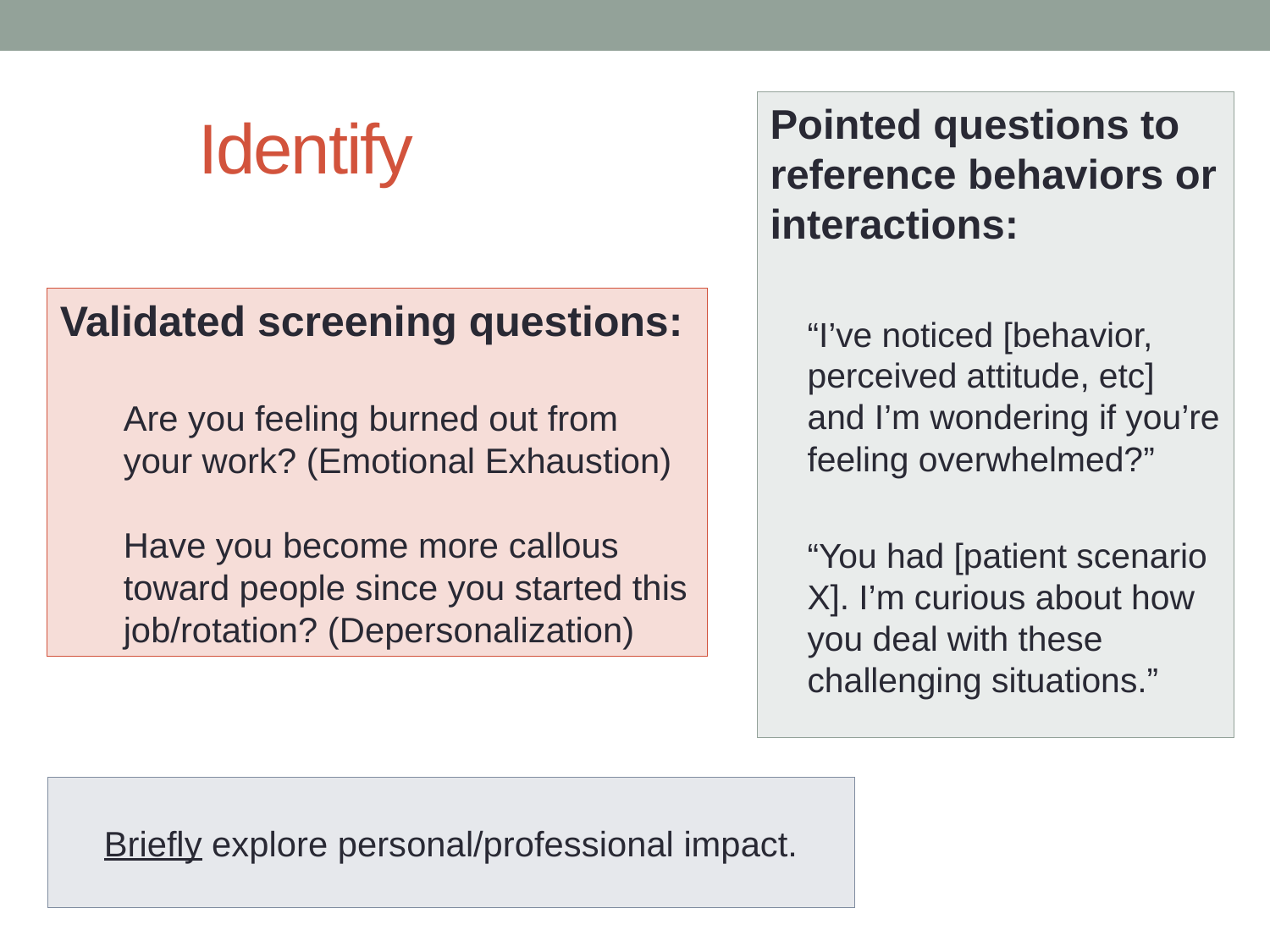

# Identify
Pointed questions to reference behaviors or interactions:
“I’ve noticed [behavior, perceived attitude, etc] and I’m wondering if you’re feeling overwhelmed?”
“You had [patient scenario X]. I’m curious about how you deal with these challenging situations.”
Validated screening questions:
Are you feeling burned out from your work? (Emotional Exhaustion)
Have you become more callous toward people since you started this job/rotation? (Depersonalization)
Briefly explore personal/professional impact.

## Slide 34
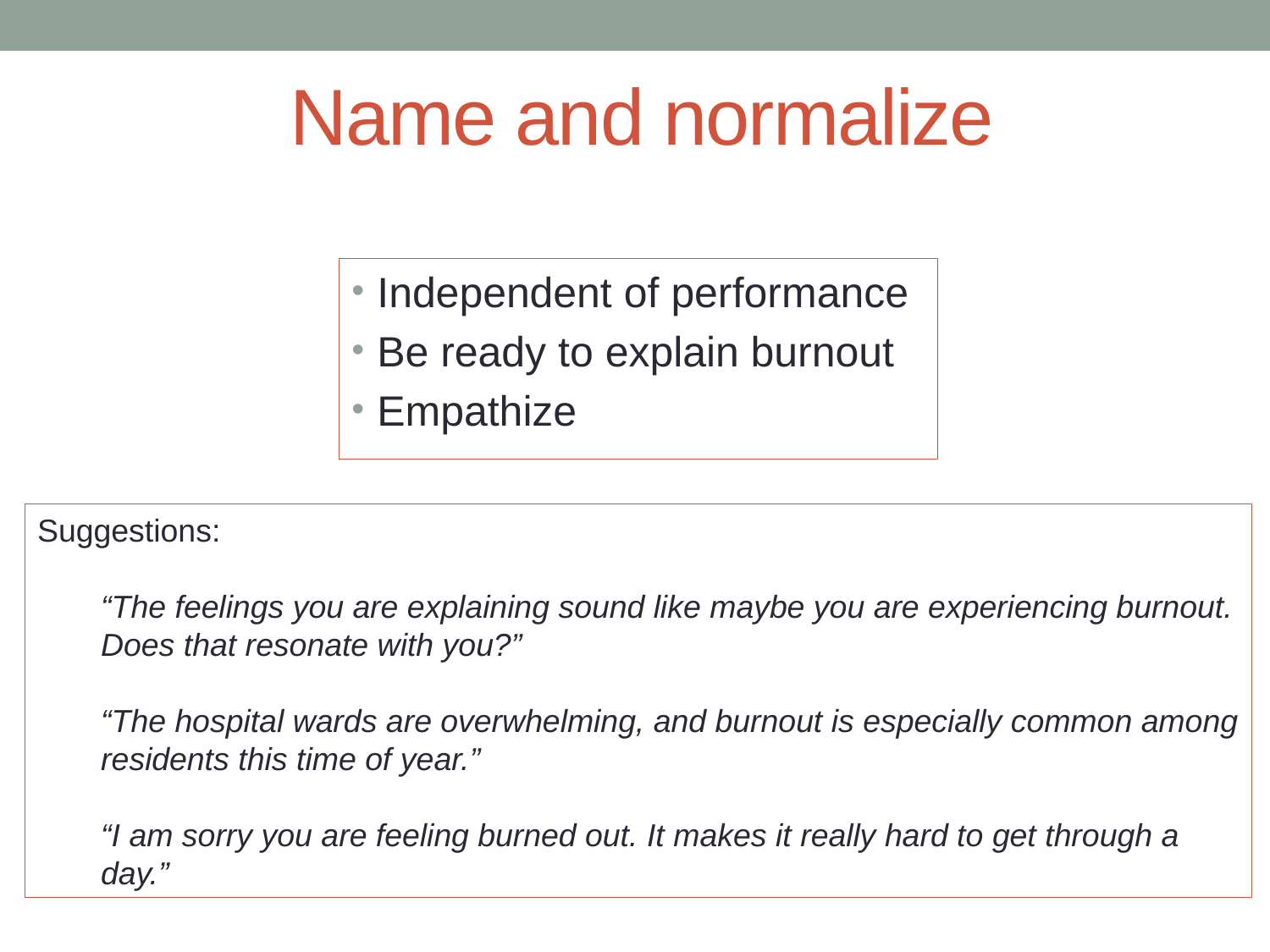

# Name and normalize
Independent of performance
Be ready to explain burnout
Empathize
Suggestions:
“The feelings you are explaining sound like maybe you are experiencing burnout. Does that resonate with you?”
“The hospital wards are overwhelming, and burnout is especially common among residents this time of year.”
“I am sorry you are feeling burned out. It makes it really hard to get through a day.”

## Slide 35
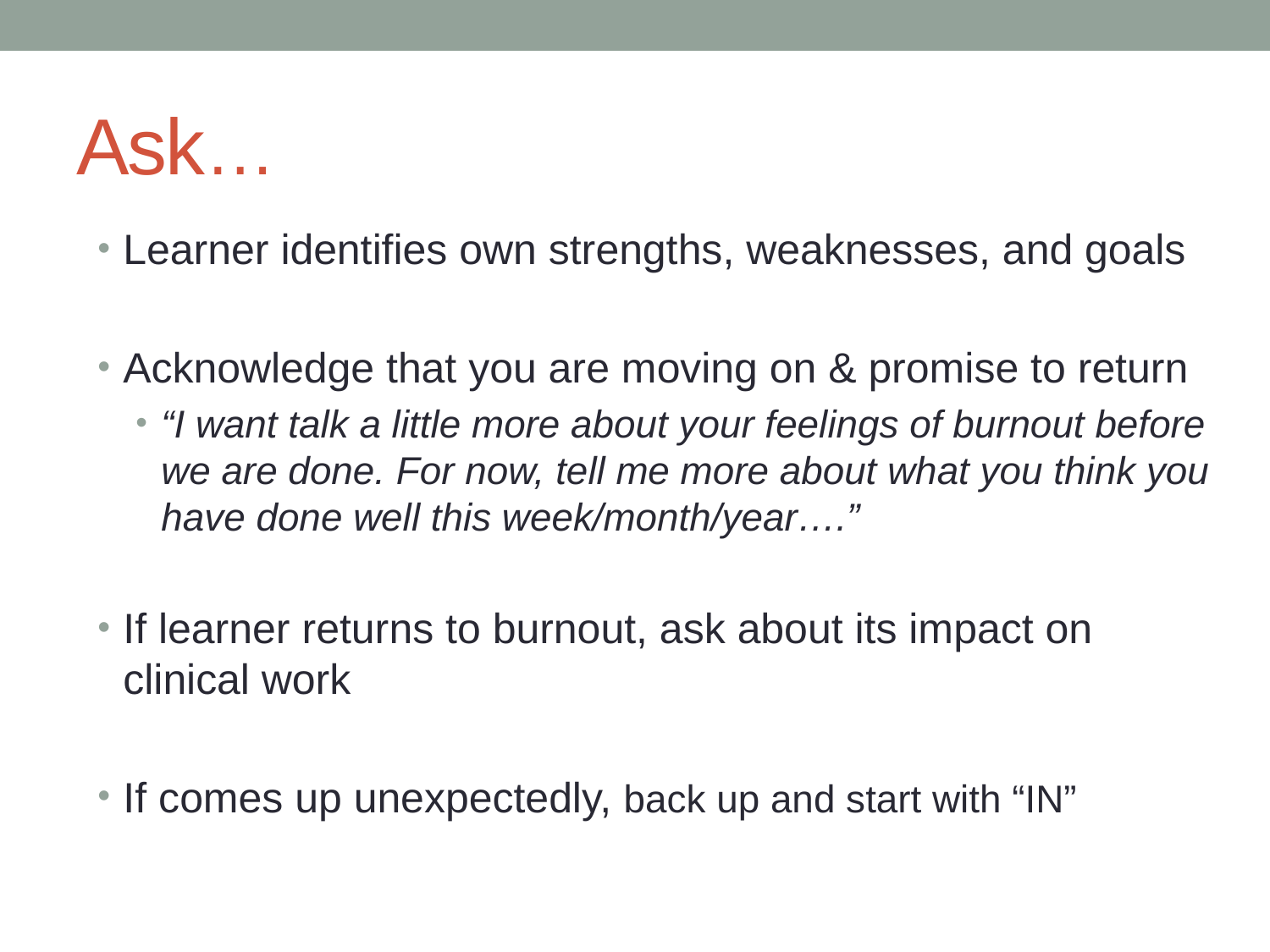

# Ask…
Learner identifies own strengths, weaknesses, and goals
Acknowledge that you are moving on & promise to return
“I want talk a little more about your feelings of burnout before we are done. For now, tell me more about what you think you have done well this week/month/year….”
If learner returns to burnout, ask about its impact on clinical work
If comes up unexpectedly, back up and start with “IN”

## Slide 36
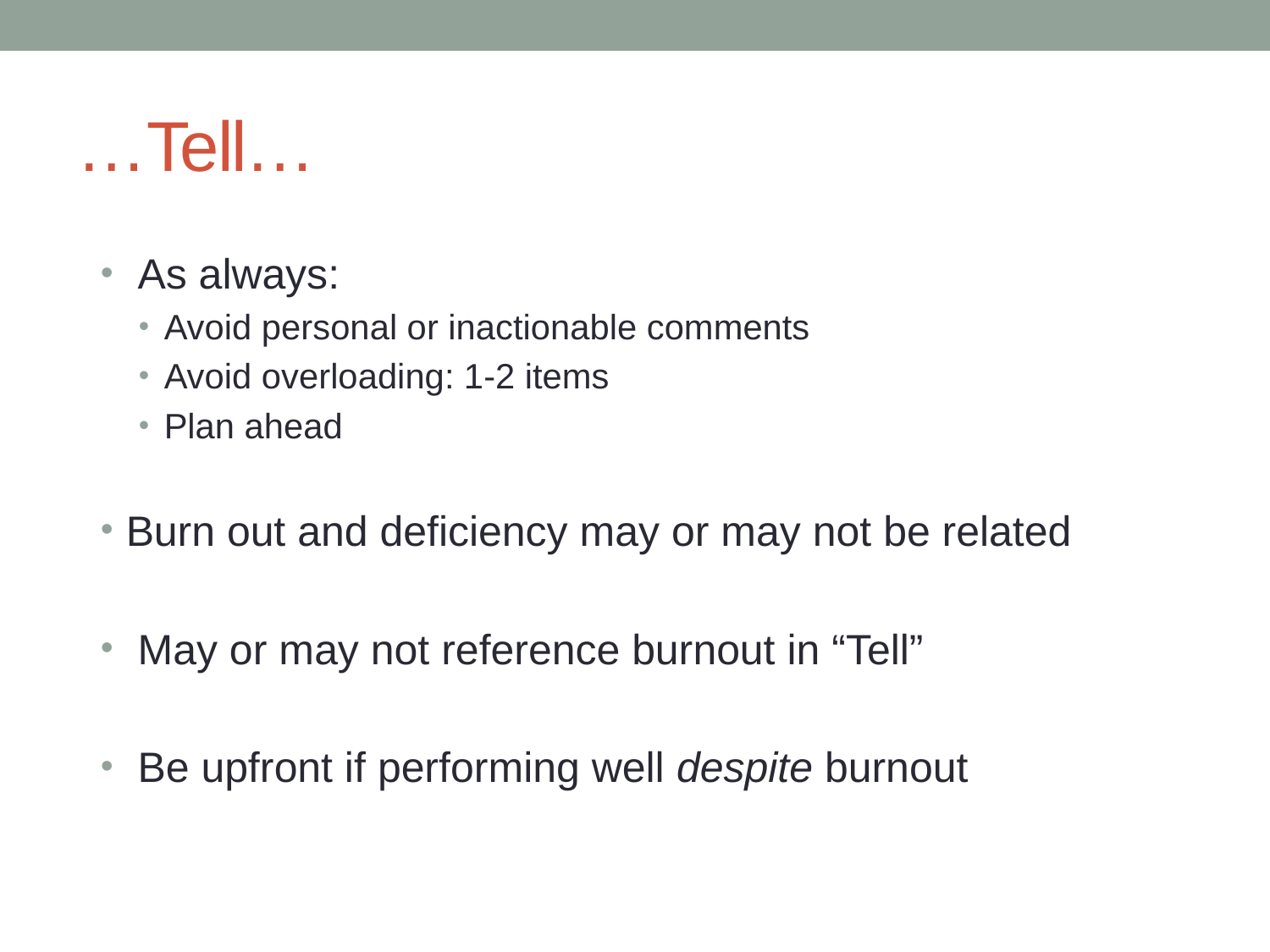

# …Tell…
 As always:
Avoid personal or inactionable comments
Avoid overloading: 1-2 items
Plan ahead
Burn out and deficiency may or may not be related
 May or may not reference burnout in “Tell”
 Be upfront if performing well despite burnout

## Slide 37
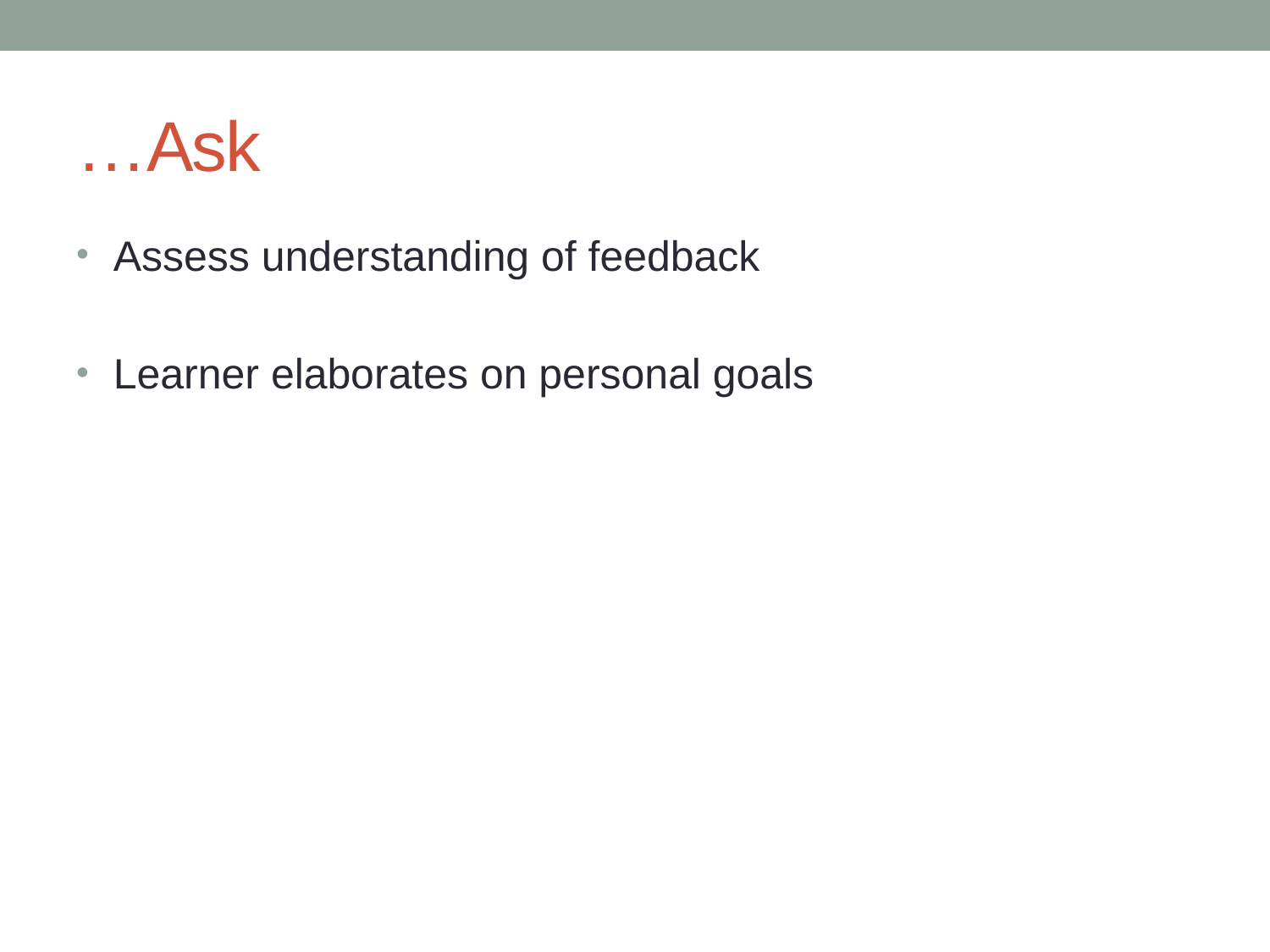

# …Ask
 Assess understanding of feedback
 Learner elaborates on personal goals

## Slide 38
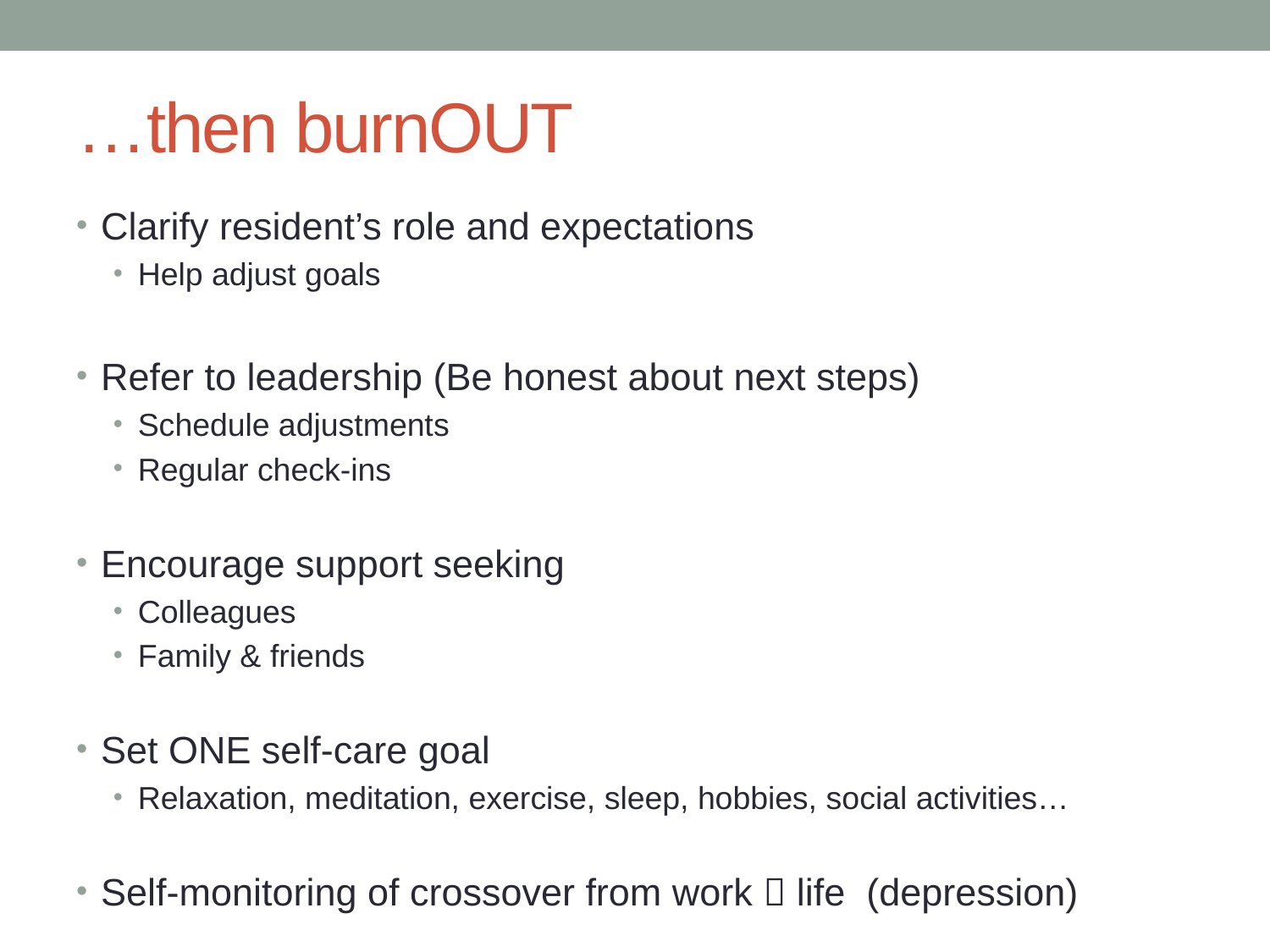

# …then burnOUT
Clarify resident’s role and expectations
Help adjust goals
Refer to leadership (Be honest about next steps)
Schedule adjustments
Regular check-ins
Encourage support seeking
Colleagues
Family & friends
Set ONE self-care goal
Relaxation, meditation, exercise, sleep, hobbies, social activities…
Self-monitoring of crossover from work  life (depression)

## Slide 39
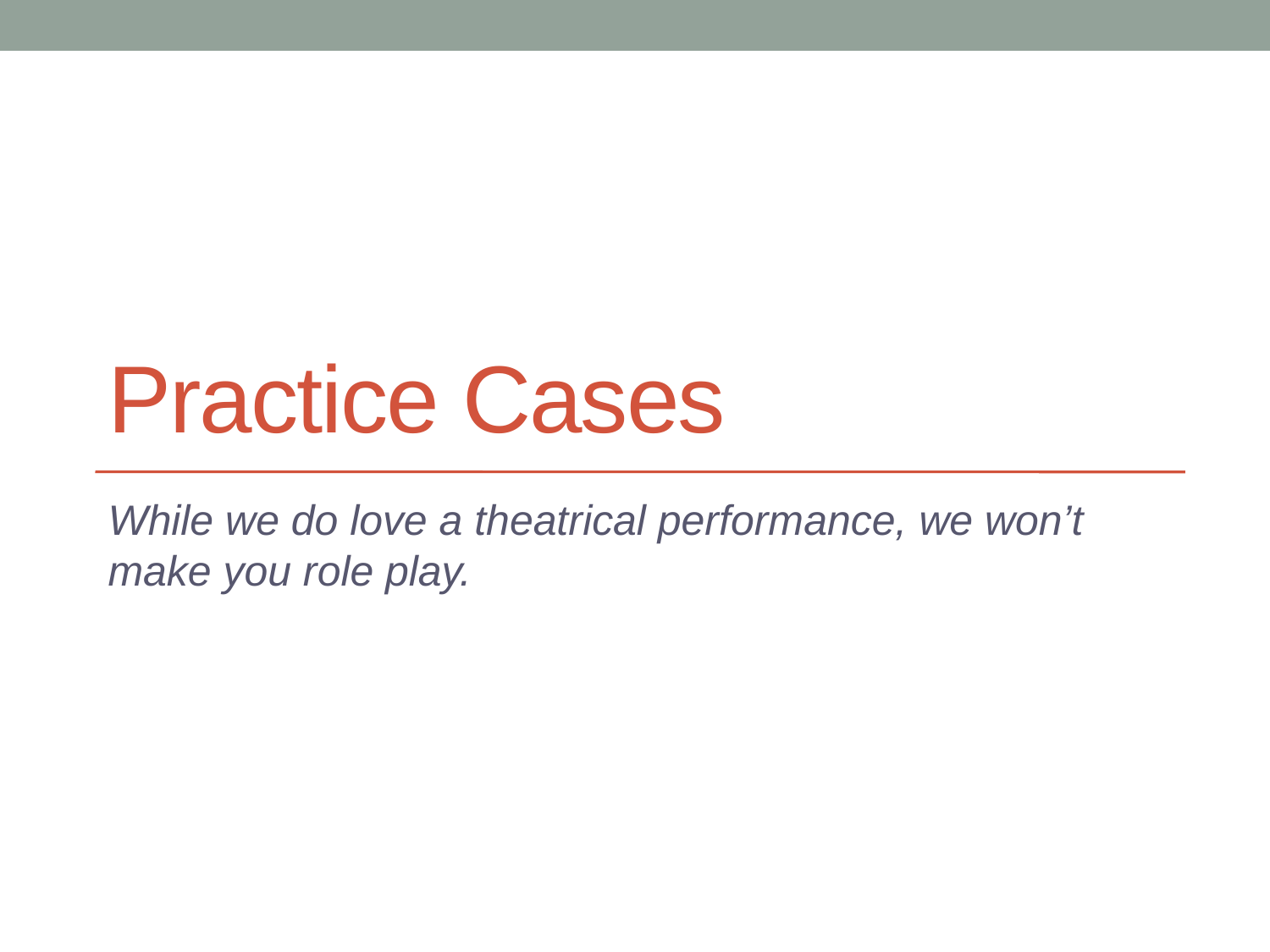

# Practice Cases
While we do love a theatrical performance, we won’t make you role play.

## Slide 40
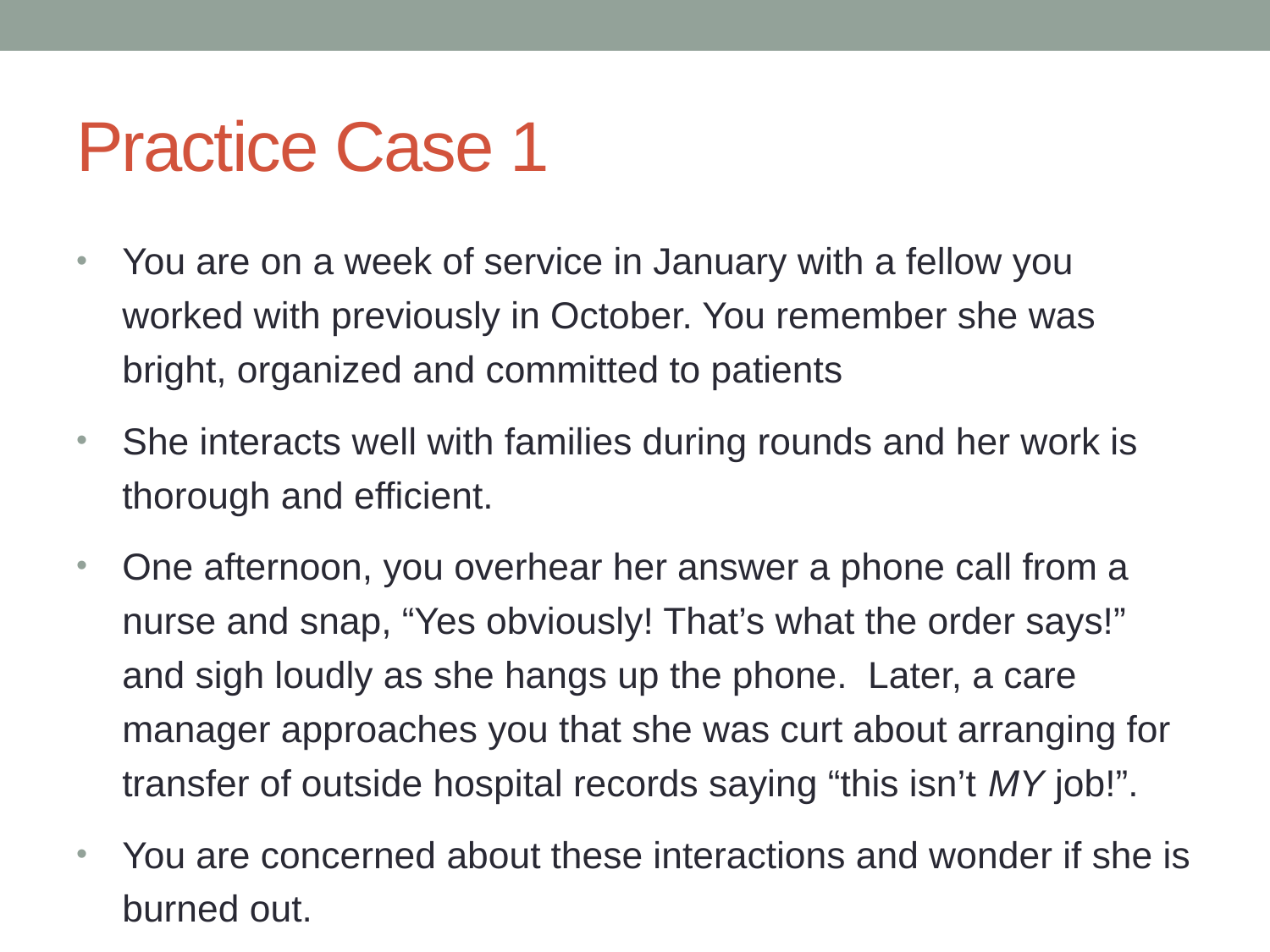

# Practice Case 1
You are on a week of service in January with a fellow you worked with previously in October. You remember she was bright, organized and committed to patients
She interacts well with families during rounds and her work is thorough and efficient.
One afternoon, you overhear her answer a phone call from a nurse and snap, “Yes obviously! That’s what the order says!” and sigh loudly as she hangs up the phone.  Later, a care manager approaches you that she was curt about arranging for transfer of outside hospital records saying “this isn’t MY job!”.
You are concerned about these interactions and wonder if she is burned out.

## Slide 41
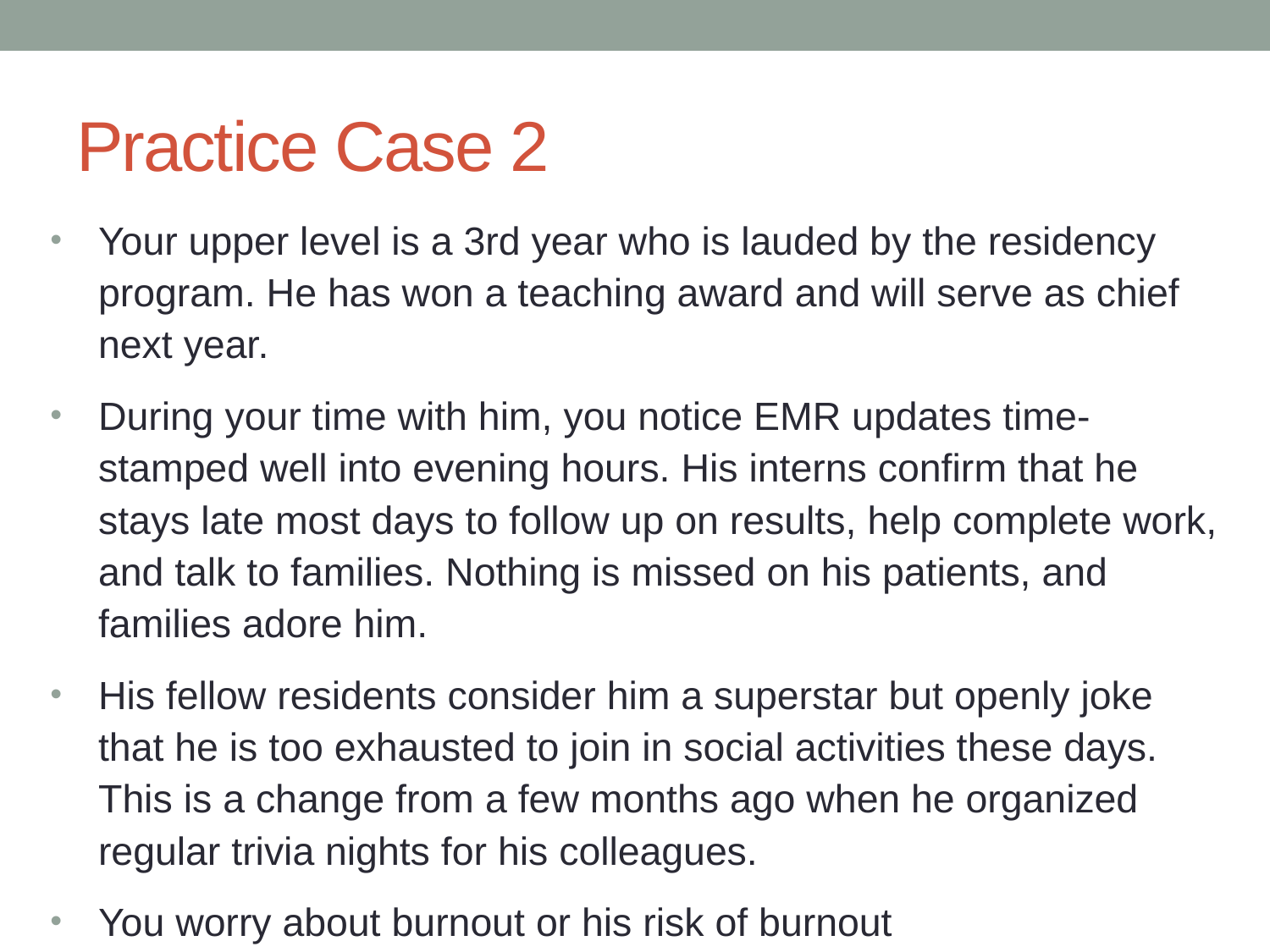

# Practice Case 2
Your upper level is a 3rd year who is lauded by the residency program. He has won a teaching award and will serve as chief next year.
During your time with him, you notice EMR updates time-stamped well into evening hours. His interns confirm that he stays late most days to follow up on results, help complete work, and talk to families. Nothing is missed on his patients, and families adore him.
His fellow residents consider him a superstar but openly joke that he is too exhausted to join in social activities these days. This is a change from a few months ago when he organized regular trivia nights for his colleagues.
You worry about burnout or his risk of burnout

## Slide 42
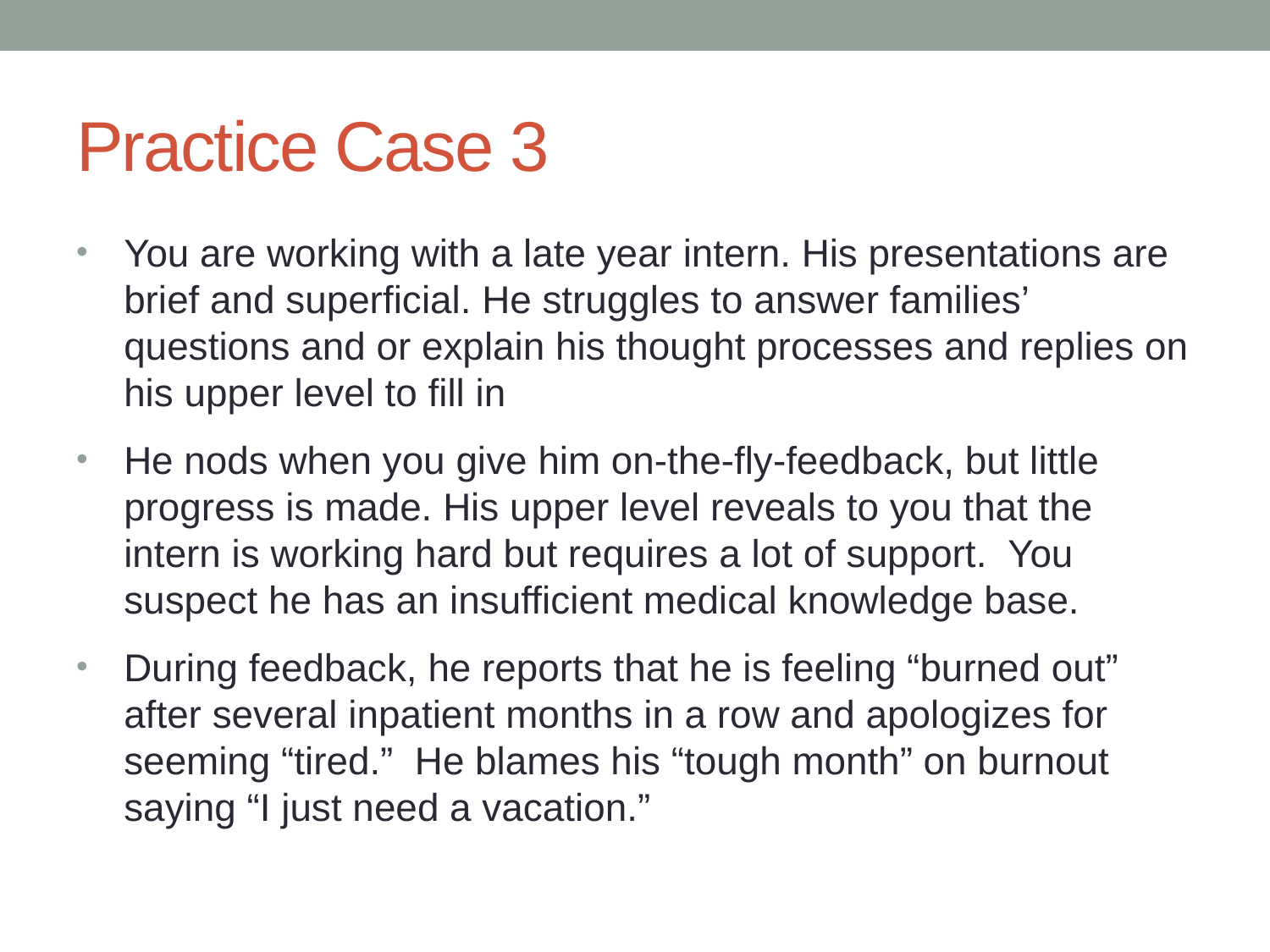

# Practice Case 3
You are working with a late year intern. His presentations are brief and superficial. He struggles to answer families’ questions and or explain his thought processes and replies on his upper level to fill in
He nods when you give him on-the-fly-feedback, but little progress is made. His upper level reveals to you that the intern is working hard but requires a lot of support.  You suspect he has an insufficient medical knowledge base.
During feedback, he reports that he is feeling “burned out” after several inpatient months in a row and apologizes for seeming “tired.”  He blames his “tough month” on burnout saying “I just need a vacation.”

## Slide 43
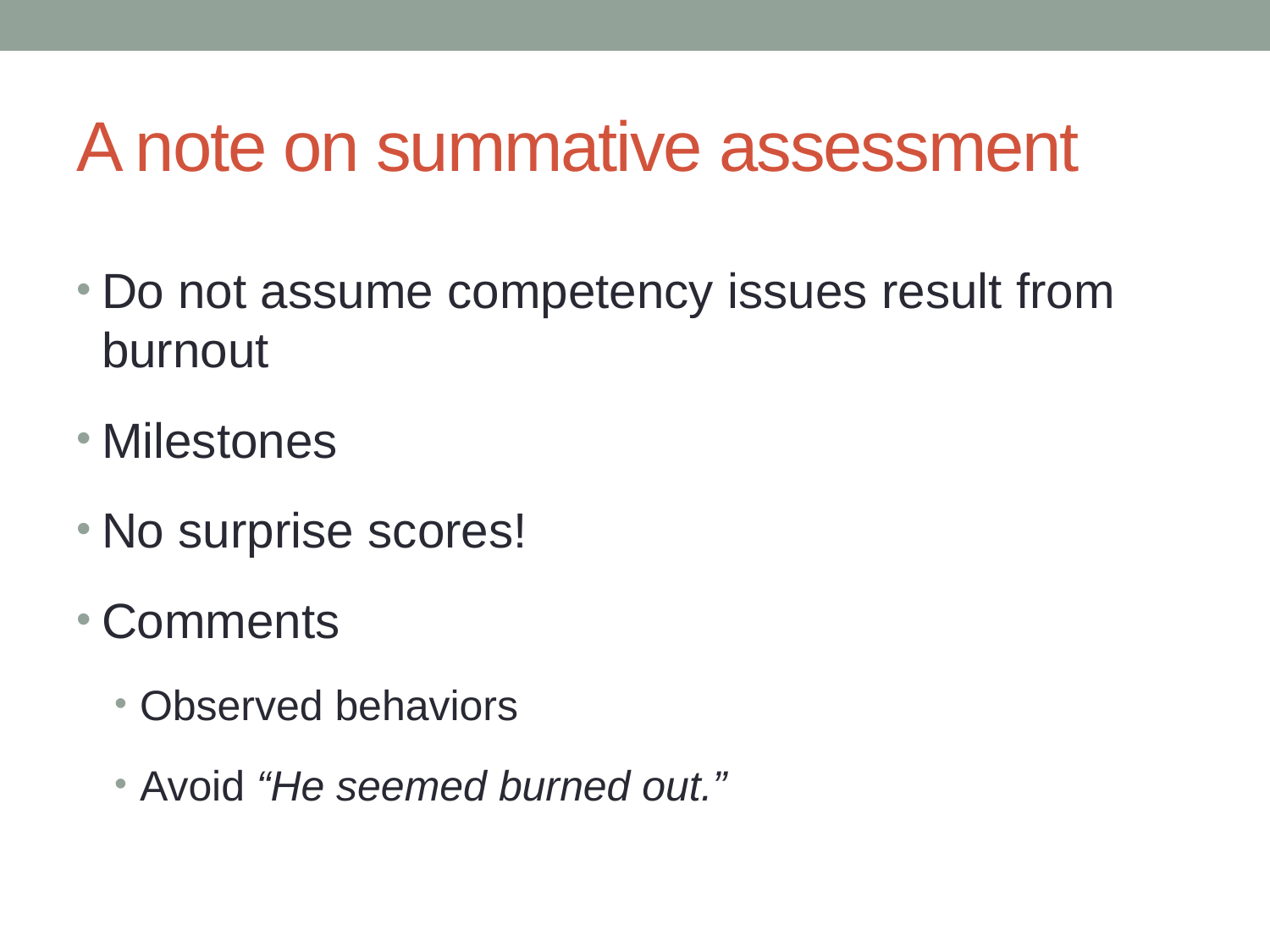

# A note on summative assessment
Do not assume competency issues result from burnout
Milestones
No surprise scores!
Comments
Observed behaviors
Avoid “He seemed burned out.”

## Slide 44
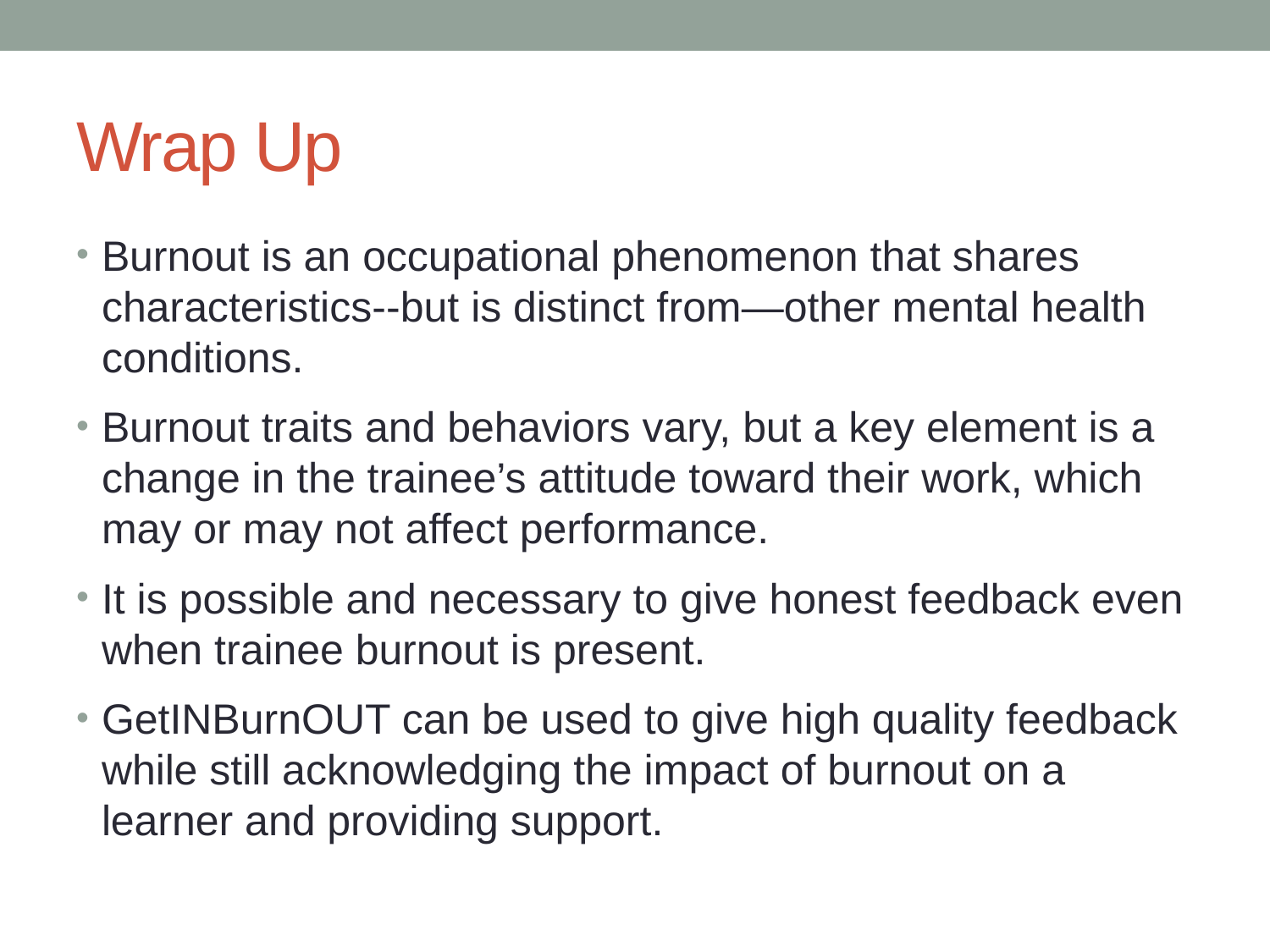

# Wrap Up
Burnout is an occupational phenomenon that shares characteristics--but is distinct from—other mental health conditions.
Burnout traits and behaviors vary, but a key element is a change in the trainee’s attitude toward their work, which may or may not affect performance.
It is possible and necessary to give honest feedback even when trainee burnout is present.
GetINBurnOUT can be used to give high quality feedback while still acknowledging the impact of burnout on a learner and providing support.

## Slide 45
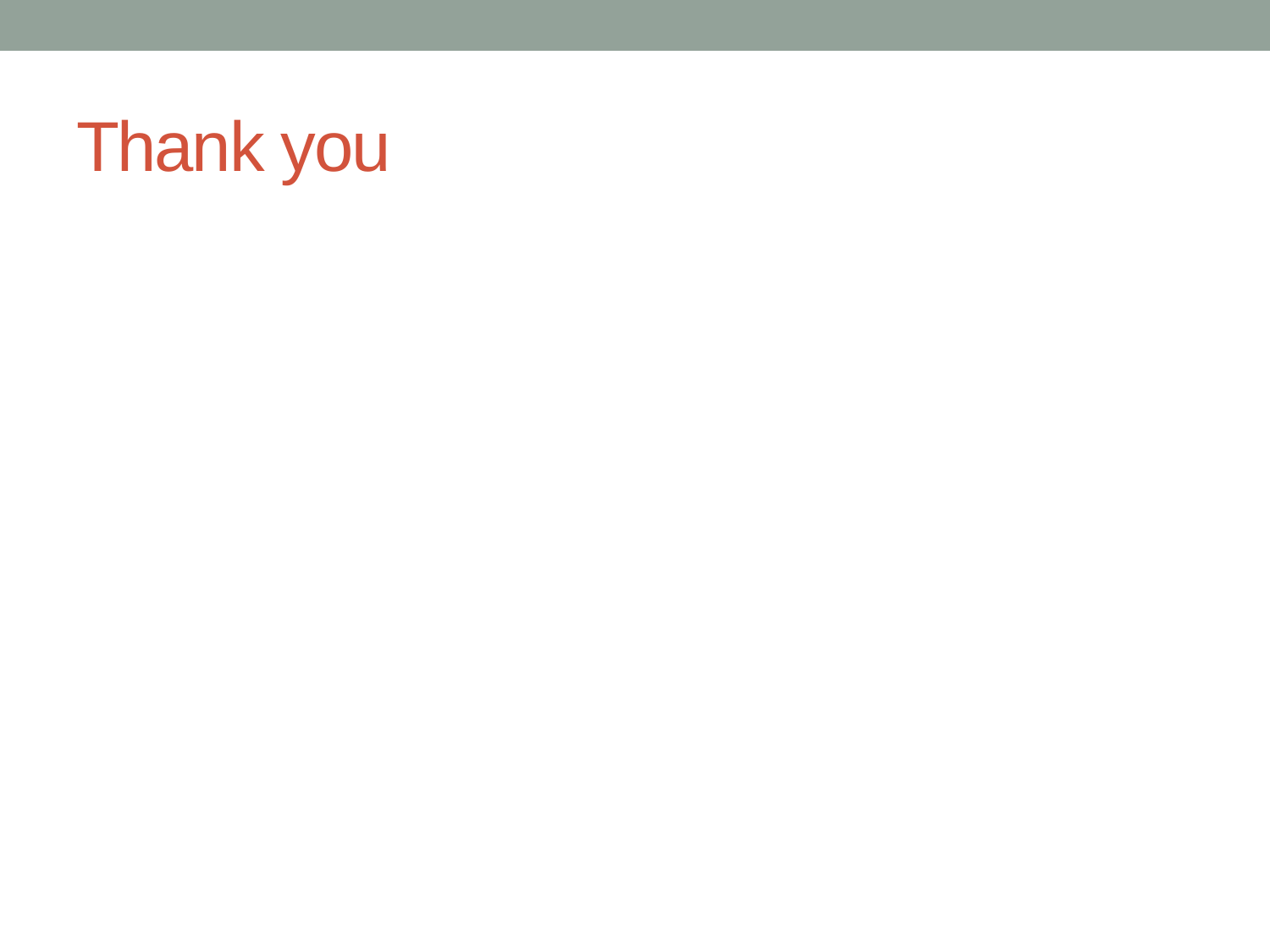

# Thank you
