# Supplementary material for: Balancing Honest Assessment and Compassion for Learners Experiencing Burnout: A Workshop and Feedback Tool for Clinical Teachers
Source: MedEdPORTAL. 2024 Oct 15;20:11449. doi: 10.15766/mep_2374-8265.11449 (PMC11473647; doi:10.15766/mep_2374-8265.11449)
Supplement: Supplementary file 1 — GetINburnOUT Method.pdfAgenda.docxFacilitator Guide.docxWorkshop Presentation.pptxCases.docxOnline Workshop Evaluation.pdf [file mep_2374-8265.11449-s001.zip › E. Cases.docx]

**CASE 1**

*What signs or behaviors of burnout is the learner exhibiting?*

*Is learner performance being affected by his/her burnout?*

*How can you use the GetINBurnOUT method to approach this learner? How will you incorporate honest and accurate feedback?*

- You are on a week of service in January with a resident you worked with previously in October. You remember she was bright, organized and committed to patients
- She interacts well with families during family centered rounds and her work is thorough and efficient.
- One afternoon, you overhear her answer a phone call from a nurse and snap, “Yes obviously! That’s what the order says!” and sigh loudly as she hangs up the phone.  Later, a care manager approaches you that she was curt about arranging for transfer of outside hospital records saying “this isn’t *MY* job!”.
- You are concerned about these interactions and wonder if she is burned out.

**CASE 2**

*What signs or behaviors of burnout is the learner exhibiting?*

*Is learner performance being affected by his/her burnout?*

*How can you use the GetINBurnOUT method to approach this learner? How will you incorporate honest and accurate feedback?*

- Your upper level is a 3^rd^ year who is lauded by the residency program. He has won a teaching award and will serve as chief next year.
- During your time with him, you notice EMR updates time-stamped well into evening hours and his interns confirm he stays late most days to follow up on results, help them complete work, and talk to families. Nothing is missed on his patients and families’ obviously adore him.
- His fellow residents consider him a superstar, but joke openly that he is too exhausted to join the group for social activities these days. This is a change from a few months ago when he organized regular trivia nights for his colleagues.
- You worry about burnout or his risk of burnout

**CASE 3**

*What signs or behaviors of burnout is the learner exhibiting?*

*Is learner performance being affected by his/her burnout?*

*How can you use the GetINBurnOUT method to approach this learner? How will you incorporate honest and accurate feedback?*

- You are working with a late year intern. His presentations are brief and superficial. He struggles to answer families’ questions and or explain his thought processes and replies on his upper level to fill in
- He nods when you give him on-the-fly-feedback, but little progress is made. His upper level reveals to you that the intern is working hard, but requires a lot of support.  You suspect he has an insufficient medical knowledge base.
- During feedback, he reports that he is feeling “burned out” after several inpatient months in a row and apologizes for seeming “tired.”  He blames his “tough month” on burnout saying “I just need a vacation.”
