# Supplementary material for: Balancing Honest Assessment and Compassion for Learners Experiencing Burnout: A Workshop and Feedback Tool for Clinical Teachers
Source: MedEdPORTAL. 2024 Oct 15;20:11449. doi: 10.15766/mep_2374-8265.11449 (PMC11473647; doi:10.15766/mep_2374-8265.11449)
Supplement: Supplementary file 1 — GetINburnOUT Method.pdfAgenda.docxFacilitator Guide.docxWorkshop Presentation.pptxCases.docxOnline Workshop Evaluation.pdf [file mep_2374-8265.11449-s001.zip › F. Online Workshop Evaluation.pdf]

Critiquing with compassion: Workshop Evaluation

Achievement of objectives

1. Please indicate how well each of the stated program objectives was achieved on a scale of 1 (poor) to 5 (excellent)

* Define burnout and differentiate it from other mental health conditions in physicians-in-training

1 (poor) 3 (neutral) 5 (excellent)

* Identify manifestations of burnout in learners on clinical rotations

1 (poor) 3 (neutral) 5 (excellent)

* Compare and contrast burnout and lack of competency in learners

1 (poor) 3 (neutral) 5 (excellent)

* Utilize the GetINburnOUT script to deliver accurate and constructive feedback to learners experiencing of burnout

1 (poor) 3 (neutral) 5 (excellent)

Critiquing with compassion: Workshop Evaluation

Overall presentation

Please evaluate the following elements of the overall presentation from 1 (poor) to 5 (excellent):

* Organization and Flow

1 (poor) 3 (fair) 5 (excellent)

* Utilization of interactive discussions and activities

1 (poor) 3 (neutral) 5 (excellent)

* Clarity of the slides and materials used to guide presentation

1 (poor) 3 (neutral) 5 (excellent)

* Usefulness of tools/handouts provided to participants

1 (poor) 3 (neutral) 5 (excellent)

* Time was allotted appropriately and allowed questions to be answered

1 (poor) 3 (neutral) 5 (excellent)

Critiquing with compassion: Workshop Evaluation

Personal expectations and practice

This activity met my personal expectations

- Yes
- No

This activity updated my current knowledge

- Yes
- No

I will use information gained in my future practice

- ◯ Yes
- ◯ No

Strengths of this session

Suggestions for Improvement

