# Supplementary material for: Balancing Honest Assessment and Compassion for Learners Experiencing Burnout: A Workshop and Feedback Tool for Clinical Teachers
Source: MedEdPORTAL. 2024 Oct 15;20:11449. doi: 10.15766/mep_2374-8265.11449 (PMC11473647; doi:10.15766/mep_2374-8265.11449)
Supplement: Supplementary file 1 — GetINburnOUT Method.pdfAgenda.docxFacilitator Guide.docxWorkshop Presentation.pptxCases.docxOnline Workshop Evaluation.pdf [file mep_2374-8265.11449-s001.zip › _Educational Summary Report_11449.pdf]

MedEdPORTAL® | The AAMC Journal of Teaching and Learning Resources

Original Publication Open Access

Balancing Honest Assessment and Compassion for Learners Experiencing Burnout: A Workshop and Feedback Tool for Clinical Teachers

Gal Barak, MD, MEd†, Dana Foradori, MD, MEd, H. Barrett Fromme, MD, MHPE, Linessa Zuniga, MD, MEd, Andrea Dean, MD*†

*Corresponding author: aldean@texaschildrens.org
†Co-primary author

Abstract

Introduction: Burnout in medical trainees challenges their work effectiveness and impedes their ability to learn. Teachers in the clinical learning environment (CLE) are well situated to identify burnout and are often responsible for learner assessment. **Methods:** We developed a workshop to improve clinical teachers' identification and understanding of learner burnout while empowering them to provide constructive feedback and support. Building on best-practice feedback techniques and utilizing the Maslach Burnout Inventory (MBI) as a framework, we designed the GetINBurnOUT method to provide feedback and support for learners experiencing burnout. Applying Kern's six-step approach to curriculum development, we created and implemented a workshop for clinical teachers centered on advanced burnout knowledge, application of the MBI to the CLE, and use of the GetINBurnOUT feedback method. Kolb's experiential learning theory informed workshop activities such as group discussion, case practice, and self-reflection. Participants completed surveys immediately after the workshop to assess planned behavior. **Results:** We delivered the workshop eight times at local, regional, and national faculty development programs/conferences to over 188 participants. Participants rated the workshop favorably, with average scores of 4.5-4.8 out of 5 across all domains and program objectives; all participants planned to make a change to their practice. Positive comments emphasized the topic's importance and the GetINBurnOUT tool's practicality. **Discussion:** This workshop can enhance clinical teachers' understanding of burnout and provide them with the tools to address it in the CLE. The GetINBurnOUT method offers a practical approach for providing honest assessments while supporting learners in the CLE.

Keywords
Burnout, Wellness, Assessment, Clinical Learning Environment, Faculty Development, Feedback, Mentoring/Coaching, Student Affairs, Well-Being/Mental Health

Educational Objectives

By the end of this activity, learners will be able to:

1. Define burnout and differentiate it from other mental health conditions in learners.
2. Identify manifestations of burnout in learners on clinical rotations.
3. Compare and contrast burnout and lack of competency in learners.
4. Utilize the GetINBurnOUT script to deliver accurate and constructive feedback to learners experiencing burnout.

Citation:
Barak G, Foradori D, Fromme HB, Zuniga L, Dean A. Balancing honest assessment and compassion for learners experiencing burnout: a workshop and feedback tool for clinical teachers. *MedEdPORTAL*. 2024;20:11449.
https://doi.org/10.15766/mep_2374-8265.11449

Introduction

Burnout, characterized by exhaustion, job-related cynicism, and reduced professional efficacy,[1] is at epidemic proportions among medical trainees.[2,3] In recent years, multiple organizations, including the Accreditation Council for Graduate Medical Education (ACGME), have increased requirements for support of trainee well-being.[4] The Clinical Learning Environment Review includes trainee well-being as one of six key focus areas.[5] Accordingly, institutions have incorporated a variety of programmatic interventions such as trainings and duty-hour regulations, as well as individual-centered interventions such as personal wellness planning, in an effort to combat rising burnout rates.

Copyright © 2024 Barak et al. This is an open-access publication distributed under the terms of the Creative Commons Attribution license.

Trainees on clinical rotations encounter the stressors inherent in caring for patients, and the threat of burnout is high. In our review of the literature, burnout interventions have largely focused on building resilience through mindfulness at the individual level or mitigating workplace demands at the program level. With the exception of a study on debriefing,[6] no burnout interventions specifically target the clinical learning environment (CLE) in real time.[7-9] Clinical teachers interface with trainees directly and are well situated to identify burnout and provide support, including in face-to-face feedback sessions during which the topic of burnout often arises. In our experience, learners may use this one-on-one opportunity to disclose their burnout to their supervisors or, when asked to reflect on their performance, their feelings of burnout become apparent. A needs assessment in 2022 by Dean and colleagues confirmed that clinician educators frequently experience this phenomenon yet, despite their frequency of working with burned-out learners, feel underprepared when they suspect or uncover burnout and feel limited in their ability to address burnout.[10]

With educators confronting learner burnout both in routine clinical practice and during feedback sessions, there is concern the quality of assessments is threatened. Accurate and objective assessment is a cornerstone of competency-based medical education (CBME) and includes formative assessment (i.e., feedback) to guide learning and move the trainee toward clinical competence,[11-15] as well as summative assessment, used as the basis for promotion of trainees. With CBME, it is important for educators to understand and attempt to overcome barriers to honest, objective, and accurate assessment including, but not limited to, internalized biases, time constraints, concerns about negative psychological consequences for learners and the educator themselves, tarnishing their reputation, lack of remediation options, or lack of institutional support if assessment is poorly received or the learner seeks retaliation.[13-18] Our needs assessment identified learner burnout as an additional barrier, as educators attempt to balance compassion and critique in their assessment of learners with whom they have time-constrained relationships and time-limited feedback sessions.[10]

As master clinical educators, our team of authors has undergone training in high-quality formative and summative assessment practices, yet we too have often struggled to deliver feedback when learner burnout is identified. We have found that time is limited once the topic of burnout dominates the conversation, and we are concerned that constructive feedback might exacerbate the learner's burnout. Our 2022 needs assessment confirmed a disconnect between intended versus actual assessments delivered to learners when burnout is present, primarily with a tendency toward leniency and sparing any more critical feedback.[10] Although, as clinical teachers, we agree that attention to psychological safety for learners is crucial and admirable, we also recognize that inaccurate and dishonest assessments mean missed opportunities to correct behavior and promote growth. Furthermore, given the prevalence of burnout, we are concerned that avoidance of critical assessment in the setting of burnout could contribute to the "failure to fail" phenomenon in which learners are prematurely promoted toward independent practice despite not actually achieving competency.[11,12,17]

With these high stakes in mind, our goal was to help clinical teachers identify and manage trainee burnout in busy CLEs and, more importantly, empower them to deliver honest and accurate assessment even when trainee burnout is suspected or confirmed. To do so, we created the GetINBurnOUT method, which merges principles of high-quality feedback with validated burnout screening questions and a brief, evidence-guided intervention. We then developed a curriculum in the form of a workshop focusing on high-level burnout knowledge acquisition and practice using the GetINBurnOUT technique.

Methods

We applied Kern's six-step approach[19] (Figure) to develop a brief, high-yield curriculum to teach on the topic of trainee burnout and disseminate the GetINBurnOUT feedback method (Appendix A). The educational project and evaluation were approved by the Baylor College of Medicine's Institutional Review Board (IRB H-49575).

Targeted Needs Assessment

We conducted a targeted needs assessment of clinical teachers to explore their experience and comfort with learner burnout in the CLE. A published study described the results of a web-based survey of clinical teachers. It found that clinical teachers frequently perceived burnout in their learners who reported neutral levels of comfort in identifying burnout. The study confirmed that the presence of burnout exacerbated known barriers to assessment and that frontline educators in the CLE altered their assessment of learners, mostly toward leniency, when burnout was present.[10]

Designing the GetINBurnOUT Method

Given a lack of existing tools, we developed the GetINBurnOUT feedback method for educators use with learners experiencing burnout. GetINBurnOUT acts as a mnemonic for the three-part feedback method: (1) IN, that is, identify, name, and

Copyright © 2024 Barak et al. This is an open-access publication distributed under the terms of the Creative Commons Attribution license.

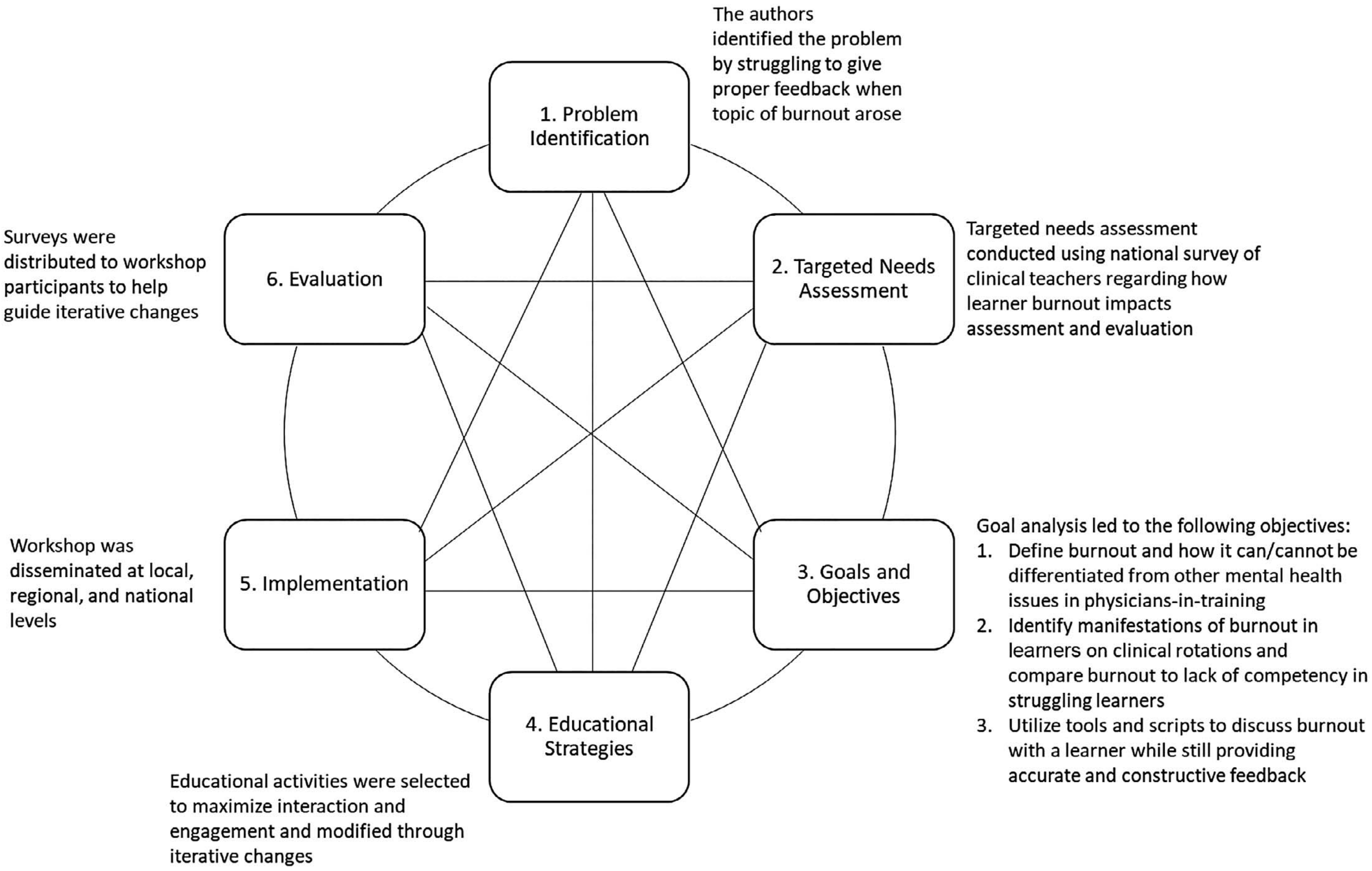

Figure. Kern's method of curriculum development was used to identify and fill an educational gap.

normalize; (2) deliver feedback; and (3) BurnOUT, that is, exit the session by returning to the topic of burnout and providing a brief intervention[20] (Appendix A). The method used validated screening questions derived from the Maslach Burnout Inventory (MBI) to identify burnout.[21,22] The method did not teach feedback techniques, instead emphasizing objective, behavior-based feedback[12,13,23] and utilizing scripting, which had been shown to increase confidence giving feedback.[24] Our wellness experts designed a brief five-step intervention based on wellness literature. It was designed to be used in the CLE and did not require special expertise, allowing widespread use by clinical teachers. Educators from multiple institutions piloted this method while on teaching service to confirm feasibility and acceptability.

Workshop Development

The workshop revolved around providing the GetINBurnOUT method. Additional components aimed to maximize the tool's impact, including improving burnout knowledge and identification. Educational goals and objectives were determined based on the needs assessment in keeping with Kern's approach.[19] Highly interactive strategies were selected according to Kolb's model of experiential learning.[25] Activities included personal reflection; small-group games, exercises, and discussion; large-group brainstorming; and case practice.

Audience

The target audience was clinical teachers. Of note, while the workshop was initially designed for pediatric inpatient teachers, the content was neither specialty nor venue specific. The workshop was designed for any clinical teachers interacting with learners in the CLE and responsible for the assessment of learners. No previous burnout knowledge was required to attend the session. However, most participants did have experience with feedback and assessment practices.

Facilitator Requirements

The session was best conducted by two to three facilitators, depending on the number of participants. Facilitators needed to have experience with providing feedback and to review the burnout content in detail to prepare.

Copyright © 2024 Barak et al. This is an open-access publication distributed under the terms of the Creative Commons Attribution license.

Setting and Materials

The workshop was initially designed as a 75-minute, in-person session and was subsequently revised into a virtual format ranging from 60 to 120 minutes. The final iteration, as provided in the agenda and facilitator guide (Appendices B and C), was a 90-minute, in-person workshop. Depending on group size and presenter preference, time could be adjusted for discussion and individual reflection. An abbreviated 60-minute session could be made possible by shortening discussions within each section.

To maximize collaboration, a room where participants could sit in groups of five to six was required. The PowerPoint presentation acted as a visual aid for presenters and audience alike (Appendix D), so the room had to be equipped with a projector and screen capable of displaying PowerPoint slides. A sound system was not needed. For each small group, large paper with a writing surface and markers were used in brainstorming activities. Red and green flags (i.e., pieces of paper) were used for the true/false game. Sticky notes were used for one activity. Based on feedback from participants, printout copies of the GetINBurnOUT method were provided in later iterations of this session. Cases were printed and distributed to tables (Appendix E).

For the virtual version, PollEverywhere software was utilized, but other polling software or chat function for true/false and open-answer text capability would suffice. The ability to break out into small groups was preferred for virtual case discussions.

Description of Workshop

The PowerPoint slides (Appendix D) and facilitator guide (Appendix C) moved the workshop through a series of interactive and didactic portions. The first half focused on building knowledge, dispensing myths, and establishing a personal connection to the material. After a break, the workshop focused on learner assessment in the setting of burnout and how to use the GetINBurnOUT method. Three cases (Appendix E) to practice application of the GetINBurnOUT method closed the workshop. Lastly, participants were offered the opportunity to evaluate the workshop.

Evaluation

Attendees completed anonymous paper evaluations at the pilot workshop at the Pediatric Hospital Medicine (PHM) national conference. Subsequently, all others were asked to complete an anonymous web-based evaluation on Survey Monkey (Appendix F). The customized online evaluation used a 5-point Likert-scale (1 = *poor,* 5 = *excellent,* or 1 = *strongly disagree,* 5 = *strongly agree*) to assess the workshop's effectiveness in meeting objectives, the quality of the presentation materials, and participants' intention for behavior change in response to the workshop. The evaluation also included open-ended questions soliciting comments about strengths and weaknesses of the training. No identifying information was collected. Likert-scale responses were analyzed with descriptive statistics to obtain the mean and standard deviation, and comments were reviewed for repeated content.

Results

The workshop has been delivered eight times in virtual and in-person formats ranging from 60 to 120 minutes in length (Table 1). It was selected by peer review process for presentation at local, regional, and national medical education conferences as well as by invitation at faculty development sessions. Approximately 188 total participants attended the workshop. Participants included pediatric faculty, institutional educational leaders, pediatric subspecialty fellows, and pediatric chief residents.

Table 1. Overview of Workshop Attendance and Evaluation Completion Rates

Conference	Date	Description of Venue	Format	Length	Participants	Evaluations: No. (%)
PHM Annual Meeting[a]	July 28, 2019	National conference	In person	75 minutes	31	10 (31)
BCM Annual Education Retreat	December 4, 2020	Local conference	Virtual	90 minutes	36	16 (44)
Association of Pediatric Program Directors Annual Meeting	March 26, 2021	National conference	Virtual	120 minutes	32	6 (19)
Texas Educator's Academies Collaborative for Health Professions	May 14, 2021	Regional conference	Virtual	60 minutes	28	14 (50)
Cleveland Clinic Faculty Development Series	July 6, 2021	Faculty development series	Virtual	60 minutes	10	4 (40)
BCM Division of PHM Faculty Development Series	August 24, 2021	Faculty development series	Virtual	60 minutes	25	8 (32)
Pediatric Academic Societies Annual Meeting	April 24, 2022	National conference	In person	90 minutes	26	13 (50)
Cleveland Clinic Faculty Development Series	October 12, 2023	Faculty development series	Virtual	60 minutes	4	3 (75)
Total					192	74 (38)
Total after pilot					161	64 (42)

Abbreviations: BCM, Baylor College of Medicine; PHM, Pediatric Hospital Medicine.
[a]The PHM 2019 national conference served as the pilot workshop session.

Copyright © 2024 Barak et al. This is an open-access publication distributed under the terms of the Creative Commons Attribution license.

Evaluations
A total of 10 (32%) of the 31 participants at the PHM annual meeting completed the paper evaluation after the pilot session. Participants rated the facilitators and workshop favorably, with means of 4.1-4.5 out of 5 across all domains on the evaluation, including meeting objectives, workshop elements, and overall activity. In addition, workshop scores were greater than or equal to the average score across all workshops presented at the conference.

Subsequently, 64 (42%) of 161 participants have completed the customized online evaluation. Results indicated that participants had a favorable response to the workshop elements, with average scores of 4.6-4.8 out of 5 across all domains (Table 2). Program objectives were met, with average scores of 4.5-4.7 out of 5, and 100% of participants indicated they would change their practice based on the workshop.

Comments from all evaluations were reviewed for repeated content. Of the 81 participants who evaluated the workshops, 46 (57%) left comments, which are summarized by theme in Table 3. These comments highlighted the utility and accessibility of the GetINBurnOUT method as a major strength of the workshop. In addition, participants appreciated the interactive nature of the workshop, including the use of sample cases.

Discussion

Burnout is present at alarming rates amongst medical trainees.[2,3] This workshop was designed to train educators to recognize and address burnout in trainees in the CLE and equip them with tools to balance critique and compassion in their assessments of learners experiencing burnout. It has been well received at local, regional, and national conferences and has been iteratively modified based on participant feedback to optimize engagement and learning for both in-person and virtual settings. Our team's particular attention to applying Kolb's experiential learning model[25] to the workshop structure to ensure active engagement throughout was particularly successful, with comments positively reflecting on the interactive nature of each portion. We continue to receive invitations to present, as well as requests to make the slides available for use by facilitators outside of our team. We are confident that interested individuals can use this publication to deliver the workshop.

Organizations such as the American Academy of Pediatrics and the ACGME have highlighted the importance of addressing burnout among medical trainees.[4,26] Prior efforts, including education and resources compiled at a national level, either target program directors, who may not directly interface with trainees in the CLE, or place the onus on trainees themselves to improve their own well-being through education.[7-9,27] Our curriculum and GetINBurnOUT method demonstrate how clinical teachers in the CLE can be incorporated into wellness interventions to address burnout among medical trainees in the setting where they are most likely to experience it. Based on a review of the literature in PubMed, *MedEdPORTAL,* and Web of Science, this is the first resource of its kind to capitalize on an existing expectation for educators to provide learners with feedback as well as intervene when burnout is uncovered.

In developing the GetINBurnOUT method, we wanted to create a hard stop for teachers to deviate from the discussion of burnout

Table 2. Summary of Evaluation Data (Excluding the 2019 Pediatric Hospital Medicine Conference)

Question	*M* (*SD*)	% Answering Yes
Program objectives[a]		
Define burnout and how it can/cannot be differentiated from other mental health issues in physicians-in-training	4.6 (0.6)	
Identify manifestations of burnout in learners on clinical rotations	4.7 (0.5)	
Compare and contrast burnout to lack of competency in learners	4.5 (0.6)	
Utilize the GetINBurnOUT script to deliver accurate and constructive feedback to learners experiencing burnout	4.6 (0.5)	
Workshop elements[a]		
Organization and flow	4.7 (0.5)	
Utilization of interactive discussions and activities	4.7 (0.5)	
Clarity of the slides and materials used to guide presentation	4.8 (0.5)	
Usefulness of tools/handouts provided to participants	4.6 (0.7)	
Time was allotted appropriately and allowed questions to be answered	4.7 (0.5)	
Overall activity[b]		
Met personal expectations		100
Updated current knowledge		100
I will use in my future practice		100

[a]Rated on a 5-point Likert scale (1 = *poor,* 3 = *neutral,* 5 = *excellent*).
[b]Rated as yes/no.

Copyright © 2024 Barak et al. This is an open-access publication distributed under the terms of the Creative Commons Attribution license.

Table 3. Summary of Survey Comments by Theme

Domain	Theme	Example Comments	
		Strengths of the Session	Suggestions for Improvement
Workshop flow	The workshop was organized effectively, though in shorter iterations was noted to be rushed.	"Great coordination of small group activities and lecture/didactic."	"At times felt a little rushed."
Burnout content	Workshop content, including defining burnout and identifying signs and symptoms of burnout in learners, was valuable and relevant for most participants.	"Really good overview of identifying burnout in learners."<br>"Expectations for addressing burnout while in the inpatient setting was a great perspective."	"I would appreciate far less time on definitions and background of burnout, and rather spend the whole time of feedback and addressing burnout."
Interactive activities	The workshop was designed to be interactive and promote active engagement with the material.	"Creating an interactive session without role play."<br>"Great avoidance of traditional lecture style, kept audience very engaged."	
GetINBurnOUT tool	Many participants found the feedback tool to be the most helpful part of the workshop.	"Concrete tools/framework to approach feedback to a learner experiencing burnout."<br>"Loved learning about this novel tool."<br>"This was a very helpful framework to follow. It's user-friendly and was well explained."	"Providing a separate worksheet handout with the feedback framework for participant to use when discussing cases."
Case examples	The case scenarios resonated with participants and allowed for application of the feedback tool; many participants wanted additional opportunities to practice with the tool either through more cases or the use of role-play.	"Great use of example cases."<br>"Practice using content with breakout sessions. When we are hearing information presented, it may seem easy to implement. However, when we actually practiced it, that was when I realized there were additional questions that I had to better implement the content."	"More time to actually implement tool."<br>"Maybe some role play would have been helpful as well."

and establish an opening for delivery of honest and accurate feedback. However, we knew that empathetic teachers would only feel empowered to do so if the method also provided support; thus, we aimed to include a burnout intervention. A literature review revealed no validated burnout interventions suitable to this time-limited, one-on-one setting. Therefore, our wellness experts designed an intervention, utilizing evidence and wellness literature indirectly in each of its steps; for example, a major contributor to workplace burnout is excessive workload,[1,28] so the intervention begins with role clarifying and resetting expectations for the learner in the given clinical setting. Support seeking and realistic self-care goals are also grounded in literature.[7-9,28,29] We gave special attention to practical use of the intervention in the CLE. Therefore, the method is not a comprehensive plan to mitigate burnout or evaluate for alternative mental health problems. Instead, the intervention incorporates a referral to program leadership, when necessary, and concludes with empowering the adult learner to self-monitor for signs of depression.[1,28,29] Its brevity makes it feasible and demonstrates a shift in the more common approach to trainee wellness from top-down program-level interventions to bottom-up individual attention.

While the GetINBurnOUT method requires minimal training, the intention of the workshop is to enhance the ability of the educator to use it by improving comfort with the topic of burnout. Our decision to teach beyond the application of the GetINBurnOUT method was supported by workshop evaluations that reported objectives fulfilled, knowledge and skills gained, and intention to apply information in respondents' teaching practice. However, in giving the workshop numerous times, we learned several lessons that led to iterative improvements included in the final version. First, participants' baseline knowledge of burnout was highly variable and often inaccurate, likely a result of burnout terminology being culturally mis- and overused. Taking the time to encourage the audience members to verbalize their reasoning during the true/false exercise allowed the facilitators to identify these misconceptions and guide their teaching throughout the workshop. Importantly, it also led to increased focus on building baseline knowledge of the MBI, the foundation for individuals interested in burnout and wellness across fields. This ensured consistent and accurate language surrounding burnout while using the tool. We learned that it was essential to devote time to this section to answer questions and ensure understanding.

One consistent challenge was delivering instruction on the complex interplay between burnout and depression, as well as the overlap of symptoms and signs.[1,28,29] This discussion often threatened to go beyond the scope of the workshop, and participants frequently expressed anxiety over their responsibility to identify mental health diagnoses in learners and how to deal with them. The facilitators overtly verbalized that this training and tool were for use in time-limited relationships in the CLE, not to teach comprehensive psychiatric diagnostics or the nuances of system-level support for mentally ill learners. We often moved

Copyright © 2024 Barak et al. This is an open-access publication distributed under the terms of the Creative Commons Attribution license.

on from this discussion by emphasizing that any concerns, whether for burnout or for ensuring attention to other coexisting mental health issues, should be escalated to program leadership immediately.

Audience participation and discussion were enhanced in the in-person format compared to virtually. We found that virtual sessions, while still receiving positive evaluations regarding the interactive nature of the workshop, had less discussion. It was more difficult to gauge audience level of knowledge during the true/false activity, and the discussion around burnout behavior was more superficial. In person, there was robust interest in differentiating behaviors within each domain of the MBI. As such, the virtual version tended to be shorter.

There are recognized limitations to this work. First, our evaluation tool mainly measured the reaction of the participants to the workshop. While this is a low tier of the Kirkpatrick model,[30] our results still demonstrate the feasibility of this innovation for audiences of varying expertise and disciplines. Although objective longitudinal data about the use of GetINBurnOUT in practice proved challenging to collect, participants reported that the tool was practical and requested copies of it for their reference or to make badge cards (Appendix A). Ongoing invitations to present from former participants have also provided subjective feedback on the tool's usefulness in practice.

Another limitation is that this feedback tool and workshop, although briefly reviewing best practice feedback techniques, do little to negate baseline barriers to feedback, such as time constraints and negative consequences for the learners and educators if assessment is poorly received.[13-18] Therefore, if a specific participant has not been trained in feedback techniques and is struggling to deliver feedback even when burnout is not present, this intervention may not be effective.

This workshop has been presented in a variety of settings to participants with various backgrounds. Although the workshop has been presented primarily to pediatric educators, participants in a variety of subspecialties with different educational roles and backgrounds have responded positively to our curriculum and feedback tool. This suggests that the materials are applicable to a wide audience range and could be generalizable beyond the field of pediatrics and to multidisciplinary medical teachers, such as nursing or advanced practice providers. At the time of publication, this intervention has only been delivered to self-selected audience members and therefore may miss the educators most in need of its type of training unless future parties make it mandatory.

In addition to ongoing dissemination, our next step is to explore how the workshop and GetINBurnOUT method can be applied to other domains of graduate or undergraduate medical education. With ACGME Milestones 2.0 incorporating well-being as a component of professionalism, this may have significant relevance at the programmatic level.[4] Future studies can explore learners' perceptions of the feedback method and its impact on their well-being. Furthermore, we would like to understand how burnout impacts assessments beyond the CLE, particularly with regard to CBME and promotion. We believe the principles outlined in the workshop, including the feedback tool, could be adapted to improve assessment practices in other settings, namely, clinical competency committees. Overall, this faculty development workshop and feedback tool are important steps in acknowledging and addressing the impact of burnout on medical trainees and present one approach to balancing support and honest feedback for learners experiencing burnout.

Appendices

A. GetINburnOUT Method.pdf
B. Agenda.docx
C. Facilitator Guide.docx
D. Workshop Presentation.pptx
E. Cases.docx
F. Online Workshop Evaluation.pdf

All appendices are peer reviewed as integral parts of the Original Publication.

Gal Barak, MD, MEd: Assistant Professor, Department of Pediatrics, Baylor College of Medicine

Dana Foradori, MD, MEd: Assistant Professor, Department of Pediatrics, Cleveland Clinic Lerner College of Medicine

H. Barrett Fromme, MD, MHPE: Professor, Department of Pediatrics, University of Chicago Pritzker School of Medicine

Linessa Zuniga, MD, MEd: Assistant Professor, Department of Pediatrics, Baylor College of Medicine

Andrea Dean, MD: Associate Professor, Department of Pediatrics, Baylor College of Medicine; ORCID: https://orcid.org/0000-0002-8522-0234

Disclosures
None to report.

Funding/Support
None to report.

Copyright © 2024 Barak et al. This is an open-access publication distributed under the terms of the Creative Commons Attribution license.

Prior Presentations

Dean A, Barak G, Zuniga L, Fromme HB, Foradori D. Enabling educators balance honest assessment and compassion for burned out learners. Poster presented at: Pediatric Academic Society Meeting; April 25, 2022; Denver, CO.

Dean A, Barak G, Zuniga L, Fromme HB, Foradori D. Enabling educators balance honest assessment and compassion for burned out learners. Oral abstract presented at: Association of Pediatric Program Directors; May 18, 2022; San Diego, CA.

Ethical Approval

The Baylor College of Medicine Institutional Review Board approved this project.

References

1. Burn-out an "occupational phenomenon": International Classification of Diseases. World Health Organization. May 28, 2019. Accessed September 26, 2024. https://www.who.int/news/item/28-05-2019-burn-out-an-occupational-phenomenon-international-classification-of-diseases
2. Dyrbye LN, West CP, Satele D, et al. Burnout among U.S. medical students, residents, and early career physicians relative to the general U.S. population. *Acad Med*. 2014;89(3):443-451. https://doi.org/10.1097/ACM.0000000000000134
3. IsHak WW, Lederer S, Mandili C, et al. Burnout during residency training: a literature review. *J Grad Med Educ*. 2009;1(2):236-242. https://doi.org/10.4300/JGME-D-09-00054.1
4. *ACGME Common Program Requirements (Residency)*. Accreditation Council for Graduate Medical Education; 2023. Accessed September 26, 2024. https://www.acgme.org/globalassets/pfassets/programrequirements/cprresidency_2023.pdf
5. CLER Evaluation Committee. *CLER Pathways to Excellence: Expectations for an Optimal Clinical Learning Environment to Achieve Safe and High-Quality Patient Care, Version 3.0.* Accreditation Council for Graduate Medical Education; 2024. Accessed September 26, 2024. https://doi.org/10.35425/ACGME.0010
6. Gunasingam N, Burns K, Edwards J, Dinh M, Walton M. Reducing stress and burnout in junior doctors: the impact of debriefing sessions. *Postgrad Med J*. 2015;91(1074):182-187. https://doi.org/10.1136/postgradmedj-2014-132847
7. West CP, Dyrbye LN, Erwin PJ, Shanafelt TD. Interventions to prevent and reduce physician burnout: a systematic review and meta-analysis. *Lancet*. 2016;388(10057):2272-2281. https://doi.org/10.1016/S0140-6736(16)31279-X
8. Zhang XJ, Song Y, Jiang T, Ding N, Shi TY. Interventions to reduce burnout of physicians and nurses: an overview of systematic reviews and meta-analyses. *Medicine (Baltimore)*. 2020;99(26):e20992. https://doi.org/10.1097/MD.0000000000020992
9. Panagioti M, Panagopoulou E, Bower P, et al. Controlled interventions to reduce burnout in physicians: a systematic review and meta-analysis. *JAMA Intern Med*. 2017;177(2):195-205. https://doi.org/10.1001/jamainternmed.2016.7674
10. Dean A, Foradori DM, Kumar S, et al. How perceived burnout alters frontline educators' assessments in the clinical learning environment. *Acad Pediatr*. 2022;22(3):495-500. https://doi.org/10.1016/j.acap.2021.12.014
11. Iobst WF, Sherbino J, Ten Cate O, et al.; International CBME Collaborators. Competency-based medical education in postgraduate medical education. *Med Teach*. 2010;32(8):651-656. https://doi.org/10.3109/0142159X.2010.500709
12. Lockyer J, Carraccio C, Chan MK, et al.; ICBME Collaborators. Core principles of assessment in competency-based medical education. *Med Teach*. 2017;39(6):609-616. https://doi.org/10.1080/0142159X.2017.1315082
13. Ende J. Feedback in clinical medical education. *JAMA*. 1983;250(6):777-781. https://doi.org/10.1001/jama.250.6.777
14. Bing-You R, Hayes V, Varaklis K, Trowbridge R, Kemp H, McKelvy D. Feedback for learners in medical education: what is known? A scoping review. *Acad Med*. 2017;92(9):1346-1354. https://doi.org/10.1097/ACM.0000000000001578
15. McQueen SA, Petrisor B, Bhandari M, Fahim C, McKinnon V, Sonnadara RR. Examining the barriers to meaningful assessment and feedback in medical training. *Am J Surg*. 2016;211(2):464-475. https://doi.org/10.1016/j.amjsurg.2015.10.002
16. Ramani S, Könings KD, Mann KV, Pisarski EE, van der Vleuten CPM. About politeness, face, and feedback: exploring resident and faculty perceptions of how institutional feedback culture influences feedback practices. *Acad Med*. 2018;93(9):1348-1358. https://doi.org/10.1097/ACM.0000000000002193
17. Yepes-Rios M, Dudek N, Duboyce R, Curtis J, Allard RJ, Varpio L. The failure to fail underperforming trainees in health professions education: a BEME systematic review: BEME Guide no. 42. *Med Teach*. 2016;38(11):1092-1099. https://doi.org/10.1080/0142159X.2016.1215414
18. Scarff CE, Bearman M, Chiavaroli N, Trumble S. Keeping mum in clinical supervision: private thoughts and public judgements. *Med Educ*. 2019;53(2):133-142. https://doi.org/10.1111/medu.13728
19. Thomas PA, Kern DE, Hughes MT, Tackett SA, Chen BY, eds. *Curriculum Development for Medical Education: A Six-Step Approach*. 4th ed. Johns Hopkins University Press; 2022.
20. Dean A, Fromme HB, Barak G, Zuniga L, Foradori D. GetINBurnOUT: feedback and support for trainees experiencing burnout. *Med Educ*. 2022;56(5):562-563. https://doi.org/10.1111/medu.14774
21. Kemper KJ, Wilson PM, Schwartz A, et al.; Pediatric Resident Burnout-Resilience Study Consortium. Burnout in pediatric residents: comparing brief screening questions to the Maslach Burnout Inventory. *Acad Pediatr*. 2019;19(3):251-255. https://doi.org/10.1016/j.acap.2018.11.003

Copyright © 2024 Barak et al. This is an open-access publication distributed under the terms of the Creative Commons Attribution license.

22. Li-Sauerwine S, Rebillot K, Melamed M, Addo N, Lin M. A 2-question summative score correlates with the Maslach Burnout Inventory. *West J Emerg Med*. 2020;21(3):610-617. https://doi.org/10.5811/westjem.2020.2.45139

23. Johnson CE, Keating JL, Boud DJ, et al. Identifying educator behaviours for high quality verbal feedback in health professions education: literature review and expert refinement. *BMC Med Educ*. 2016;16:96. https://doi.org/10.1186/s12909-016-0613-5

24. Fromme HB, Ryan MS, Gray K, et al. A script for what ails your learners: feedback scripts to promote effective learning. *Acad Pediatr*. 2020;20(5):721-723. https://doi.org/10.1016/j.acap.2020.02.005

25. Kolb DA. *Experiential Learning: Experience as the Source of Learning and Development*. 2nd ed. Pearson Education; 2015.

26. McClafferty H, Brown OW; Section on Integrative Medicine; Committee on Practice and Ambulatory Medicine. Physician health and wellness. *Pediatrics*. 2014;134(4):830-835. https://doi.org/10.1542/peds.2014-2278

27. Sullivan AG, Hoffman A, Slavin S. Becoming AWARE: ACGME's new suite of well-being resources. *J Grad Med Educ*. 2020;12(1):122-124. https://doi.org/10.4300/JGME-D-19-00967.1

28. West CP, Dyrbye LN, Shanafelt TD. Physician burnout: contributors, consequences and solutions. *J Intern Med*. 2018;283(6):516-529. https://doi.org/10.1111/joim.12752

29. Dyrbye L, Shanafelt T. A narrative review on burnout experienced by medical students and residents. *Med Educ*. 2016;50(1):132-149. https://doi.org/10.1111/medu.12927

30. Kirkpatrick DL. The four levels of evaluation. In: Brown SM, Seidner CJ, eds. *Evaluating Corporate Training: Models and Issues. Springer*; 1998:95-12. Accessed September 26, 2024. https://doi.org/10.1007/978-94-011-4850-4_5

Received: December 8, 2023
Accepted: May 20, 2024
Published: October 15, 2024

Copyright © 2024 Barak et al. This is an open-access publication distributed under the terms of the Creative Commons Attribution license.
